# A Novel Small‐Molecule Dual Adiponectin Receptor 1/2 Agonist Induces Myogenesis and Ameliorates Skeletal Muscle Atrophy

**DOI:** 10.1002/jcsm.70364

**Published:** 2026-08-24

**Authors:** Shubhrajyoti Das, Pallavi Awasthi, Md. Rameez Moin, Shyamal Pal, Anushka Talukdar, Akhilesh Kumar, Nikhil Nikolas, Shruti Suhas Varode, Shamima Khatoon, Nabanita Das, Sourav Chattopadhyay, Madhav Nilkanth Mugale, Rajdeep Guha, Sabyasachi Sanyal, Atul Goel

**Affiliations:** ^1^ Division of Biochemistry and Structural Biology CSIR‐Central Drug Research Institute Lucknow Uttar Pradesh India; ^2^ Academy of Scientific and Innovative research (AcSIR) Ghaziabad India; ^3^ Division of Medicinal and Process Chemistry CSIR‐Central Drug Research Institute Lucknow Uttar Pradesh India; ^4^ Division of Toxicology and Experimental Medicine CSIR‐Central Drug Research Institute Lucknow Uttar Pradesh India; ^5^ Laboratory Animal Facility CSIR‐Central Drug Research Institute Lucknow Uttar Pradesh India

**Keywords:** adiponectin receptor agonist, medicarpin, myofibre, sciatic nerve denervation, skeletal muscle atrophy

## Abstract

**Background:**

Skeletal muscle atrophy is a hallmark of ageing and chronic diseases, yet effective pharmacotherapies remain unavailable. Adiponectin signalling through AdipoR1 and AdipoR2 regulates skeletal muscle metabolism, regeneration and oxidative capacity, but the therapeutic use of adiponectin is limited by its large size and complex multimeric structure. Small‐molecule AdipoR agonists, therefore, represent an attractive therapeutic strategy.

**Methods:**

A PGC‐1α promoter‐luciferase assay was used to screen for AdipoR agonists in HEK‐293T cells overexpressing AdipoR1 or AdipoR2. Receptor specificity was validated through receptor overexpression and RNA‐interference. C2C12 myoblast differentiation was assessed using phase‐contrast microscopy and myosin heavy chain (MyHC) immunostaining followed by morphometric analyses of cross‐sectional area (CSA) and Feret's diameter, along with immunoblotting of MyoD and myogenin. Anti‐atrophy effects were examined in myotubes exposed to dexamethasone, cytokines or nutrient deprivation by evaluating CSA and diameter through morphometry, immunoblotting and qRT‐PCR analysis of atrogenes and myogenic markers. Oxidative metabolism and mitochondrial function were analysed using extracellular flux analysis and immunoblotting of fibre‐type markers, including MyHC‐I, MyHC‐IIA and MyHC‐IIB. In vivo efficacy was evaluated in a dexamethasone‐induced and a sciatic nerve denervation‐induced rat models following oral administration of Med. Muscle histology, molecular signalling and functional performance were analysed. All immunoblots were analysed by densitometry.

**Results:**

Med activated AdipoR1 and AdipoR2 with EC_50_ values of 160 and 302 pM, respectively, and stimulated canonical adiponectin signalling pathways, including AMPK, AKT and p38 MAPK. It significantly induced expression of typical adiponectin targets, PGC‐1α, PPARα, Glut4 and UCP3 (*p* < 0.0001). AdipoR1 knockdown completely abolished Med‐mediated signalling, whereas AdipoR2 depletion caused partial attenuation. Med enhanced myogenic differentiation, evidenced by increasing myotube formation and expression of MyoD, myogenin and MyHC. It protected myotubes against dexamethasone‐, cytokine‐ and nutrient deprivation‐induced atrophy by preserving CSA and Feret's diameter (*p* < 0.0001), which was accompanied by suppression of Atrogin‐1 and MuRF1, and increased MyoD and myogenin expression (*p* < 0.05). Med also enhanced oxidative capacity by increasing fatty acid oxidation and Med‐treated cells showed elevated oxidative fibre markers. Oral administration of Med significantly attenuated muscle atrophy in both rat models, evidenced by improved muscle morphology, suppressed atrogenes, enhanced myogenic markers and increased muscle adiponectin expression and corresponding downstream signalling. Med markedly improved muscle function, including grip strength (*p* < 0.0001), wire hanging (*p* < 0.01) and rotarod performance (*p* < 0.01) and toe‐spread ability of denervated limbs (*p* < 0.01).

**Conclusion:**

These findings identify Med as a potent small‐molecule orally bioavailable AdipoR agonist and provide proof‐of‐concept for AdipoR agonists as potential therapeutics for sarcopenia and muscle wasting disorders.

## 1 Introduction

Skeletal muscle atrophy is a pathological condition arising from an imbalance between protein degradation and synthesis and is characterized by induction of atrogenes, including the E3 ubiquitin ligases Atrogin‐1 and MuRF1 [1]. Muscle atrophy commonly accompanies chronic diseases and metabolic disorders, occurs as an adverse effect of various pharmacological agents, including corticosteroids and statins, and develops with ageing, anorexia, injury, disuse and denervation [1, 2, 3, S1–S3]. Currently, no pharmacological therapy is approved for the prevention or treatment of skeletal muscle atrophy [1, 2]. Exercise and nutritional interventions remain the only standard‐of‐care options, yet their feasibility is limited in elderly, frail or bedridden patients [4, 5] underscoring a critical unmet clinical need.

Multiple therapeutic strategies are under clinical evaluation, including hormonal therapies, appetite stimulants, inhibitors/neutralizing antibodies of inflammatory cytokines or activin receptors, β‐adrenergic receptor agonists, angiotensin‐converting enzyme inhibitors and cyclooxygenase‐2/prostaglandin E inhibitors [6, 7, 8, S4]. However, many of these approaches are constrained by cardiovascular liabilities, safety concerns or limited efficacy [6, 9].

Adiponectin is a systemically abundant cytokine primarily synthesized and secreted by adipocytes and skeletal muscles [10, S5]. Reduced serum adiponectin is clinically associated with metabolic diseases/disorders that include obesity, insulin resistance, Type 2 diabetes, atherosclerosis, various cancers and MASLD [10, 11, S5]. Adiponectin primarily signals through its membrane receptors AdipoR1 and AdipoR2, with AdipoR1 highly expressed in skeletal muscle and AdipoR2 predominantly expressed in the liver but also in muscle [10, 11, 12, S5]. Adiponectin, through its receptors, modulates glucose and lipid metabolism, inflammation, mitochondrial biogenesis and function, reactive oxygen species (ROS) and autophagy [10, 11, 12, S5, S6]. The primary effectors of adiponectin‐AdipoR signalling are rapid and transient AMPK, p38 and AKT activation, which leads to activation and induction of classical adiponectin targets, including PPAR‐gamma coactivator 1 alpha (PGC‐1α), glucose transporter 4 (Glut4), uncoupling proteins (UCP), fatty acid transporter protein/CD36, sirtuin 1 (Sirt1) and peroxisome proliferator‐activated receptor 1 alpha (PPARα) [11, 13]. Dysregulation of any of these pathways leads to metabolic disorders [10, S5–S7]. These make adiponectin or its receptors key targets for metabolic diseases/disorders.

Despite clear proof of concept, adiponectin‐replenishment therapy has not been feasible owing to its large molecular size and complex multimerization [14, S6]. Consequently, considerable effort has focused on the discovery of small‐molecule, orally available AdipoR agonists. To date, five such compounds have been reported following the initial discoveries by us and the Kadowaki group [13, 15]. Although our compound GTDF demonstrated receptor binding and efficacy at nanomolar concentration [13], its translation was hindered by challenges in bulk synthesis and the endangered status of its natural source, *Ulmus wallichiana*. Adiporon, although biologically active, requires high micromolar concentrations and exhibits toxicity in rabbits and potential off‐target liabilities [15, 16, S8]. Recently, ESME and AdipoAI have been described as AdipoR agonists; however, these agents display activity at micromolar concentrations and remain incompletely characterized [17, 18]. Isovitexin, identified as an AdipoR2 agonist [19], lacks intellectual property potential, which precludes its translation. Despite limitations, all these compounds display impressive preclinical efficacy against various pathophysiological conditions, which highlights the therapeutic promise and the persistent need for AdipoR agonists with improved efficacy, pharmacokinetics and safety profiles.

The importance of adiponectin signalling in skeletal muscle is well established. Adiponectin‐ and AdipoR1‐deficient mice exhibit impaired contractility, reduced oxidative fibre content and endurance [20]. Over the past decade, we and others have demonstrated protective and regenerative effects of adiponectin and small‐molecule AdipoR agonists in musculoskeletal disorders [21, 22], including skeletal muscle atrophy [12, 23, 24], through mechanisms involving enhanced myogenesis [12, 23], suppression of inflammation‐induced myolysis [S11], autophagy [25], improvement of muscular metabolism [13, 23] and mitochondrial oxidative capacity [23, 24, 26, S9, S10].

Here, we report the discovery and pharmacological characterization of medicarpin, a previously described osteogenic phytoalexin [27], and its water‐soluble sodium salt (Med), as dual AdipoR1/R2 agonists, and demonstrate the efficacy of Med in vitro and in rodent models of skeletal muscle atrophy.

## 2 Materials and Methods

### 2.1 Chemicals and Reagents

Medicarpin and Med (purity > 99%) were synthesized as before [28] (details in Data S1). Globular adiponectin (gAd) was synthesized and purified as reported [29]. Adiporon was from Abcam. Cell culture reagents were from Thermo Fisher Scientific and Sigma‐Aldrich. Fine chemicals were from Sigma‐Aldrich, Cayman, BioVision, Abcam and MP Biomedicals (Navi Mumbai, India). Antibody details are in Table S1. Stock solutions of medicarpin, forskolin and inhibitors were made in DMSO. Med stock solution was made in 20% ethanol. gAd was dissolved in sterile phosphate buffer saline (PBS; at 1 mg/mL). Final concentration of DMSO in all experiments was 0.1%, ethanol: 0.0002% and PBS: 0.001%. All the vehicles (V) at their final concentration were compared in pilot experiments in HEK‐293T and C2C12 cells, and because no significant difference was observed, the final diluent, DMEM‐high glucose (DMEM‐HG, Thermo Fisher Scientific), was used as vehicle in all in vitro experiments.

### 2.2 Cell Culture

C2C12 (CRL‐1772), HEK‐293T (CRL‐3216) were from ATCC, and cultured in growth medium (GM): DMEM‐HG supplemented with 10% FBS + 1X antibiotic‐antimycotic solution (Thermo Fisher Scientific), at 37°C in a 5% CO_2_ incubator. C2C12 cells between 2nd and 10th passages and HEK‐293T between 12th and 18th passages were used.

#### 2.2.1 C2C12 Myoblast Differentiation

The 90% confluent C2C12 myoblasts were incubated in serum‐free DMEM‐HG containing Med/gAd/Adiporon. Serum‐free DMEM‐HG (SS) and differentiation medium (DM; DMEM‐HG + 2% horse serum (HS); Thermo Fisher Scientific) served as negative and positive controls, respectively. Treatments were replenished every alternate day. Microscopy and immunoblotting were performed on the third day.

#### 2.2.2 AdipoR Over‐Expression and Luciferase Reporter Assays in HEK‐293 Cells

AdipoR over‐expression and luciferase reporter assays in HEK‐293 cells were performed as earlier [19] and in the Supporting Information.

#### 2.2.3 AdipoR Knockdown in C2C12 Myotubes, qRT‐PCR and Immunoblotting

AdipoR knockdown in C2C12 myotubes, qRT‐PCR and immunoblotting are detailed in the Supporting Information. Full immunoblot images are given in the Supporting Information. The qRT‐PCR primer sequences are in Table S2.

### 2.3 Immunofluorescence Staining and Morphometric Analyses

C2C12 myoblasts on cover slips inside 12‐well plates were treated with SS, SS supplemented with test compounds or DM for 3 days. Myotubes were fixed in 10% neutral buffered formalin (NBF), permeabilized with 1% Triton X‐100 (Sigma Aldrich), blocked with 1% BSA, Fraction V (MP Biomedicals), and incubated with MyHC antibody (MF‐20), overnight at 4°C. After probing with Alexa Fluor‐488‐conjugated secondary antibody (Thermo Fisher Scientific), nuclei were stained with DAPI in mounting medium (Abcam). Images were captured using a Nikon ECLIPSE Ts2 microscope at 100× magnification, and Nikon NIS‐Elements F 5.21.00 software was used to acquire and merge the fluorescent channels. Morphometry was performed using the ImageJ software (Version 1.54d, NIH) [S12]. MyHC^+^ cells with elongated tubular shape and ≥ 5 nuclei/cell were used for quantification of area and diameter (*n* = 3, 18 fields/group, 10 myotubes/field, total 180 myotubes/treatment group). Fusion index was calculated as before [S13] using the formula: fusion index (%) = 100 × (number of nuclei in MyHC^+^ myotubes)/(total number of nuclei in MyHC^+^ myotubes and myocytes).

### 2.4 Inhibitor Studies

C2C12 myoblasts in six‐well plates were pre‐treated for 30 min with compound C (20 μM; Abcam), wortmannin (1 μM; Sigma‐Aldrich) or SB203580 (10 μM; Sigma‐Aldrich) and then incubated in SS/SS containing Med/gAd or DM for 3 days. For myotube atrophy, differentiated myotubes were pretreated with the inhibitors as above, followed by incubation in SS containing Dex (10 μM) with or without Med ± inhibitors for 48 h.

### 2.5 C2C12 Myotube Atrophy Models

C2C12 myotube atrophy models have been described previously [4, 12]. Differentiated myotubes were pretreated with V/Med/gAd/Adiporon for 24 h and then with Dex (10 μM) or LT (1 μg/mL lipopolysaccharide (LPS; Invivogen) + 20 ng/mL TNF‐α [Biovision]) ± test compounds for 48 h. For PBS‐induced atrophy, myotubes were pretreated as above and incubated in Ca^2+^‐free or Ca^2+^‐containing PBS along with test compounds for 6 h. Cells were analysed by immunoblotting or microscopy. Images were captured using a Nikon ECLIPSE Ts2 microscope (Dex and LT). For the PBS model, an EVOS FL Auto Imaging System (Life Technologies) was used. For morphometry, 10 linear unbranched myotubes with the largest diameter/field were selected (180 myotubes/treatment group) and analysed with the freehand selection tool in the NIH ImageJ software as before [S14].

### 2.6 Fatty Acid Oxidation Assay

C2C12 cells (80 000 cells/well) differentiated for 4 days in 24‐well XF cell plates (Agilent) were pretreated with Med/gAd for 24 h, followed by Dex (10 μM) ± Med/gAd for further 48 h. Cells were then serum starved for 6 h and kept in Krebs–Henseleit buffer supplemented with glutamine (1 mM; Thermo Fisher Scientific) and L‐carnitine (0.5 mM; Cayman) for 45 min at 37°C in a non‐CO_2_ incubator. The first injection port of the XFe cartridge was loaded with 1.5 μM of FCCP (Abcam), and rest were left empty. Before loading the plates into the machine, 250 μM palmitate (Sigma‐Aldrich) or 38 μM BSA (MP Biomedicals) was added. The oxygen consumption rate (OCR) and extracellular acidification rate (ECAR) were determined using a Seahorse XFe24 Analyser following a measurement cycle of 3 min wait, 2 min mix and 3 min measurement. Values were normalized to protein concentration and were expressed as pmol/min/μg protein.

### 2.7 Animal Studies

All procedures were approved by the Institutional Animal Ethics Committee (IAEC) of CSIR‐CDRI; Approval No. IAEC/2022/44. Outbred Sprague–Dawley (SD) rats obtained from the National Laboratory Animal Center, CSIR‐CDRI, were maintained at 24°C ± 2°C, humidity: 50%–60%, light: 300 Lux in 12‐h light/dark cycles (3 animals/cage) and had access to a standard chow diet (Altromin, Lage, Germany) and water ad libitum. Animals were acclimatized for 7 days before initiation and randomized into groups based on body weight (bw). Euthanization was done by thrice the anaesthetic dose of ketamine‐xylazine (anaesthetic dose: ketamine [80 mg/kg], xylazine [10 mg/kg]).

#### 2.7.1 Denervation‐Induced Skeletal Muscle Atrophy

Male SD rats (8 weeks old; bw 225 ± 25 g) were randomly divided into two groups: *n* = 6/group. Animals were anaesthetized, the sciatic nerve on the left leg was exposed, a 5 mm section was excised, and the nerve ends were ligated. The right leg served as the sham control. Twenty‐four hours post surgery, the rats were given oral gavage of either vehicle (Den + V [1% gum acacia in distilled water] or Med [Den + Med in gum acacia: 5 mg/kg.bw/day] for 3 or 7 days). Gastrocnemius muscles (GN) were divided into two portions: One part was snap frozen, whereas the other was preserved in 10% NBF.

##### 2.7.1.1 Toe‐Spread Analysis

Toe**‐**spread analysis was done as before [4]. The paws were coated with a non‐toxic dye, and animals were gently positioned upright on a sheet of filter paper to allow both hind paws to make simultaneous contact with it. Scoring: 0 = *no spreading of the toes*, 1 = *intermediate toe spread*, 2 = *complete toe spread*.

#### 2.7.2 Dex‐Induced Skeletal Muscle Atrophy

Female SD (8 weeks, bw 255 ± 20 g) rats were trained on wire hanging apparatus, rotarod instrument and grip strength machine for 4 days and then were randomly divided into three groups (*n* = 6/group): control (vehicle‐treated; 1% gum acacia in distilled water; by oral gavage), Dex (200 μg/kg; i.p.) and Dex + Med (Dex (i.p.) + Med‐5 mg/kg.bw/day; oral gavage in 1% gum acacia). Treatments were administered for 15 days. BW and feed intake were recorded daily. Before sacrifice, blood was collected from the retro‐orbital plexus. Serum adiponectin was measured using an adiponectin ELISA kit (Thermo Fisher Scientific). GN muscles from the left hindlimb were snap‐frozen, whereas the right was preserved in 10% NBF.

##### 2.7.2.1 Functional Analyses

###### 2.7.2.1.1 Wire Hanging Test

Wire hanging test was done as before [4]. A 1‐mm‐diameter wire was horizontally positioned ~50 cm above the laboratory bench. The animals were allowed to grip it securely with their forepaws before being gently released. Muscle strength and posture were evaluated using a 6‐point scoring system: 0 = *falling off*, 1 = *hanging with two forelimbs*, 2 = *hanging with two forelimbs and attempting to climb on the wire*, 3 = *hanging on the wire with both forelimbs and hindlimbs*, 4 = *hanging on the wire with both forelimbs and hindlimbs and tail wrapped around the wire*, 5 = *attempting to escape after climbing on the wire with all four limbs and tail support*. Each animal underwent three trials, conducted in a blinded manner with 30‐min intervals between sessions.

###### 2.7.2.1.2 Grip Strength Analysis

Forelimb grip strength was assessed using a grip strength meter (Chatillon, DFE2‐010). Measurements were recorded in three sessions, 15 min apart, by an investigator blinded to the treatment groups.

###### 2.7.2.1.3 Rotarod Motor Coordination Analysis

Rats were trained on rotarod (IITC Inc./Life Science) for 4 days at 5–30 rpm for 5 min each. On the test day, the rotarod speed was fixed at 15 rpm (300 s/session). The latency to fall was recorded for each animal across three consecutive sessions, with a minimum 15‐min gap between sessions.

#### 2.7.3 Histology and Wheat Germ Agglutinin (WGA) Staining

The 7‐μm transverse sections were stained with haematoxylin and eosin (H&E) as before [4]. For wheat germ agglutinin (WGA) staining, sections permeabilized with 0.1% Triton X‐100 were incubated with Alexa Fluor 488–conjugated WGA (Thermo Fisher Scientific; 5 μg/mL in PBS) for 2 h; the nuclei were stained with DAPI and analysed by microscopy (H&E; Nikon Eclipse E200, WGA; Leica DMI6000B).

##### 2.7.3.1 Myofibre Quantification

Myofibre cross‐sectional area (CSA) and Feret's diameter were measured using ImageJ with the freehand selection tool. To avoid bias, all myofibres (> 1500/group) with distinct visible boundaries as determined by haematoxylin‐stained nuclei were analysed per field, excluding partial myofibres at the edges or ones with no visibly discernible boundaries (Dex study: *n* = 6 animals/group, 5 fields/animal, 30 images/group. Denervation study: *n* = 6 animals/group, 3 fields/animal, 18 images/group).

### 2.8 Statistical Analyses

Results are expressed as mean ± SEM; *n* = 3 or as indicated. One‐way or two‐way ANOVA followed by Bonferroni's post‐test or Kruskal–Wallis test, followed by Dunn's post‐test were used. Intra‐sample variances were calculated by Bartlett's test. Statistics involving two groups were performed by unpaired, two‐tailed, Student's *t*‐test (intra‐group variances were assessed by *F* test). GraphPad Prism 5 was used to analyse data; *p* < 0.05 was considered statistically significant.

## 3 Results

### 3.1 Medicarpin and Its Water‐Soluble Salt (Med) Are Dual AdipoR1/R2 Agonists

**FIGURE 1:**
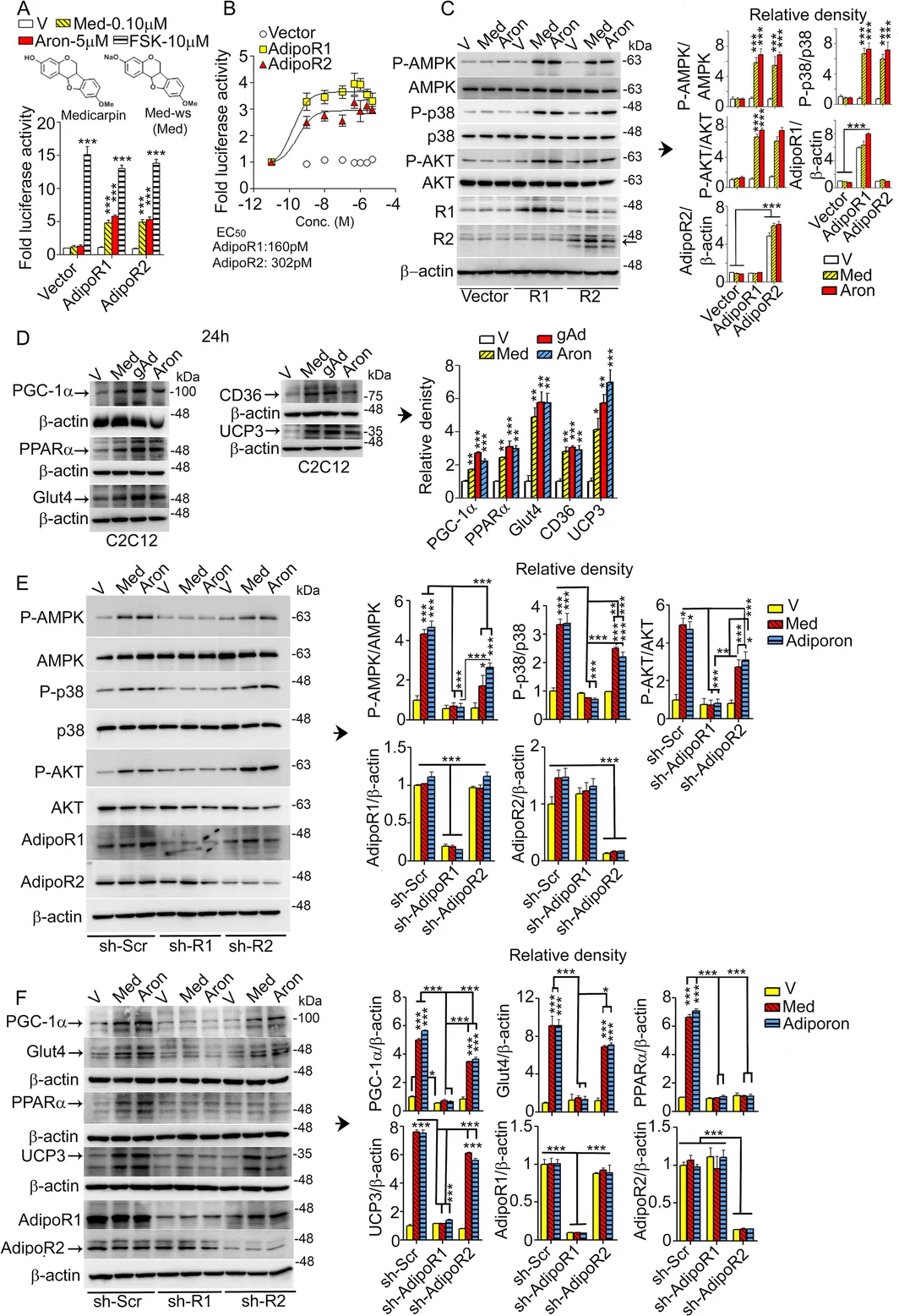
Med activates AdipoR1 and AdipoR2 and induces typical adiponectin‐associated signalling events. (A,B) Med induces PGC‐1α‐luc reporter in AdipoR but not empty vector‐transfected HEK‐293T cells. (A) HEK‐293T cells in 24‐well plates were transfected with (−1.1 kb) PGC‐1α‐luc (400 ng/well), and empty vector or AdipoR1/AdipoR2 expression plasmids (400 ng/well) along with EGFPN1 (normalizing control; 200 ng/well) were treated with vehicle (V) or test compounds (Med; 100 nM, Adiporon; 5 μM, Forskolin; 10 μM) for 24 h and luciferase activity was measured (*n* = 3 in triplicates), normalized with GFP and plotted. (B) HEK‐293T cells were transfected as above and were treated with seven concentrations of Med for 24 h, and luciferase/GFP values were plotted (*n* = 3 in triplicates). EC_50_ was calculated in GraphPad Prism 5. (C) Med induces adiponectin‐associated rapid signalling events in AdipoR‐transfected HEK‐293T cells. HEK‐293T cells in six‐well plates were transfected with AdipoR1/AdipoR2 or empty vector (2 μg/well) and EGFPN1 (500 ng/well) and 24 h after transfection were treated for 10 min with the indicated compounds, and the expression of indicated proteins was determined by immunoblotting. Adjoining graphs represent densitometry from three independent experiments. (D–F) Med treatment in differentiated C2C12 myotubes induces and activates factors associated with adiponectin signalling pathways, and AdipoR1/R2 knockdown compromises it. (D) C2C12 myotubes were treated with Med (100 nM), gAd (1 μg/mL) or Adiporon (5 μM) for 24 h and analysed by immunoblotting. Hereafter, Med, gAd and Adiporon conc used across all experiments were 100 nM, 1 μg/mL and 5 μM, respectively, unless otherwise indicated. (E) C2C12 myotubes were infected with lentivirus containing sh‐Scr/AdipoR1/AdipoR2 shRNAs. Forty‐eight hours after infection, cells were treated with the indicated compounds for 10 min (E) or 24 h (F) and analysed by immunoblotting. Adjoining graphs represent densitometries. Data are expressed as mean ± SEM; *n* = 3. Phospho‐proteins were normalized with their respective total proteins; for rest, β‐actin was used as a normalization control. Total proteins corresponding to each phospho‐protein or β‐actin corresponding to each protein were from the same blot; stripped and reprobed. Statistical analyses were performed by one‐way (D) or two‐way ANOVA (A,C,E,F) followed by Bonferroni's post‐test. *V versus treatment groups, empty vector versus AdipoR1/R2 groups. *p < 0.05, **p < 0.01, ***p < 0.0001. Aron, Adiporon; gAd, globular adiponectin.

Multiple osteogenic compounds previously identified by us were found to modulate AdipoRs [13, 19, 21, 26, S10]. We thus screened an in‐house library of osteogenic compounds to evaluate their AdipoR‐modulating potential in a previously established PGC‐1α‐promoter‐driven luciferase assay [19] in HEK‐293T cells (low endogenous AdipoR) overexpressing AdipoR1 or AdipoR2 and identified medicarpin [27] and its water‐soluble sodium salt; Med as AdipoR agonists (Figure 1A and Figure S1A). Both compounds activated the reporter in AdipoR‐overexpressing cells at 100 nM, whereas Adiporon did so at its reported active concentration of 5 μM [15] (Figure 1A and Figure S1A). Forskolin, which induces PGC‐1α through cyclic AMP pathway [30] activated the reporter independent of AdipoR‐expression (Figure 1A). Dose–response analysis yielded EC_50_ values of 160 and 302 pM for AdipoR1 and AdipoR2, respectively, indicating dual agonism by Med (Figure 1B). As the oral Cmax of medicarpin is ~300 nM in SD rats [27], 100 nM Med was selected as a pharmacologically relevant concentration for subsequent in vitro studies.

Consistent with adiponectin signalling [12, 13, 29, 31], Med induced rapid AMPK, AKT and p38 phosphorylations in AdipoR‐transfected HEK‐293T and AdipoR‐abundant C2C12 myotubes, similar to gAd (1 μg/mL) and Adiporon (Figure 1C and Figure S2). Med also increased protein and mRNA levels of canonical AdipoR targets [13, 31], including PGC‐1α, PPARα, Glut4, CD36 and UCP3 (Figure 1D and Figures S1B and S3). AdipoR1 knockdown in C2C12 myotubes abrogated Med‐ or Adiporon‐induced rapid signalling and target protein expressions, whereas AdipoR2 depletion was less effective (Figure 1E: first set of shRNAs and Figure S4A: second set of shRNAs). PPARα induction by Med or Adiporon however, was similarly reduced by depletion of either AdipoR (Figure 1F and Figure S4B). These results establish Med as a potent dual AdipoR1/R2 agonist, with AdipoR1 being the dominant mediator of its actions in muscle cells.

### 3.2 Med Induces Differentiation of C2C12 Myoblasts

**FIGURE 2:**
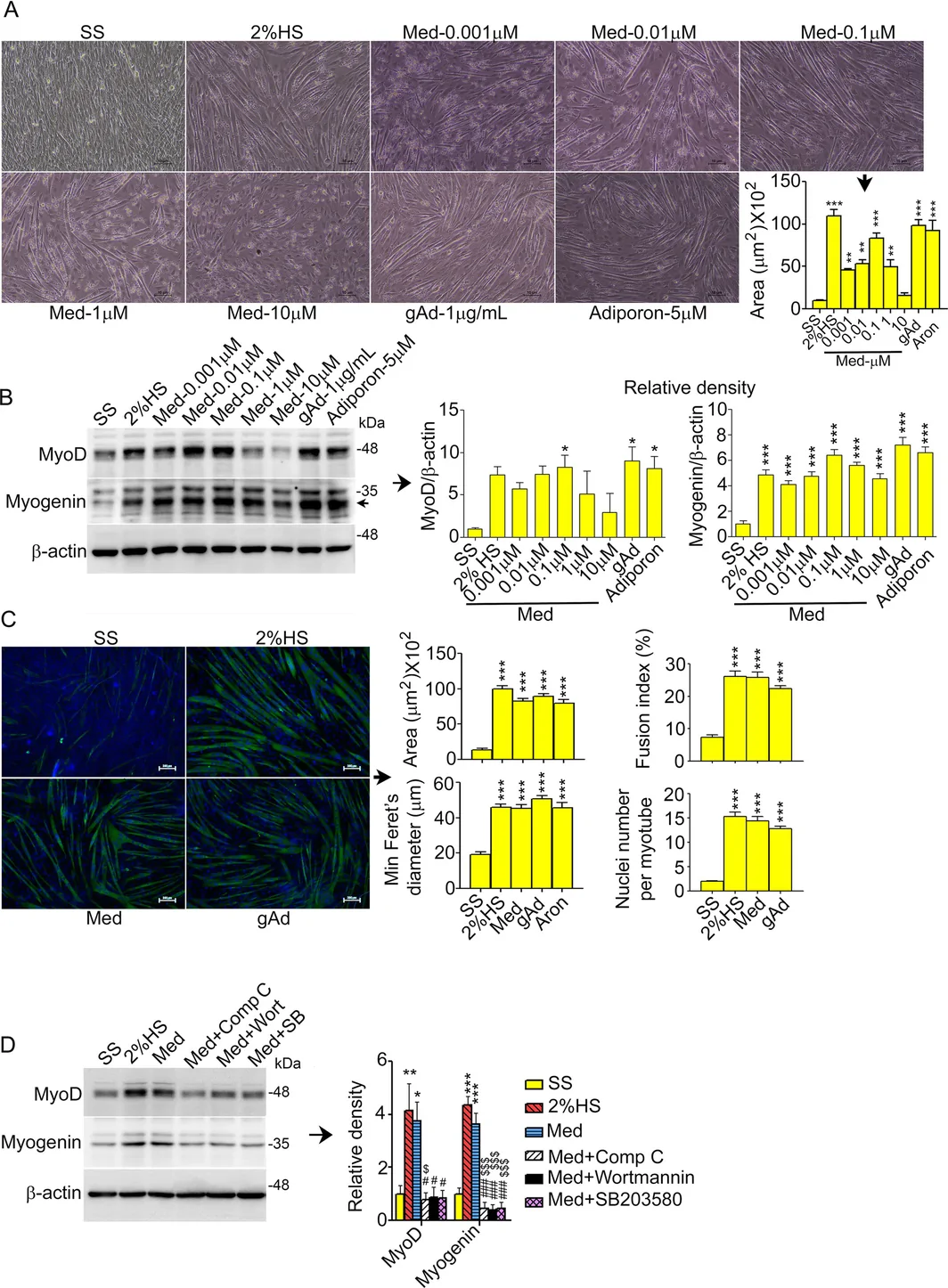
Med promotes myogenic differentiation of C2C12 myoblasts. (A) Representative phase‐contrast images showing morphological changes and myotube formation in 90% confluent C2C12 myoblasts differentiated for 3 days in serum‐free DMEM‐HG medium containing indicated concentrations of Med and compared with serum‐starved (SS), gAd or Adiporon‐treated cells (treatments were replenished every alternate day). 2% HS was used as a positive control. Representative phase‐contrast images are given (magnification: 100×; scale bar 10 μm). Graphs in the right panel represent calculated area (*n* = 3, 18 fields/treatment group, except for 1 and 10 nM Med‐treated cells, where 17 fields/treatment group were analysed). (B) Expression of myogenic markers was assessed by immunoblotting. C2C12 myoblasts were treated with increasing concentrations of Med. 2% HS, gAd and Adiporon were used as controls. Densitometry is given in the right panel. (C) Immunofluorescence staining for myosin heavy chain (MyHC) (MF‐20 antibody; green). Representative images are given (magnification: 100×, scale bar: 10 μm). Right panel: Quantification of myotube area, minimum Feret's diameter, nuclei number per myotube and fusion index (*n* = 3, 18 fields/group). (D) Evaluation of med‐induced myoblast differentiation in the presence of various signalling inhibitors. 90% confluent C2C12 myoblasts were pretreated with inhibitors: compound C (20 μM), wortmannin (1 μM), or SB203580 (10 μM) for 30 min, followed by treatment with Med or gAd for 3 days in serum‐free medium, and expression of MyoD and myogenin was determined by immunoblotting. All treatments were replenished every 24 h. Right panel shows densitometric quantification. Microscopy was performed by a Nikon ECLIPSE Ts2 microscope. For immunoblots, corresponding β‐actin was reprobed on the same blots. Graphs represent mean ± SEM (*n* = 3). *SS versus treatment groups, #HS versus treatment groups, $Med versus Med + inhibitors (A–D) Statistical analyses were performed by one‐way ANOVA followed by Bonferroni's post‐test. *,#,$*p* < 0.05, **,##,$$*p* < 0.01, ***,###,$$$*p* < 0.0001. Comp C, dorsomorphin; Wort, wortmannin; SB, SB 203580. Control groups pertaining to 2C (SS, 2% HS, gAd) are common with another study [S23].

Because adiponectin plays crucial roles in skeletal muscle including myogenesis [13, 24, 29, S15, S16], we evaluated the ability of Med to induce myoblast differentiation. Treating serum‐starved C2C12 myoblasts with Med robustly promoted myotube formation in a concentration‐dependent manner, with maximal effects at 100 nM (Figure 2A). This was accompanied by induction of MyoD, myogenin and myosin heavy chain (MyHC) (Figure 2B,C and Figure S5), along with higher nuclei count per myotube and fusion index (Figure 2C). Pharmacological inhibitors of AMPK (compound C), p38 MAPK (SB203580) and AKT (wortmannin) significantly reduced Med‐induced myogenic marker expression and myotube formation (Figure 2D).

### 3.3 Med Protects Against Myotube Atrophy Caused by Various Assaults

Corticosteroids such as Dex, inflammatory cytokines and nutrient‐deprivation induce atrophy in myotubes [4, 12, 32]. We thus examined whether Med could protect against atrophy induced by these stressors.

**FIGURE 3:**
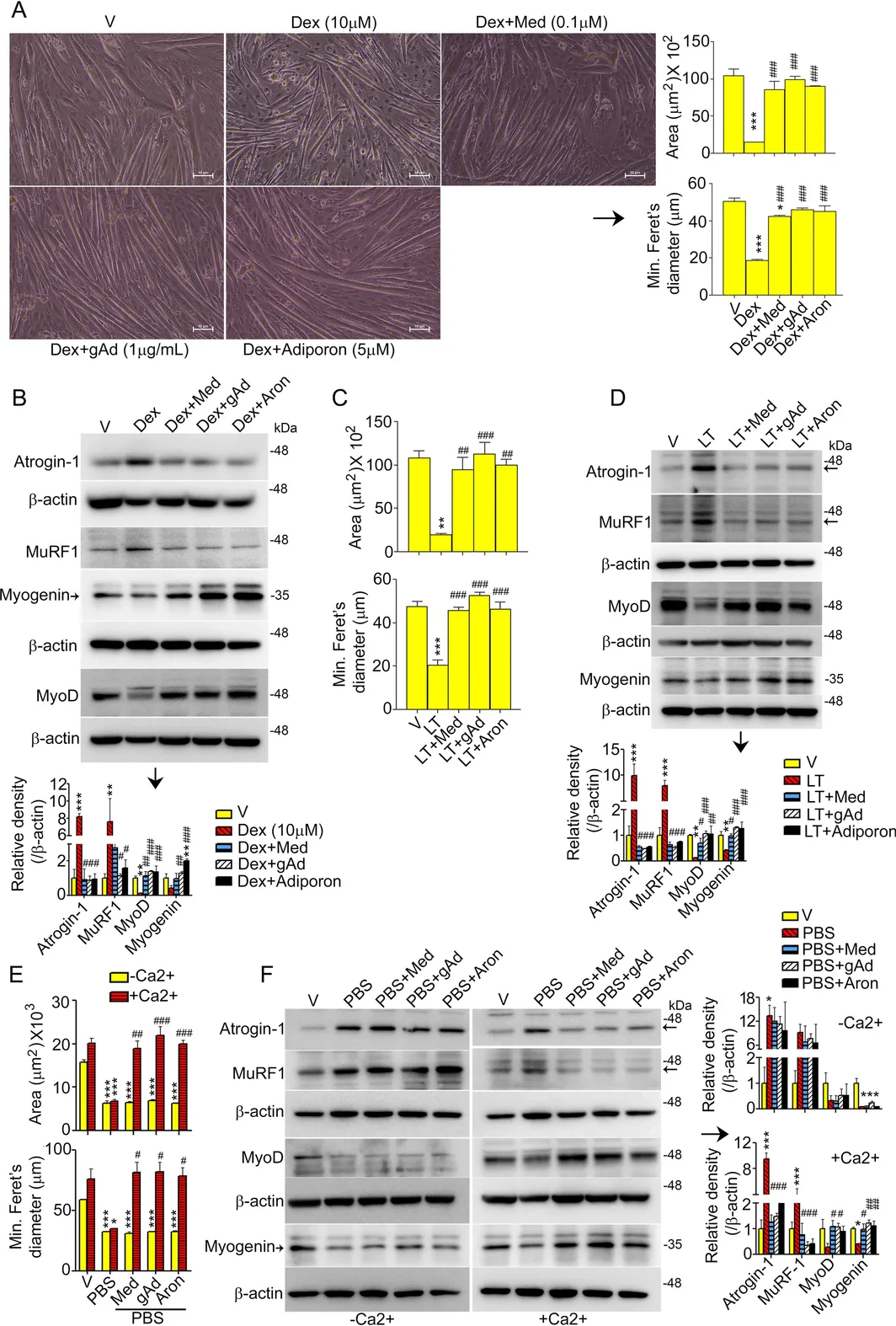
Med mitigates corticosteroid (Dex), cytokine (LPS + TNFα; LT) and nutrient deficiency (PBS)–induced myotube atrophy. (A,B) Med alleviates Dex‐induced myotube atrophy. Differentiated C2C12 myotubes were pretreated with Med, gAd or Adiporon for 24 h and were then treated with Dex (10 μM) ± Med, gAd or Adiporon for a further 48 h. Treatments were replenished every 24 h. Left panel: Representative phase‐contrast images. Right panel: Quantification of area and min. Feret's diameter (18 fields/treatment group). (B) Immunoblot analysis of indicated proteins. The lower panel shows densitometric analysis of three independent blots. (C,D) Med reduces LT‐induced myotube atrophy. Differentiated C2C12 myotubes were first pre‐treated with Med, gAd or Adiporon for 24 h, followed by LT (1 μg/mL lipopolysaccharide (LPS) + 20 ng/mL tumour necrosis factor alpha (TNF‐α)) followed by treatment with LT alone or plus test compounds for 48 h. (C) Myotube area and min. Feret's diameter, derived from microscopic images of the myotubes (corresponding representative images in Figure S7A). (D) Expression of indicated proteins was assessed by western blotting. Graphs in the lower panel are densitometric analysis; *n* = 3. (E,F) Med attenuated nutrient deficiency–induced myotube atrophy in the presence of Ca^2+^. Differentiated C2C12 myotubes were pre‐treated for 24 h with Med, gAd or Adiporon. The medium was then withdrawn and incubated in Ca^2+^‐free or Ca^2+^‐containing PBS for 6 h, along with Med, gAd or Adiporon. (E) Quantification of area and min. Feret's diameter (corresponding representative images in Figure S7B,C). (F) Immunoblot analysis of indicated proteins. Right panel: Densitometric analysis; *n* = 3. For Dex and LT experiments, images were captured using a Nikon ECLIPSE Ts2 microscope (magnification: 100×, scale bar: 10 μm). Eighteen fields/treatment group were analysed for morphometry. For the PBS‐induced atrophy model, myotubes were visualized using an EVOS FL Auto Imaging System (Life Technologies) (magnification: 100×, scale bar: 400 μm). For morphometry, 18 fields/treatment group were analysed. Data are presented as mean ± SEM (*n* = 3). *,#*p* < 0.05, **,##*p* < 0.01, ***,###*p* < 0.0001. *V versus treatment groups, #Dex/LT/PBS versus stressors + Med/gAd/Adiporon. Statistical analyses were performed by one‐way ANOVA followed by Bonferroni's post‐test.

Differentiated myotubes treated with Dex or LT displayed significantly reduced CSA and Feret's diameter, increased Atrogin‐1 and MuRF1 and suppressed MyoD and myogenin expressions, whereas Med/gAd/Adiporon attenuated these effects (Figure 3A–D and Figures S6 and S7A). PBS, a surrogate for nutrient deficiency, also induced similar effects as Dex and LT irrespective of whether it was supplemented with Ca2^+^, and Med or gAd/Adiporon could only ameliorate these effects in presence of Ca2^+^ (Figure 3E,F and Figure S7B,C), consistent with the requirement of Ca2^+^‐dependent signalling for adiponectin actions [12, 31]. These results demonstrate that Med protects against myotube atrophy via AdipoR‐dependent mechanisms.

### 3.4 Med Enhances Oxidative Fibre Markers and Fatty Acid Oxidation in C2C12 Myotubes

**FIGURE 4:**
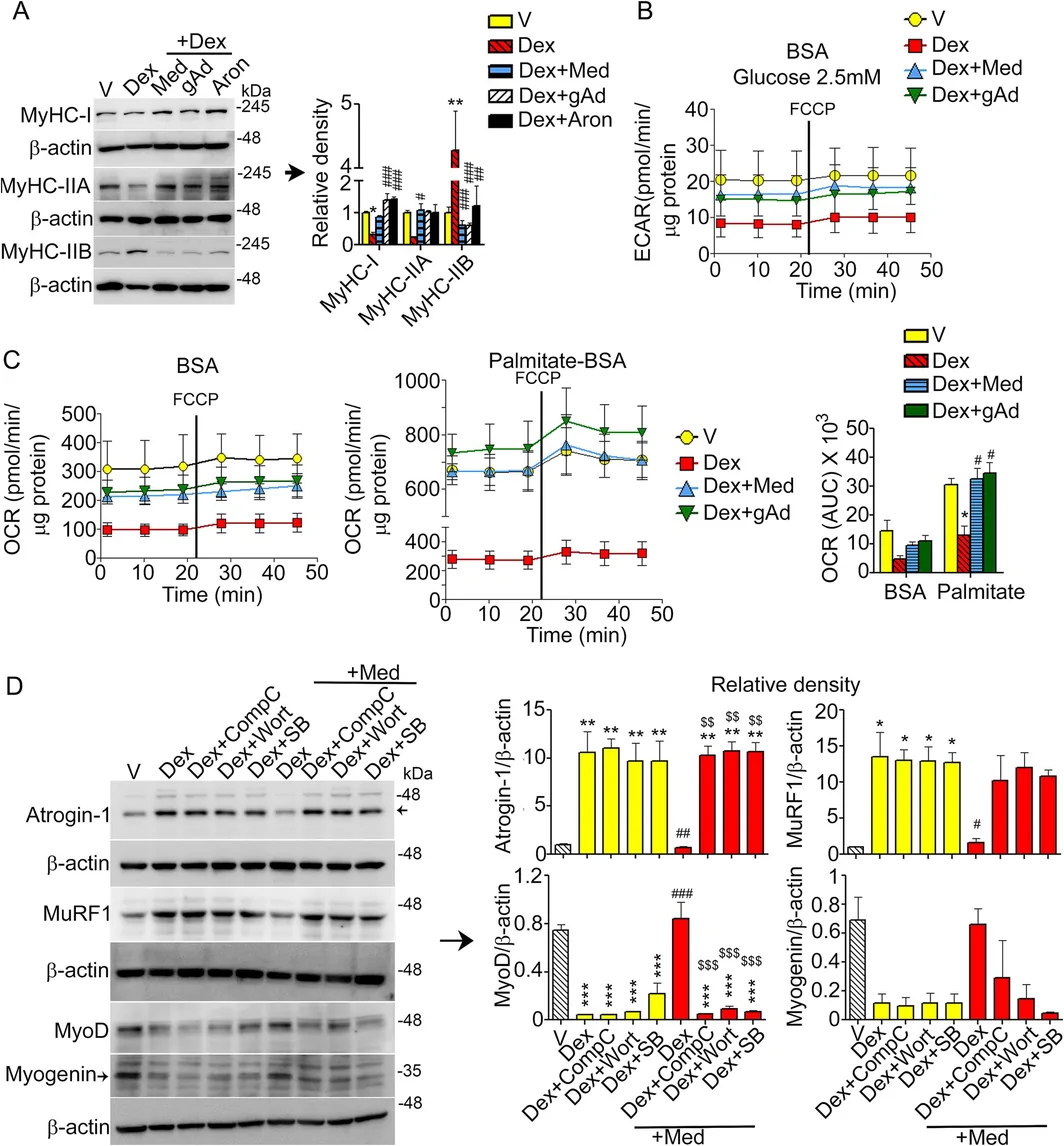
Med enhances oxidative metabolism and attenuates Dex‐induced changes in fibre‐type markers in C2C12 myotubes. (A) Dex increases glycolytic MyHC‐IIB and reduces oxidative MyHC‐I and IIA expression in C2C12 myotubes, and Med reverses it. Differentiated C2C12 myotubes were pretreated with Med, gAd or Adiporon for 24 h and were then treated with Dex for an additional 48 h. (A) Western blot (left) and quantification (right) of MyHC isoforms. (B,C) Med improves Dex‐mediated lowering of glycolytic and FAO rate in C2C12 myotubes. C2C12 myoblasts were plated on 24‐well Seahorse XFe cell culture plates (80 000 cells/well) and differentiated in the presence of 2% HS. Differentiated myotubes were treated as indicated for 48 h, following which ECAR was measured in the presence of 2.5 mM glucose (B), and OCR was measured using real‐time treatment with 250 μM palmitate (right panel) or 38 μM BSA (vehicle; left panel) (*n* = 3, in triplicates). (D) Inhibition of AMPK, AKT and p38 compromises Med‐mediated amelioration of Dex‐induced atrogene expressions. C2C12 myotubes were treated with compound C, wortmannin or SB203580 for 30 min, followed by treatment with Dex alone or Dex + Med for 48 h. Expressions of indicated protein levels were analysed by immunoblotting. All images are representative of three independent experiments. Graphs (mean ± SEM) corresponding to western blots represent densitometric analyses: *n* = 3. *,#,$*p* < 0.05, **,##,$$*p* < 0.01, ***,###,$$$*p* < 0.0001. *V versus treatment groups, #Dex versus treatment groups. $ Dex + Med versus Dex + Med + inhibitor groups. Statistical analyses were performed by one‐way ANOVA followed by Bonferroni's post‐test. ECAR, extracellular acidification rate; FCCP, carbonyl cyanide‐p‐trifluoromethoxyphenylhydrazone; MyHC, myosin heavy chain; OCR, oxygen consumption rate. Control groups (V, Dex, Dex + gAd) pertaining to (B,C) are common with another study [S23].

Adiponectin induces PGC‐1α, the master regulator of mitochondrial biogenesis and function [12, 13, 31], which influences muscle fibre type [33, S17]. As oxidative fibres are relatively resistant to fasting‐ or corticosteroid‐induced atrophy [34, S18], we investigated fibre‐type alterations in Dex‐induced myotube atrophy and their modulation by Med. Intriguingly, Dex reduced expression of the slow oxidative marker MyHC‐I and, to a lesser extent, MyHC‐IIA while significantly increasing the fast glycolytic marker MyHC‐IIB (Figure 4A). Med, gAd or Adiporon mitigated these Dex‐induced changes (albeit, Med reversal of MyHC‐I and gAd/Adiporon reversal of MyHC‐IIA were not statistically significant) (Figure 4A). Despite elevated MyHC‐IIB, Dex‐treated myotubes exhibited reduced glycolysis, indicated by lower ECAR, which was improved by Med or gAd (Figure 4B). Dex also decreased OCR, consistent with reduced oxidative markers, whereas Med and gAd restored OCR, particularly when palmitate served as the energy source (Figure 4C).

Pharmacological inhibition studies revealed that like myoblast differentiation (Figure 2D), the anti‐atrophy effects of Med required AMPK, p38 and AKT signalling (Figure 4D). These findings indicate that Dex promotes a shift towards a metabolically impaired fast glycolytic phenotype, whereas Med and gAd, through canonical adiponectin signalling, restore both glycolytic and oxidative capacities of affected myotubes.

To determine whether Med acted through direct glucocorticoid receptor (GR) antagonism, a GRE‐luciferase assay was performed. Neither Med nor gAd/Adiporon significantly suppressed Dex‐induced reporter activation in GR‐transfected HEK‐293T cells (Figure S8).

### 3.5 Med Improves Dex‐Induced Skeletal Muscle Atrophy in SD Rats

We next evaluated the in vivo effects of Med in a Dex‐induced systemic model of skeletal muscle atrophy. Eight‐week‐old female SD rats were treated for 15 days with V, Dex (200 μg/kg, i.p.) [22, 29] or Dex + Med (Dex, i.p.; Med, 5 mg/kg.bw daily by oral gavage). Dex caused marked body weight loss without affecting feed intake, and Med did not reverse this effect (Figure S9A,B). EchoMRI revealed reduced lean, but not fat mass, in Dex‐treated rats, which was also unaffected by Med (Figure S9C). Tibialis anterior (TA) and soleus muscle weights were unchanged among the groups (Figure S9D).

**FIGURE 5:**
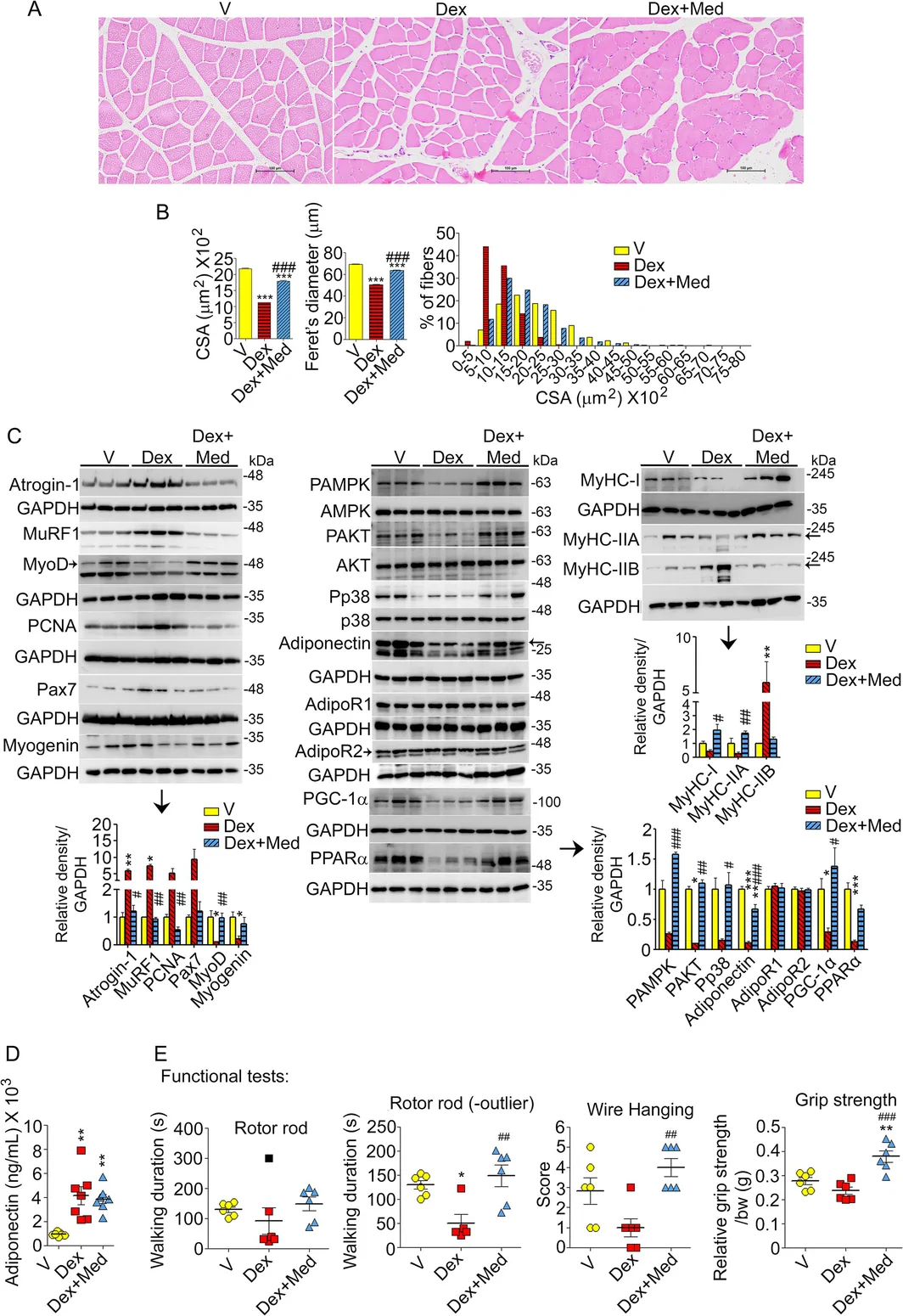
Med attenuates Dex‐induced skeletal muscle atrophy in vivo. Female SD rats were divided into three groups (V, Dex or Dex + Med) as described in the methods section. (A) Med improves Dex‐mediated structural alterations in GN muscle. Representative H&E staining of myofibre cross‐sections of GN muscle sections was captured using a Nikon Eclipse E200 microscope (magnification: 200×, Scale bar: 100 μm). (B) Quantitative analysis of myofibre cross‐sectional area (CSA). Feret's diameter, frequency distribution of CSA of total muscle fibre population obtained from GN muscle (control: 30 fields, Dex: 30 fields, Dex + Med: 29 fields, > 1500 myofibres/group) are shown. (C) Med reverses Dex‐mediated induction of atrophy‐related factors, suppression of adiponectin‐associated signalling molecules and fibre‐type alterations and enhances expression of myogenic markers. Expressions of indicated proteins in GN muscles were assessed by western blotting. GAPDH served as the loading control. Each lane represents a protein sample from an individual animal. Data from three animals are shown here, and the other three are given in the supplementary full western blots. GAPDH corresponding to each protein is from the same blot; stripped and reprobed. Corresponding graphs; *n* = 6/group. (D) Analysis of plasma adiponectin levels (*n* = 6/group). (E) Med restores Dex‐mediated reduction in functional parameters in rats. Functional evaluation of muscle performance by rotor rod, wire hanging and grip strength (*n* = 6). All images and immunoblots were quantified using ImageJ. Data are presented as mean ± SEM (*n* = 6/group). *,#*p* < 0.05, **,##*p* < 0.01, ***, ###*p* < 0.0001. *Control versus other groups, #Dex versus Dex + Med. Statistical analyses were performed by one‐way ANOVA followed by Bonferroni's post‐test (C: myogenin, adiponectin, AdipoR1, AdipoR2, MyHC‐IIA, E: all experiments except for the rotarod analysis with outlier) or Kruskal–Wallis test followed by Dunn's post‐test (all proteins in C, except those indicated above. E: rotarod analysis with outlier). Control groups pertaining to this experiment (V and Dex) are common with another study [S23].

However, H&E‐stained GN muscle sections revealed reduced Feret's diameter and increased numbers of small fibres (5–10 μm^2^) in the Dex group, both of which were significantly improved by Med (Figure 5A,B). Dex also increased Atrogin‐1 and MuRF1 expression, whereas levels in the Dex + Med group were comparable to V (Figure 5C). Skeletal muscle repair involves proliferation of the self‐renewing Pax7‐positive myosatellite cells followed by their recruitment in the myogenic lineage and early and late differentiation characterized by MyoD and myogenin expressions, respectively [4, 35]. Dex‐treated muscles displayed higher proliferating cell nuclear antigen (PCNA), and Pax7, but low MyoD and myogenin expressions (Figure 5C), indicating lower recruitment and differentiation of satellite cells. Dex + Med group, on the contrary, revealed PCNA, Pax7, MyoD and myogenin levels comparable with V (Figure 5C). The qRT‐PCR confirmed these findings, showing reduced Atrogin‐1 and MuRF1 and enhanced myogenin expression in the Dex + Med group (Figure S9E). These results suggest that Med either protects against Dex‐induced damage, promotes satellite cell differentiation or both.

Analysis of adiponectin signalling revealed that Dex markedly reduced P‐AMPK, P‐AKT, P‐p38, PGC‐1α and PPARα, whereas Med restored their expression to near‐control levels (Figure 5C). Dex also decreased skeletal muscle adiponectin, which was fully restored by Med, whereas AdipoR expression remained unchanged (Figure 5C). In contrast, serum adiponectin was elevated by Dex and unaffected by Med (Figure 5D), suggesting a greater role for local than systemic adiponectin in this model.

Consistent with the in vitro observations (Figure 4A), Dex reduced oxidative MyHC‐I and MyHC‐IIA markers and increased MyHC‐IIB. The Dex + Med group, in contrast, showed a similar pattern to the vehicle group (Figure 5C).

Functional testing demonstrated impaired grip strength and wire hanging performance in Dex‐treated rats, whereas the Dex + Med group performed significantly better, in some cases exceeding vehicle controls (Figure 5E). Rotarod performance also improved after exclusion of a highly active outlier from the Dex group (Figure 5E).

Together, these results demonstrate that Med mitigates Dex‐mediated skeletal muscle deterioration accompanied by reduced atrogene expression, restoration of adiponectin signalling, enhanced myogenic markers and increased oxidative fibre marker expression in GN muscle.

### 3.6 Med Mitigates Skeletal Muscle Atrophy Induced by Sciatic Nerve Denervation

**FIGURE 6:**
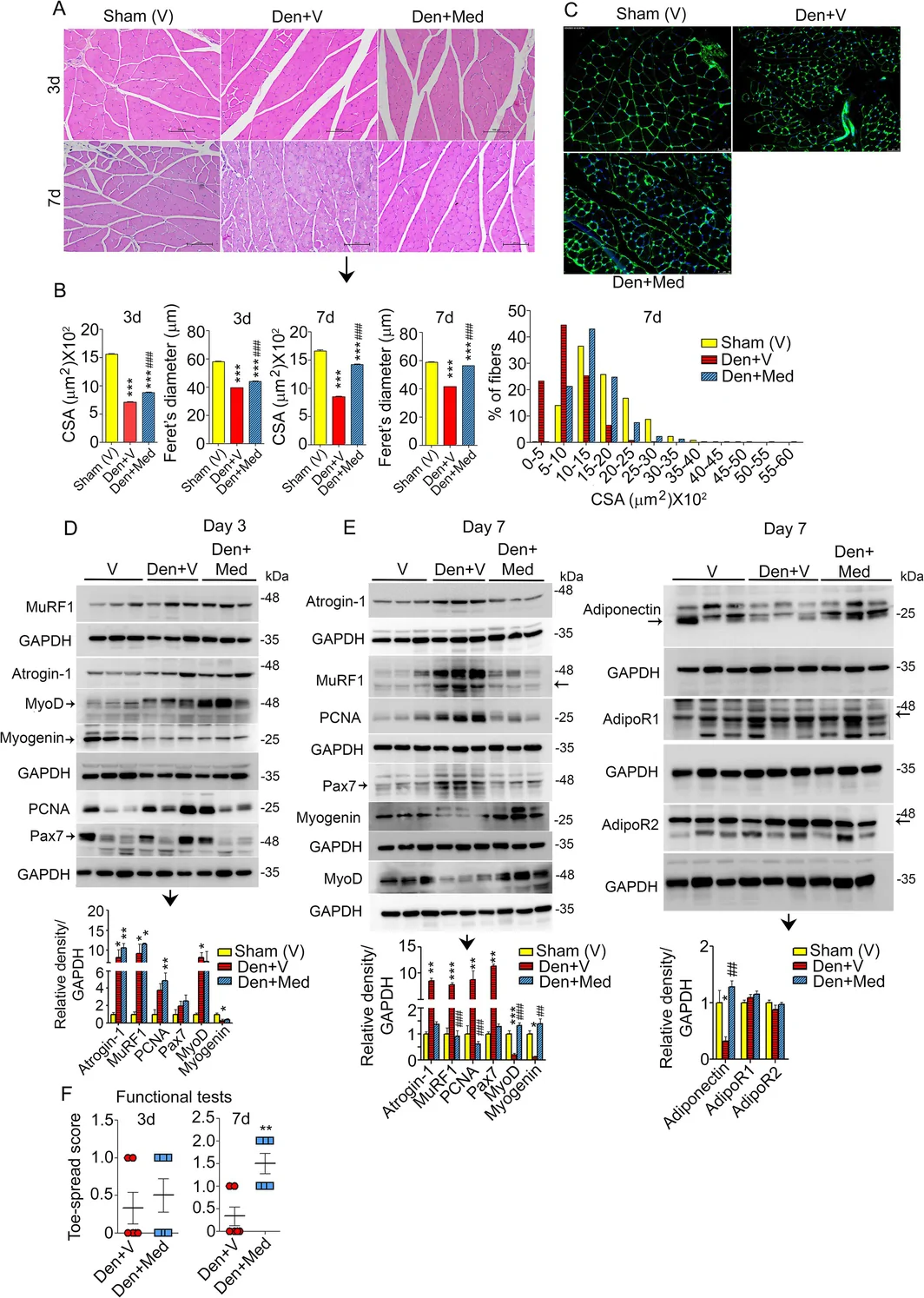
Med ameliorates sciatic nerve denervation‐induced skeletal muscle atrophy in SD rats. Male SD rats were randomized into two groups: control (vehicle‐treated, *n* = 6) and Med‐treated (*n* = 6). Sciatic nerve transaction and treatments were performed as described in the methods section. (A–C) Med partially mitigates denervation‐induced reduction of myofibre size. (A) Representative images of H&E‐stained cross‐sections of GN muscles from vehicle or Med‐treated animals at Day 3 or Day 7 post‐surgery were captured using a Nikon Eclipse E200 microscope (magnification: 200×, scale bar: 100 μm). (B) Bar graphs corresponding to H&E sections (A); quantification of cross‐sectional area (CSA) and Feret's diameter and frequency distribution of CSA of the total muscle fibre population in GN muscle (three fields/animal, total 18 fields/group, > 1500 myofibres/group). (C) Representative immunofluorescence images from 7 days showing WGA‐stained GN muscle sections outlining fibre boundaries (green) were captured using a Leica DMI6000B fluorescence microscope (magnification: 200×; scale bar: 50 μm). (D,E) Med ameliorates Den‐induced molecular alterations in GN muscles. Indicated protein expressions were analysed by immunoblotting in GN muscles from Day 3 (D) or Day 7 (E) of denervation. Each lane represents a protein sample from an individual animal. Data from three animals are shown here, and the other three are given in supplementary full western blots. GAPDH corresponding to each protein is shown from the same blot stripped and reprobed. Corresponding graphs represent data from six animals/group. (F) Med improves Den‐mediated functional impairment in affected limbs. Functional recovery was evaluated by the toe‐spread analysis (*n* = 6/group), corresponding paw prints with individual scores are in Figure S11. All quantification was performed using ImageJ. Data represent mean ± SEM *,#*p* < 0.05, **,##*p* < 0.01, ***,###*p* < 0.0001. Statistical analyses were performed by one‐way ANOVA followed by Bonferroni's post‐test (D: Pax7, myogenin. E: MuRF1, MyoD, Adiponectin, AdipoR1, AdipoR2) or Kruskal–Wallis test followed by Dunn's post‐test (B: all parameters. D: Atrogin‐1, MuRF1, PCNA, MyoD. E: Atrogin‐1, PCNA, Pax7, myogenin, F: Atrogin‐1, PCNA, Pax7, myogenin) and unpaired Student's *t*‐test (F). *Control versus other groups, #Den versus Den + Med. Den, denervation; WGA, wheat germ agglutinin.

Aside from its direct effect on the skeletal muscle, Dex also induces a whole‐body catabolic flux; thus, the possibility of Med amelioration of muscle atrophy via correction of Dex‐affected non‐muscle factors remains. To thus determine whether Med could ameliorate locally induced atrophy, we employed a sciatic nerve denervation model characterized by lower‐limb muscle wasting [36]. Twenty‐four hours post surgery (left hindlimbs), rats received vehicle (Den + V) or Med (Den + Med) for 3 or 7 days. Sham‐operated contralateral limbs from vehicle‐treated rats served as controls (sham + V). H&E (3 and 7 days) and WGA (7 days) staining of GN muscles showed marked fibre shrinkage, irregular fibre boundaries and increased interstitial space in Den + V compared with sham + V (Figure 6A–C). Med significantly attenuated these changes (Figure 6A–C). Den + Med animals displayed increased CSA and Feret's diameter by Day 3, and by Day 7, these parameters were comparable to sham + V (Figure 6B). Similarly, the marked increase in small fibres (0–10 μm^2^) observed in Den + V was significantly normalized by Med on Day 7 (Figure 6B).

At Day 3, both Den + V and Den + Med groups exhibited elevated Atrogin‐1, MuRF1, PCNA, Pax7 and MyoD, with reduced myogenin expression relative to sham + V (Figure 6D). By Day 7, however, although Den + V muscles continued to show high Atrogin‐1, MuRF1, PCNA and Pax7 levels, the expression of these factors in Den + Med was comparable to sham + V (Figure 6E). MyoD and myogenin levels were significantly higher in Den + Med than Den + V and were comparable to sham + V (Figure 6E).

Consistent with Figure 5D, muscle adiponectin expression was lower in Den + V than sham + V and was significantly increased by Med, whereas AdipoR1 and AdipoR2 expression remained unchanged across groups (Figure 6E). In unchallenged sham‐operated limbs (Day 7), Med had no significant effect on atrogenes, adiponectin signalling components, myogenesis or fibre‐type markers (Figure S10).

To assess if these structural and molecular improvements led to functional improvement, we performed a previously described [4] toe‐spread test. Whereas the scores were comparable between Den + V and Den + Med groups on Day 3, the Den + Med group scored significantly better on Day 7 (Figure 6F and Figure S11), indicating functional improvement in the operated limbs.

Taken together, Med alleviated denervation‐induced atrophy on Day 7, which was accompanied by reduced atrogene expression, increased satellite cell differentiation and functional improvements in the affected limbs.

## 4 Discussion

Here, we identified medicarpin and its water‐soluble salt Med as dual AdipoR1/R2 agonists that activate both receptors at picomolar concentrations. Med induced canonical adiponectin signalling and downstream targets, promoted myoblast differentiation and ameliorated skeletal muscle atrophy induced by Dex, inflammatory cytokines and nutrient deprivation in vitro. These effects were reproduced in rat models of Dex‐ and sciatic nerve denervation‐induced muscle atrophy.

Medicarpin was originally characterized as an osteogenic compound acting through p38 MAPK‐dependent oestrogen receptor activation [27], with subsequent studies implicating Wnt signalling and anti‐inflammatory mechanisms [37, 38]. Because adiponectin activates p38 [23], stimulates Wnt/β‐catenin signalling [S19], and suppresses inflammation [23], our findings strongly support AdipoRs as proximal targets of medicarpin and Med. This was further validated by Med‐induced activation of canonical adiponectin signalling and downstream targets in C2C12 myotubes, which were compromised upon AdipoR depletion.

Interestingly, despite its equipotent efficacy on both AdipoRs, AdipoR1 knockdown completely blocked Med‐induced AMPK, p38 and AKT phosphorylation and expression of PGC‐1α, Glut4 and UCP3, whereas AdipoR2 depletion produced partial inhibition. In contrast, PPARα, which is considered the primary target for AdipoR2 [31], required both receptors. A similar dependence on AdipoR1 for PPARα induction was reported for the AdipoR2‐selective agonist isovitexin [19]. Together with evidence for AdipoR1/AdipoR2 heterodimerization [39], these findings suggest functional cooperation between the two receptors.

Consistent with reported myogenic effects of adiponectin or its mimics [12, 23, 40, S9, S11, S15, S20], Med induced myoblast differentiation and ameliorated myotube atrophy caused by various assaults, which was accompanied by preservation of myotube size, suppression of atrogenes and restoration of MyoD and myogenin expression. In nutrient deprivation–induced atrophy, Med's effects were Ca^2+^ dependent, reinforcing its reliance on AdipoR‐mediated signalling. Pharmacological inhibition of AMPK, p38 or AKT abolished Med's myogenic and anti‐atrophy effects, demonstrating that all three pathways are required.

Med also promoted a shift towards oxidative fibre types by increasing MyHC‐I and MyHC‐IIA and reversing Dex‐induced MyHC‐IIB expression. Although Dex primarily affects the glycolytic but not the oxidative muscle fibres [S21], Dex‐treated myotubes unexpectedly displayed a fast glycolytic phenotype characterized by reduced MyHC‐I/MyHC‐IIA and elevated MyHC‐IIB. Despite this shift, both glycolytic activity and oxidative metabolism were impaired in Dex‐treated myotubes, suggesting that Dex induces a metabolically compromised glycolytic state. Combined with glucocorticoid‐induced insulin resistance and lipolysis, this may contribute to muscle vulnerability during chronic glucocorticoid exposure.

In vivo, Med improved myofibre morphology and grip strength, rotarod and wire hang performance in Dex‐treated rats without affecting Dex‐induced body weight loss, consistent with findings for Adiporon [S15] and other agents [S22]. Although Dex increased Pax7 and PCNA expression, differentiation markers were suppressed. Med restored MyoD and myogenin, indicating enhanced satellite cell differentiation. Med also reversed suppression of oxidative fibre markers and adiponectin signalling components by Dex. Notably, Dex reduced muscle adiponectin despite elevated circulating levels, whereas Med restored local muscle adiponectin without altering serum concentrations, suggesting a dominant role for local adiponectin signalling in muscle maintenance. Importantly, Med, gAd and Adiporon did not inhibit Dex‐induced GR activity, indicating a potential therapeutic advantage for treatment of muscle atrophy in patients requiring corticosteroids for other indications.

Similar ameliorative effects were observed in the denervation model, where Med suppressed atrogenes, enhanced satellite cell differentiation markers and restored muscle adiponectin. In both models, Med improved muscle function in addition to preserving muscle morphology, distinguishing it from several experimental therapies that increase muscle mass without improving performance [6, 9].

Despite these findings, certain limitations in this study must be acknowledged: First, a Med‐only group was not included in unchallenged animals. Although sham‐operated limbs from the denervation study showed no differences in atrogenes, adiponectin signalling myogenic or fibre‐type markers between vehicle and Med groups, long‐term studies are needed. Second, the Dex study did not evaluate additional metabolic parameters or other organs affected by Dex and/or AdipoR agonists, which are currently under investigation. Finally, although Med displayed favourable pharmacological properties compared with other AdipoR agonists, including Adiporon, particularly regarding receptor activation, efficacy at sub‐micromolar concentrations, oral bioavailability (22%) and plasma half‐life (5.33 h) [27], further studies on safety pharmacology, toxicity and off‐target effects are required and are currently underway.

## 5 Conclusion

Our findings identify Med as a dual AdipoR1/R2 agonist that promotes myoblast differentiation and ameliorates skeletal muscle atrophy induced by diverse insults in vitro and in vivo. Med acts through activation of canonical adiponectin signalling, enhancement of oxidative metabolism and satellite cell differentiation. Our results also indicate that skeletal muscle atrophy may involve suppression of local adiponectin signalling. Collectively, these findings strengthen the rationale for targeting AdipoRs in skeletal muscle atrophy and establish Med as a promising scaffold for development of translatable AdipoR agonists.

## Funding

This work was supported by Council of Scientific and Industrial Research, India (MLP2028) and the Blockchain for Impact‐BIOME (GAP0485).

## Ethics Statement

All experimental procedures were approved by the Institutional Animal Ethics Committee (IAEC) of CSIR‐Central Drug Research Institute; Approval No. IAEC/2022/44.

## Conflicts of Interest

The authors declare no conflicts of interest.

## Supporting information


**Data S1:** Supporting Information.

**Table S1:** Antibody details.
**Table S2:** List of primers.
**Figure S1:** Medicarpin (DMSO soluble) activates AdipoR1 and AdipoR2 and induces adiponectin targets. (A) HEK‐293T cells transfected with PGC‐1α‐luc, empty vector or AdipoR1/AdipoR2 expression plasmids along with eGFPN1 were treated with vehicle (V), medicarpin or Adiporon at indicated concentrations for 24 h, and luciferase/GFP values were plotted as fold luciferase activity. (B) C2C12 myotubes were treated as indicated with Medicarpin (100 nM), Med (100 nM) or Adiporon (5 μM) for 24 h, and protein expression was analysed by immunoblotting. β‐Actin was reprobed on the same blot. Right panel graph represents densitometry (mean ± SEM; *n* = 3). *p < 0.05, **p < 0.01, ***p < 0.0001. Statistical analyses were performed by two‐way ANOVA (A) or one‐way ANOVA (B), followed by Bonferroni's post‐test.
**Figure S2:** Med treatment in differentiated C2C12 myotubes induces and activates factors associated with adiponectin signalling pathways. C2C12 myotubes were treated with Med (100 nM), gAd (1 μg/mL) or Adiporon (5 μM) for 10 min and analysed by immunoblotting. Graph represents mean ± SEM (*n* = 3). Statistical analyses were performed by one‐way ANOVA followed by Bonferroni's post‐test. *p < 0.05, **p < 0.01, *** < 0.0001. Aron, Adiporon; gAd, globular adiponectin.
**Figure S3:** Med treatment in differentiated C2C12 myotubes induces adiponectin target gene expressions. C2C12 myotubes were treated with Med (100 nM), gAd (1 μg/mL) or Adiporon (5 μM) for 24 h and analysed by qRT‐PCR. Graph represents mean ± SEM (*n* = 3). Statistical analyses were performed by one‐way ANOVA, followed by Bonferroni's post‐test (CD36), or Kruskal–Wallis test followed by Dunn's post‐test (PGC‐1α, Glut4, PPARα). *p < 0.05, **p < 0.01, ***p < 0.0001.
**Figure S4:** Med treatment in C2C12 myotubes induces and activates factors involved in adiponectin signalling pathways, and AdipoR1/R2 knockdown compromises it. C2C12 myotubes were infected with lentivirus (Set‐2) containing shscr/AdipoR1/AdipoR2 shRNAs. Forty‐eight hours after infection, cells were treated with the indicated compounds for 10 min (A) or 24 h (B). Phospho‐proteins were normalized with their respective total proteins (on same blot); for rest, β‐actin (on same blot) was used as normalization control. Graphs represents mean ± SEM (*n* = 3). *p < 0.05, **p < 0.01, ***p < 0.0001. Statistical analyses were performed by two‐way ANOVA (A,B), followed by Bonferroni's post‐test.
**Figure S5:** Med promotes myogenic differentiation of C2C12 myoblasts. C2C12 myoblasts were treated as indicated for 3 days and analysed by immunoblotting. Graph represents mean ± SEM (*n* = 3). Statistical analyses were performed by one‐way ANOVA followed by Bonferroni's post‐test. *p < 0.05, **p < 0.01, ***p < 0.0001.
**Figure S6:** Med reverses Dex induction of atrogenes and restores Dex‐mediated suppression of myogenin mRNA expression in C2C12 myotubes. Graph represents (mean ± SEM; *n* = 3). *V versus treatment groups, #Dex versus treatment groups. *p < 0.05, **p < 0.01, ***p < 0.0001. Statistical analyses were performed by Kruskal–Wallis test followed by Dunn's post‐test. Aron, Adiporon; gAd, globular adiponectin. Control groups (V, Dex, Dex + gAd, Dex + Adiporon) are common with another study [S23].
**Figure S7:** Med mitigates (LPS + TNFα; LT), and nutrient‐deficiency (PBS)–induced myotube shrinkage. Phase‐contrast microscopic images of LT‐induced atrophy model (A), PBS without Ca2 + (B) and PBS with Ca2 + (C). Images in A were captured using a Nikon ECLIPSE Ts2 microscope (magnification: 100×; scale bar: 10 μm, 18 fields/treatment group). Images in B and C were captured using EVOS FL Auto Imaging System (Life Technologies) (magnification: 100×, scale bar: 400 μm, 18 fields/treatment group). Morphometric analyses of (A–C) are provided in Figure 3.
**Figure S8:** Med does not suppress Dex‐mediated GR activation in a GRE‐Luciferase activity assay. HEK‐293T cells seeded in 48‐well plates were transfected with 200 ng/well of empty vector/GR expression plasmid, GRE‐luciferase reporter (200 ng/well) and EGFPN1 (100 ng/well). 24 h post‐transfection, cells were treated with V, Dex (10 μM) or Dex + test compounds for further 24 h. Data represent mean ± SEM. *p < 0.05, **p < 0.01, ***p < 0.0001. *Vehicle‐treated GR‐transfected versus GR‐transfected + test compounds. Statistical analysis was performed by two‐way ANOVA followed by Bonferroni's post‐test. gAd, globular adiponectin; Aron, Adiporon. Control groups (V, Dex, Dex + gAd, Dex + Adiporon) are common with another study [S23].
**Figure S9:** Evaluation of the effect of Dex and Med on feed intake, body composition, muscle weight and muscle mRNA expressions. Feed intake (A) body weight (B) of rats from indicated groups (*n* = 6/group). (C) Lean mass and Fat mass (in grams) of rats from indicated groups (*n* = 6/group) measured using an EchoMRI‐500 Body Composition Analyser (EchoMRI Corporation Pvt. Ltd., Singapore). (D) Absolute and relative muscle weights of TA and Soleus muscles from left hindlimbs (*n* = 6/group). E. Transcript levels of Atrogin‐1, MuRF1 and myogenin in GN muscle of rats from indicated treatment groups (*n* = 3 in triplicates). All graphs represent mean ± SEM. *V versus treatment groups, #Dex versus treatment groups. *#*p* < 0.05, **##*p* < 0.01, ***##*p* < 0.0001. Statistical analysis performed using one‐way ANOVA followed by Bonferroni's post‐test for (C–E). For Atrogin1 and MuRF1, except for myogenin in (E). Kruskal–Wallis test was performed followed by Dunn's post‐test. Control groups (V, Dex) are common with another study [S23].
**Figure S10:** Med treatment does not significantly alter expressions of the proteins examined in sham‐operated GN muscles. Immunoblot panels represent expression of indicated proteins in GN muscles from sham‐operated limbs of vehicle (*n* = 6) or Med (*n* = 6) ‐treated rats corresponding to the denervation experiment (main Figure 6). Graph represents densitometric analysis (mean ± SEM). MyHC‐I, MyHC‐IIA and MyHC‐IIB were normalized with α‐actinin, and the rest were normalized with GAPDH. Statistical analysis was performed by unpaired, two‐tailed Student's *t*‐test, except for MyoD and MyHC‐IIB, where Mann–Whitney test was used. F test was used to assess intra‐group variances.
**Figure S11:** Paw print images from Day 3 and Day 7, post‐denervation. Paw prints from individual rats are labelled along with their respective scores. Left hindlimbs in each case was denervated, and the right limb served as sham and was taken as benchmark (score: 2) for full opening of the paws. Corresponding graphs are in the main Figure 6F.

## Data Availability

The data that support the findings of this study are available from the corresponding author upon reasonable request.
