# Supplementary material for: A Novel Small‐Molecule Dual Adiponectin Receptor 1/2 Agonist Induces Myogenesis and Ameliorates Skeletal Muscle Atrophy
Source: J Cachexia Sarcopenia Muscle. 2026 Aug 24;17(4):e70364. doi: 10.1002/jcsm.70364 (PMC13502058; doi:10.1002/jcsm.70364)
Supplement: Supplementary file 1 [file JCSM-17-e70364-s001.pdf]

Supplementary Methods:

Luceferase reporter assay: HEK-293T cells in 24-well plates were transfected with a PGC-1α-luciferase reporter (400ng/well), empty vector/AdipoR1/R2 expression plasmids (400ng/well), and 200ng EGFPN1 (normalization control)/well (plasmids are described previously (Khatoon et al., 2024)) using Lipofectamine 3000 according to the manufacturer's protocol. 24h post-transfection, cells were treated as indicated for an additional 24h. GFP and luciferase activity were quantified using a SpectraMax M-series multimode plate reader and a GloMax luminometer (Promega), respectively. GRE-luciferase assays were performed similarly (detailed in **supplementary Figure S8**).

AdipoR overexpression and evaluation of rapid signaling events: HEK-293T cells in 6-well plates were transfected as above with 2µg/well empty vector/AdipoR1/AdipoR2 constructs, and EGFPN1 (500ng/well). 24h after transfection, cells were treated with indicated compounds for 10 min and were analyzed by immunoblotting.

Khatoon, S., Das, N., Chattopadhyay, S., Joharapurkar, A., Singh, A., Patel, V., Nirwan, A., Kumar, A., Mugale, M.N., Mishra, D.P., Kumaravelu, J., Guha, R., Jain, M.R., Chattopadhyay, N. and Sanyal, S., 2024. Apigenin-6-C-glucoside ameliorates MASLD in rodent models via selective agonism of adiponectin receptor 2, Eur J Pharmacol. 978, 176800.

shRNA-mediated knockdown in C2C12 myotubes: For lentiviral packaging of the shRNAs, HEK-293T cells in 150mm dishes were transfected with lentiviral packaging plasmid psPAX2 (7.5µg/dish; Addgene), lentiviral envelope plasmid pMD2.G (7.5µg/dish; Addgene), and plasmids bearing shRNA sequence for sh-scr (scramble)/sh-AdipoR1/sh-AdipoR2 (15µg/dish) using standard calcium phosphate method. Media was collected from the transfected plates at 48 and 72h, pooled and filtered with a 0.45µm syringe filter (Merck). Lentivirus particles were then precipitated using PEG8000 (Sigma-Aldrich), and the viral pellet was resuspended in chilled PBS and stored at −80°C until use. For knockdown, C2C12 myotubes in 6-well plates were infected with lentivirus containing sh-scr/AdipoR1/AdipoR2 along with polybrene (Sigma-Aldrich). After 48h, the transduction media were replaced, and the cells were treated as indicated. Following harvest, lysates were used for immunoblotting. Mouse shRNAs were from Sigma-Aldrich in pLKO.1 vector. AdipoR1; TRC Clone ID TRCN0000249149 (set-1); Target Sequence:GGGATTGCTCTACTGATTATG,

TRCN0000257836 (set-2); Target Sequence:AGATGGAGGAGTTCGTGTATA, AdipoR2; TRC Clone ID TRCN0000175771 (set-1); Target Sequence:GCAGGAATTTCGTTTCATGAT, TRCN0000173201, (set-2); Target Sequence: CCATCATGCTATGGAACGAAT.

Protein Extraction and Immunoblotting: Cell samples were harvested and homogenized in RIPA lysis buffer supplemented with protease and phosphatase inhibitor cocktails (Sigma-Aldrich). 40-70μg of total protein were resolved by SDS PAGE, on 8-15% gels depending on the molecular mass of the target proteins, transferred onto PVDF membranes (Merck) and sequentially probed with primary and HRP-conjugated mouse or rabbit secondary antibodies (Sigma-Aldrich) as appropriate (details in **supplementary Table S1**). Images were acquired using an ImageQuant LAS 4010 Chemidoc (GE Healthcare) using a chemiluminescence kit (Merck). Blots were stripped and reused whenever possible. Loading controls for each protein were acquired on the same blots after stripping and reprobing and are shown. For rat muscle, frozen GN muscles were pulverized in liquid nitrogen, to which RIPA lysis buffer (supplemented with protease and phosphatase inhibitors) was added, and then the samples were analyzed as above. Full blots from all independent sets of experiments are provided as Supplementary Western blots. Densitometric analyses were performed using the ImageJ software.

Quantitative real-time polymerase chain reaction (qRT-PCR): Total RNA was harvested from C2C12 myotubes or snap-frozen GN muscle tissue using a Direct-zol RNA MiniPrep kit (Zymo Research, CA, USA). Total RNA (1µg) was reverse transcribed using a NeoScript cDNA synthesis kit (Genes2Me, India). SYBR Green Master mix (Applied Biosystems, MA, USA), was used for quantitative determination of relative mRNA expression using a CFX96 Real-Time PCR System (Bio-Rad, CA, USA) and ΔΔCT method, with β-actin (for myotubes) or GAPDH (for muscle tissue) used as reference genes (myotubes: n=3, muscle tissue: n=3 rats/group, in triplicates). Primer sequences are provided in the **supplementary Table S2**.

EchoMRI: Body composition of live SD rats was analyzed using an EchoMRI-500 Body Composition Analyzer (EchoMRI Corporation Pvt. Ltd, Singapore) on day 0, 7, and 14. Prior to analysis, the machine was calibrated by scanning columns of canola oil and distilled water provided by the manufacturer.

Supplementary Table S1. Antibody details

Sl no.	Antibody	Catalog no.	Manufacturer	Dilution
1	Adiponectin (19F1)	MA1-054	Thermo Fisher Scientific	1/1000
2	AdipoR1	DF6430	Affinity Biosciences	1/1000
3	AdipoR2	DF12811	Affinity Biosciences	1/1000
4	Phospho-AMPKα (Thr172) (40H9)	#2535	Cell Signaling Technology	1/1000
5	AMPKα (D5A2)	#5831	Cell Signaling Technology	1/1000
6	Phospho-p38 MAPK (Thr180/Tyr182) (D3F9)	#4511	Cell Signaling Technology	1/1000
7	p38 MAPK (D13E1)	#8690	Cell Signaling Technology	1/1000
8	Phospho-Akt (Ser473) (193H12)	#4058	Cell Signaling Technology	1/1000
9	Akt (pan) (C67E7)	#4691	Cell Signaling Technology	1/1000
10	PGC-1α	ab54481	Abcam	1/1000
11	PPARα (3B6/PPAR)	MA1-822	Thermo Fisher Scientific	1/1000
12	Glut4 (1F8)	#2213	Cell Signaling Technology	1/1000
13	UCP3	ab3477	Abcam	1/1000
14	CD36	sc-7309	Santa Cruz	1/1000
15	MYOD	MA1-41017	Thermo Fisher Scientific	1/1000
17	Myogenin	ab124800	Abcam	1/1000
18	MURF1	ab172479	Abcam	1/1000
19	Fbx32	ab168372	Abcam	1/1000
20	PCNA (PC10)	#2586	Cell Signaling Technology	1/1000
21	Pax7 (EPR3353)	ab92317	Abcam	1/1000
22	MyHC(MF20)	DSHB, #MF20	DSHB	1/200
23	Myosin heavy chain Type I	DSHB, #BA-D5	DSHB	0.3µg/mL
24	Myosin heavy chain Type IIA	DSHB, #SC-71	DSHB	0.3µg/mL
25	Myosin heavy chain Type IIB	DSHB, #BF-F3	DSHB	0.3µg/mL
26	GAPDH (14C10)	#3683	Cell Signaling Technology	1/1000
27	Rat Adiponectin Rapid ELISA Kit	EELR028	Thermo Fisher Scientific	1/2
28	β-Actin	A3854	Sigma-Aldrich	1/35,000
29	alpha-Actinin	#6487	Cell Signaling Technology	1/1000
30	Anti-Rabbit IgG secondary	A9169	Sigma-Aldrich	1/10,000
31	Anti-Mouse IgG secondary	A9044	Sigma-Aldrich	1/10,000

Supplementary Table S2. List of primers

Rat	Forward Primer	Tm (°C)	Reverse Primer	Tm (°C)	Accession no.
Atrogin-1	GAAGACCGGCTACTGTGGAA	59.4	ATCAATCGCTTGCGGATCT	54.5	NM_133521.2
MuRF1	AGGACTCCTGCCGAGTGAC	61	TTGTGGCTCAGTTCCTCCTT	57.3	AY059627.1
Myogenin	CTACAGGCCTTGCTCAGCTC	57.9	TGGGAGTTGCATTCACTGG	55.3	NM_017115.3
GAPDH	TGGGAAGCTGGTCATCAAC	56.7	GCATCACCCCATTTGATGTT	55.3	NM_017008.4
**Mouse**	**Forward Primer**	**Tm (°C)**	**Reverse Primer**	**Tm (°C)**	**Accession no.**
Ppargc1a	ATGGCTTGGGACATGTGCAG	60.97	GCAAAGAGGCTGGTCCTCAC	60.96	NM_008904.3
Ppara	TCCGAGGGCTCTGTCAT	56.67	GGGCAGCTGACTGAGGA	58.15	NM_001113418.1
Cd36	TTGTACCTATACTGTGGCTAAATGAGA	59.66	CTTGTGTTTTGAACATTTCTGCTT	57.35	NM_001159555.2
Glut4	GACGGACACTCCATCTGTTG	58.29	GCCACGATGGAGACATAGC	58.09	NM_009204.3
Trim63	CCTGCAGAGTGACCAAGGA	58.94	GGCGTAGAGGGTGTCAAACT	59.68	NM_001369245.1
Fbxo32	GATCAAACGCTTGCGAATCT	57.19	AGTGAGGACCGGCTACTGTG	61.25	NM_026346.3
Myog	CTACAGGCCTTGCTCAGCTC	60.46	TGGGAGTTGCATTCACTGG	57.66	NM_031189.2
β actin	CCTCACCCTCCCAAAAGC	57.58	GTGGACTCAGGGCATGGA	58.60	NM_007393.5

Supplementary Figure S1

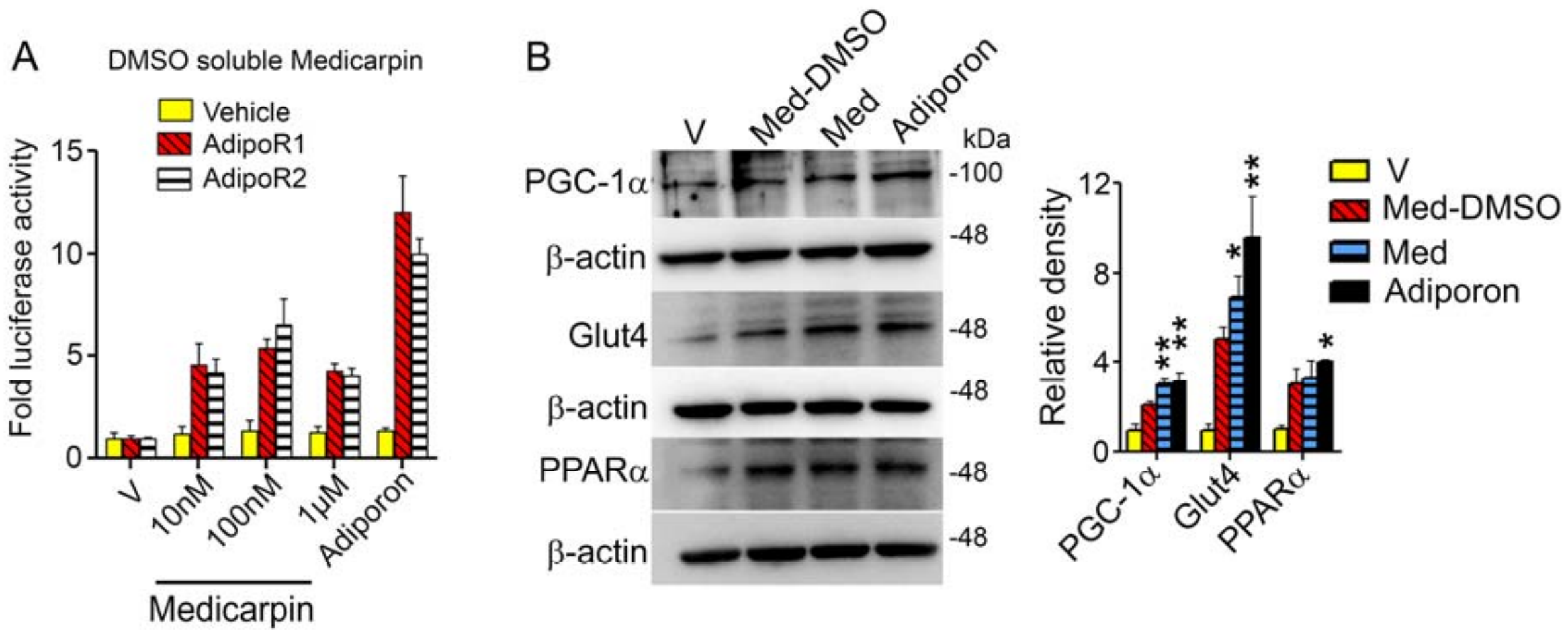

Supplementary Figure S1. Medicarpin (DMSO soluble) activates AdipoR1 and AdipoR2 and induces Adiponectin targets. A. HEK-293T cells transfected with PGC-1α-luc, empty vector or AdipoR1/AdipoR2 expression plasmids along with eGFPN1 were treated with vehicle (V), Medicarpin, or Adiporon at indicated concentrations for 24h and luciferase/GFP values were plotted as fold luciferase activity. **B.** C2C12 myotubes were treated as indicated with Medicarpin (100nM), Med (100nM) or, Adiporon (5µM) for 24h, and protein expression was analyzed by immunoblotting. β-actin was reprobed on the same blot. Right panel graph represents densitometry (mean ± SEM; n=3). $*p < 0.05$, $**p < 0.01$, $***p < 0.0001$. Statistical analyses were performed by 2-Way ANOVA (**A**), or 1-Way ANOVA (**B**) followed by Bonferroni's post-test.

Supplementary Figure S2

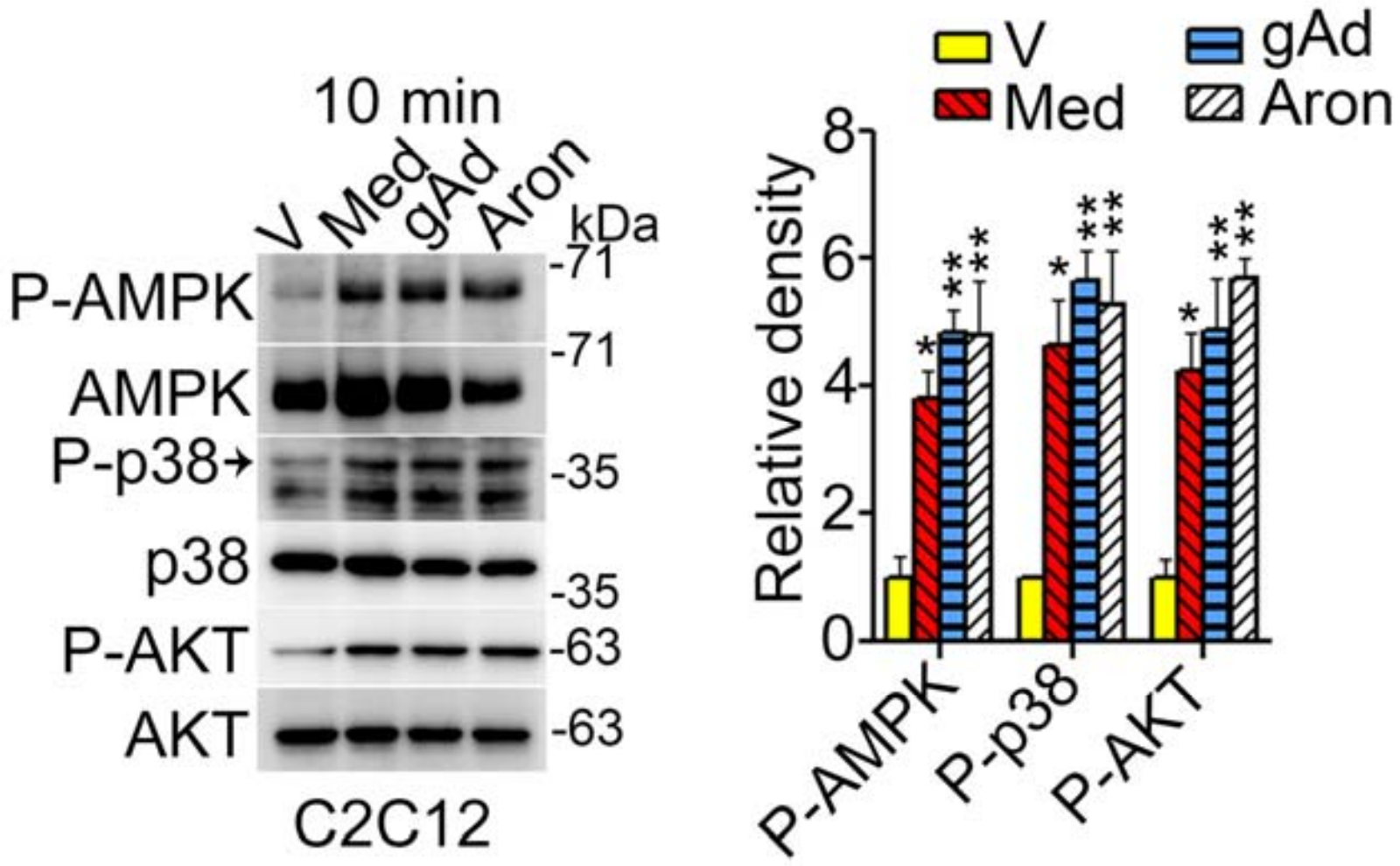

Supplementary Figure S2. Med treatment in differentiated C2C12 myotubes induces and activates factors associated with Adiponectin signaling pathways. C2C12 myotubes were treated with Med (100nM), gAd (1µg/mL), or Adiporon (5µM) for 10 min, and analyzed by immunoblotting. Graph represents mean ± SEM (n=3). Statistical analyses were performed by 1-Way ANOVA followed by Bonferroni's post-test. *p<0.05, **p<0.01, *** <0.0001. Aron; Adiporon, gAd: globular Adiponectin

Supplementary Figure S3

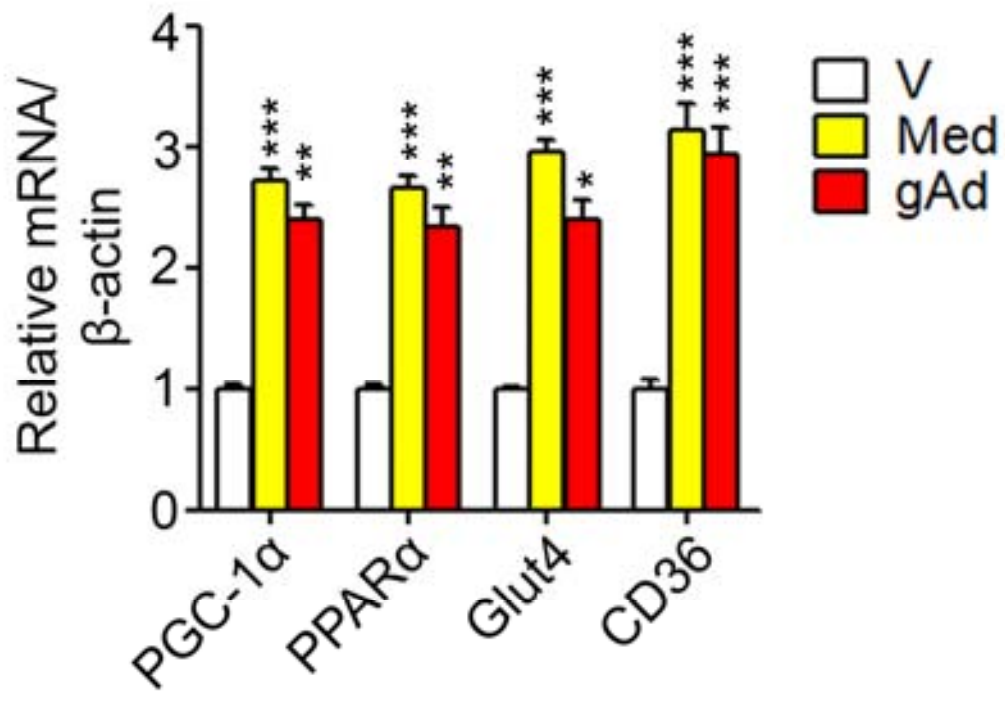

Supplementary Figure S3. Med treatment in differentiated C2C12 myotubes induces adiponectin target gene expressions. C2C12 myotubes were treated with Med (100nM), gAd (1µg/mL), or Adiporon (5µM) for 24h, and analyzed by qRT-PCR. Graph represents mean±SEM (n=3). Statistical analyses were performed by 1-Way ANOVA followed by Bonferroni's post-test (CD36), or Kruskal-Wallis test followed by Dunn's post-test (PGC-1α, Glut4, PPARα). *p<0.05, **p<0.01, ***p<0.0001.

Supplementary Figure S4

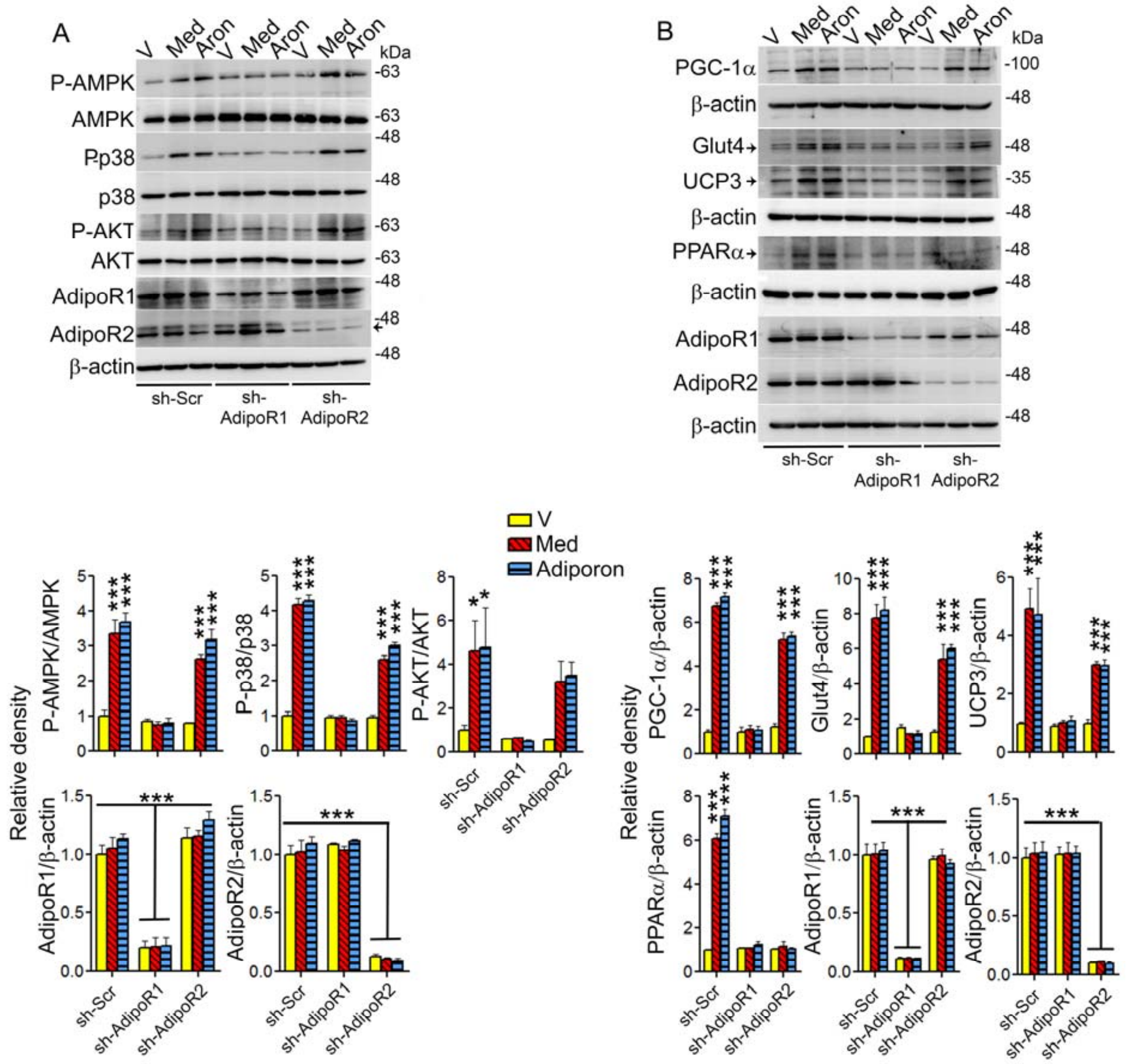

Supplementary Figure S4. Med treatment in C2C12 myotubes induces and activates factors involved in Adiponectin signaling pathways, and AdipoR1/R2 knockdown compromises it. C2C12 myotubes were infected with lentivirus (Set-2) containing sh-scr/AdipoR1/AdipoR2 shRNAs. 48h after infection, cells were treated with the indicated compounds for 10 min **(A)** or 24h **(B)**. Phospho-proteins were normalized with their respective total proteins (on same blot), for rest, β-actin (on same blot) was used as normalization control. Graphs represents mean ± SEM (n = 3). *p<0.05, **p<0.01, ***p<0.0001. Statistical analyses were performed by 2-Way ANOVA (**A-B**) followed by Bonferroni's post-test.

Supplementary Figure S5

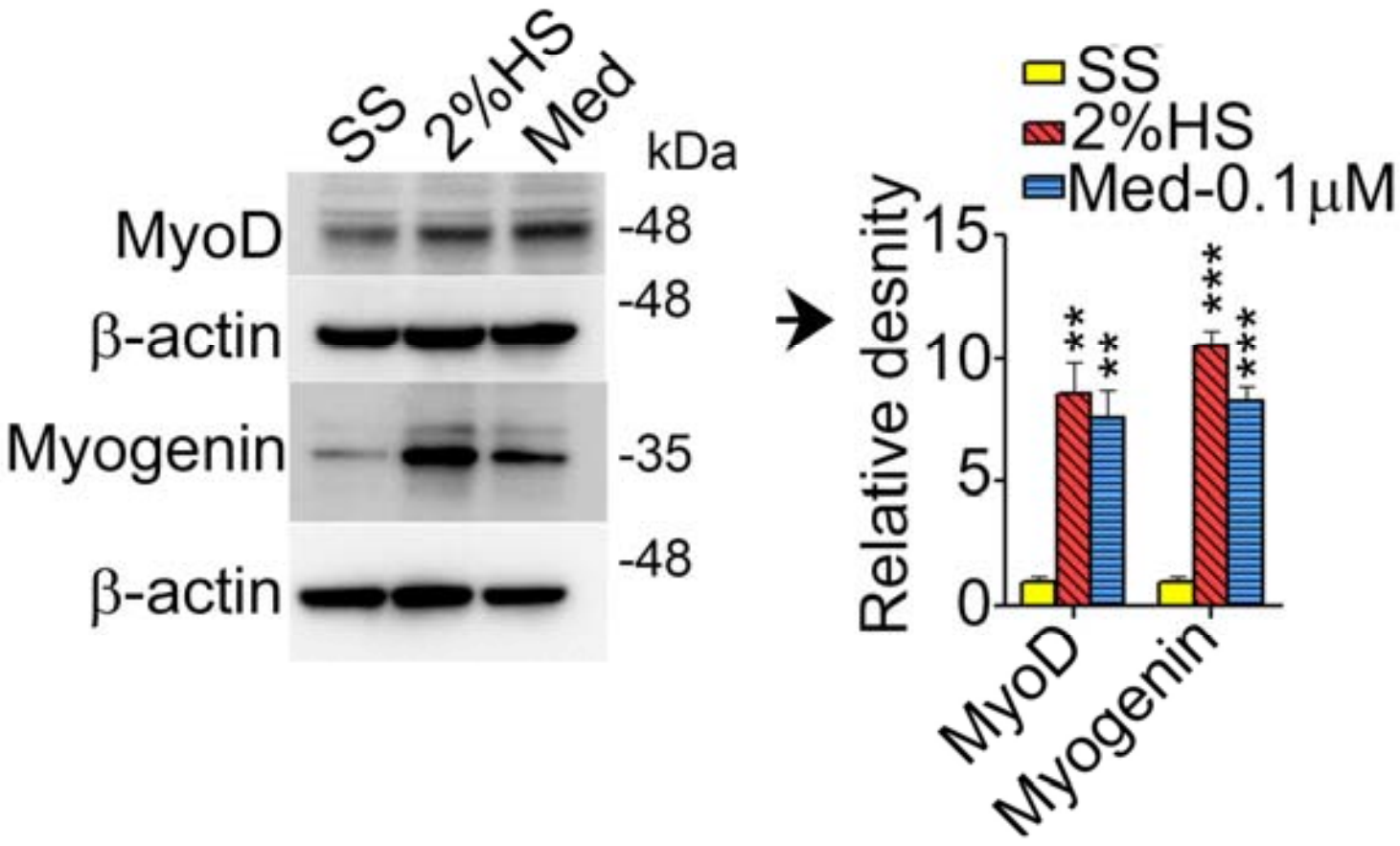

Supplementary Figure S5. Med promotes myogenic differentiation of C2C12 myoblasts. C2C12 myoblasts were treated as indicated for three days and analyzed by immunoblotting. Graph represents mean±SEM (n=3). Statistical analyses were performed by 1-Way ANOVA followed by Bonferroni's post-test. *p<0.05, **p<0.01, ***p<0.0001.

Supplementary Figure S6

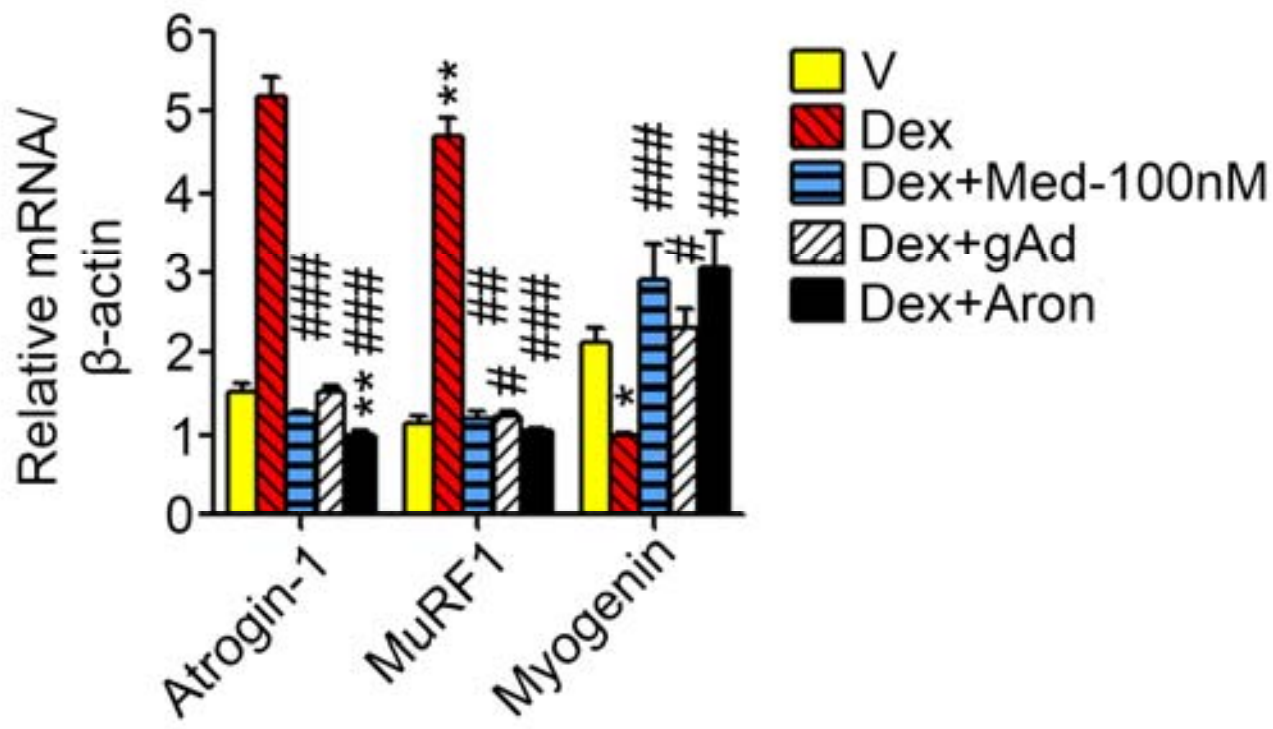

Supplementary Figure S6. Med reverses Dex induction of atrogenes and restores Dex-mediated suppression of myogenin mRNA expression in C2C12 myotubes. Graph represents (mean±SEM; n=3). *V vs treatment groups, #Dex vs treatment groups. *$p<0.05$, **$p<0.01$, ***$p<0.0001$. Statistical analyses were performed by Kruskal-Wallis test followed by Dunn's post-test. gAd: Globular Adiponectin, Aron: Adiporon. Control groups (V, Dex, Dex+gAd, Dex+Adiporon) are common with another study [**S23**].

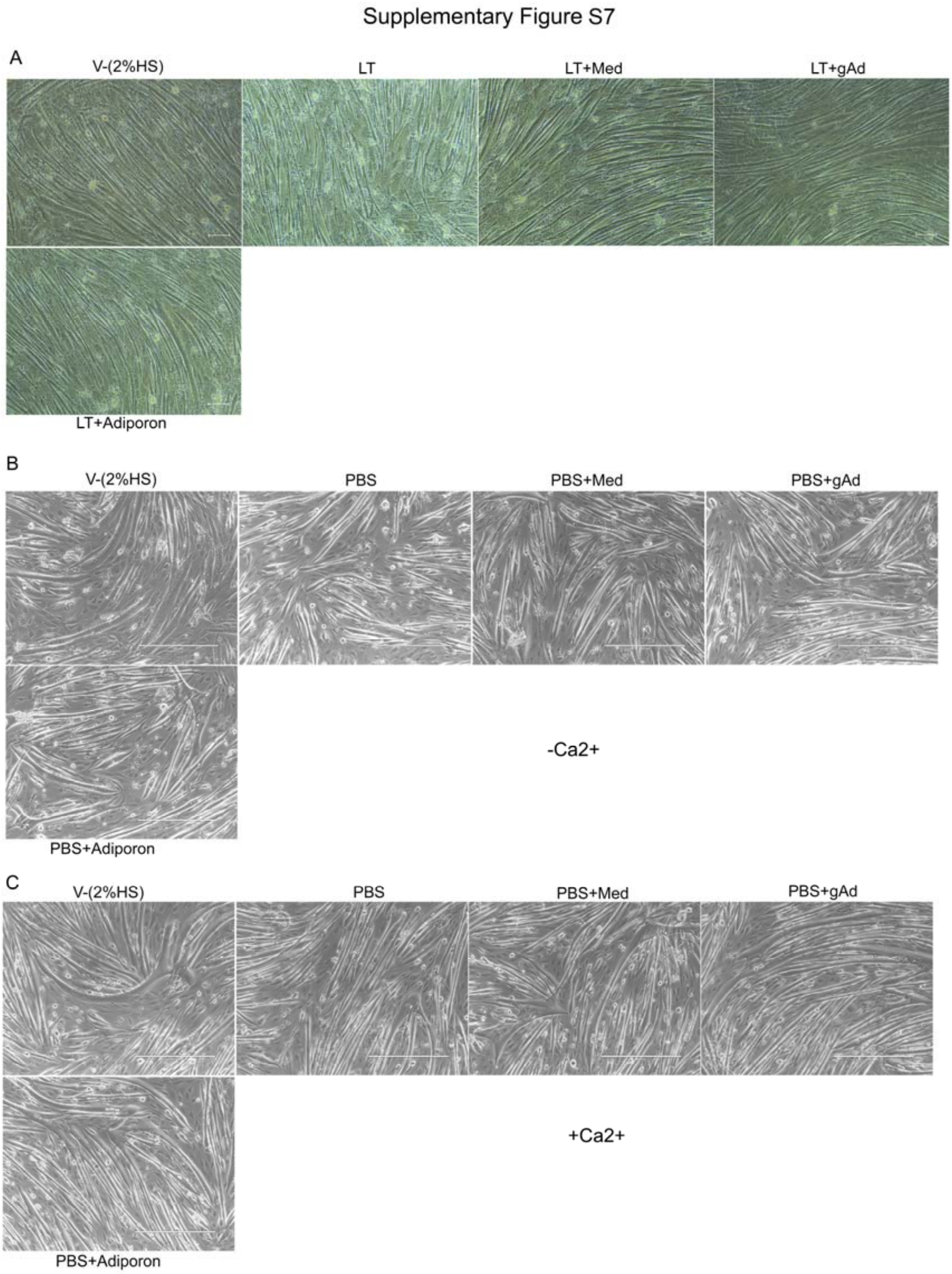

Supplementary Figure S7. Med mitigates (LPS + TNFα; LT), and nutrient-deficiency (PBS)–induced myotube shrinkage. Phase contrast microscopic images of LT-induced atrophy model (**A**), PBS without Ca^{2+} (**B**) and PBS with Ca^{2+} (**C**). Images in **A** were captured using a Nikon ECLIPSE Ts2 microscope (magnification: 100X; scale bar: 10µm, 18 fields/treatment group). Images in **B** and **C** were captured using EVOS FL Auto Imaging System (Life Technologies) (magnification: 100X, scale bar: 400µm, 18 fields/treatment group). Morphometric analyses of (**A-C**) are provided in **Fig. 3**.

Supplementary Figure S8

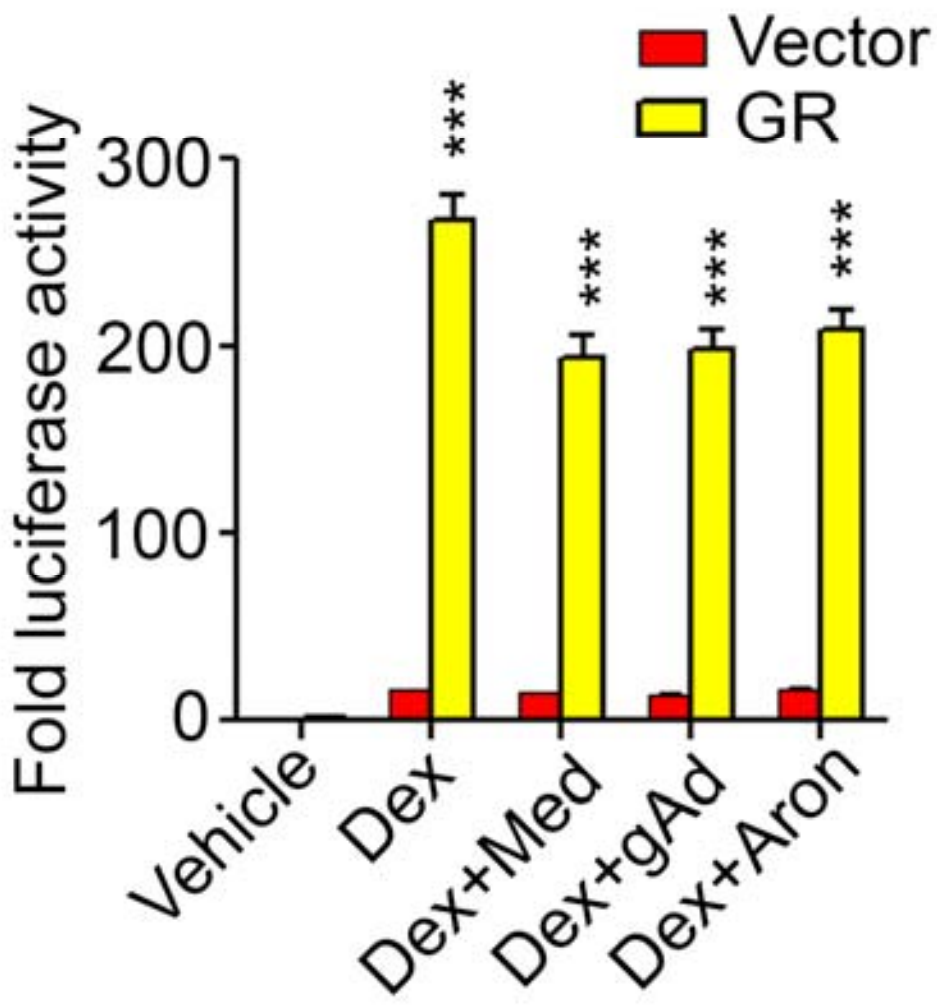

Supplementary Figure S8. Med does not suppress Dex-mediated GR activation in a GRE-Luciferase activity assay. HEK-293T cells seeded in 48-well plates were transfected with 200ng/well of empty vector/GR expression plasmid, GRE-luciferase reporter (200 ng/well), and EGFPN1 (100 ng/well). 24h post-transfection, cells were treated with V, Dex (10µM), or Dex+ test compounds for further 24h. Data represent mean ± SEM. *p< 0.05, ** < 0.01, ***p< 0.0001. *Vehicle-treated GR-transfected vs GR-transfected + test compounds. Statistical analysis was performed by 2-Way ANOVA followed by Bonferroni's post-test. gAd: Globular Adiponectin, Aron; Adiporon. Control groups (V, Dex, Dex+gAd, Dex+Adipron) are common with another study [**S23**].

Supplementary Figure S9

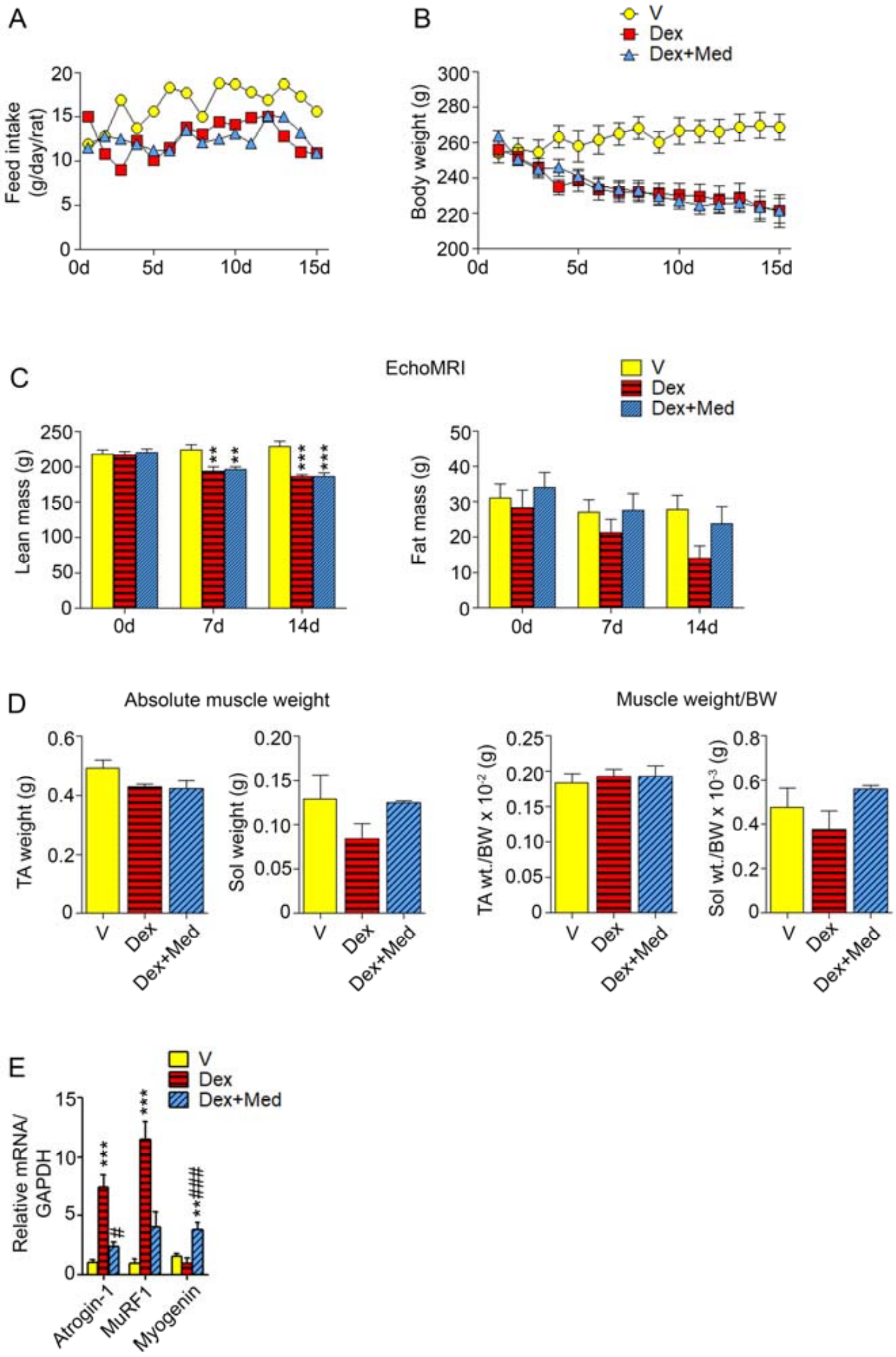

Supplementary Figure S9. Evaluation of the effect of Dex and Med on feed intake, body composition, muscle weight, and muscle mRNA expressions. Feed intake (**A**) body weight (**B**) of rats from indicated groups (n=6/group). **C.** Lean mass and Fat mass (in grams) of rats from indicated groups (n=6/group) measured using an EchoMRI-500 Body Composition Analyzer (EchoMRI Corporation Pvt. Ltd, Singapore). **D. Absolute and relative muscle weights of TA and Soleus** muscles from left hindlimbs (n=6/group). **E.** Transcript levels of Atrogin-1, MuRF1 and myogenin in GN muscle of rats from indicated treatment groups (n=3 in triplicates). All graphs represent mean ± SEM. *V vs treatment groups, #Dex vs treatment groups. *#p < 0.05, **##p < 0.01, ***##p < 0.0001. Statistical analysis performed using 1-Way ANOVA followed by Bonferroni's post-test for **C**, **D**, **E**. For Atrogin1 and MuRF1,

except for myogenin in **E**. Kruskal-Wallis test was performed followed by Dunn's post-test. Control groups (V, Dex) are common with another study [**S23**].

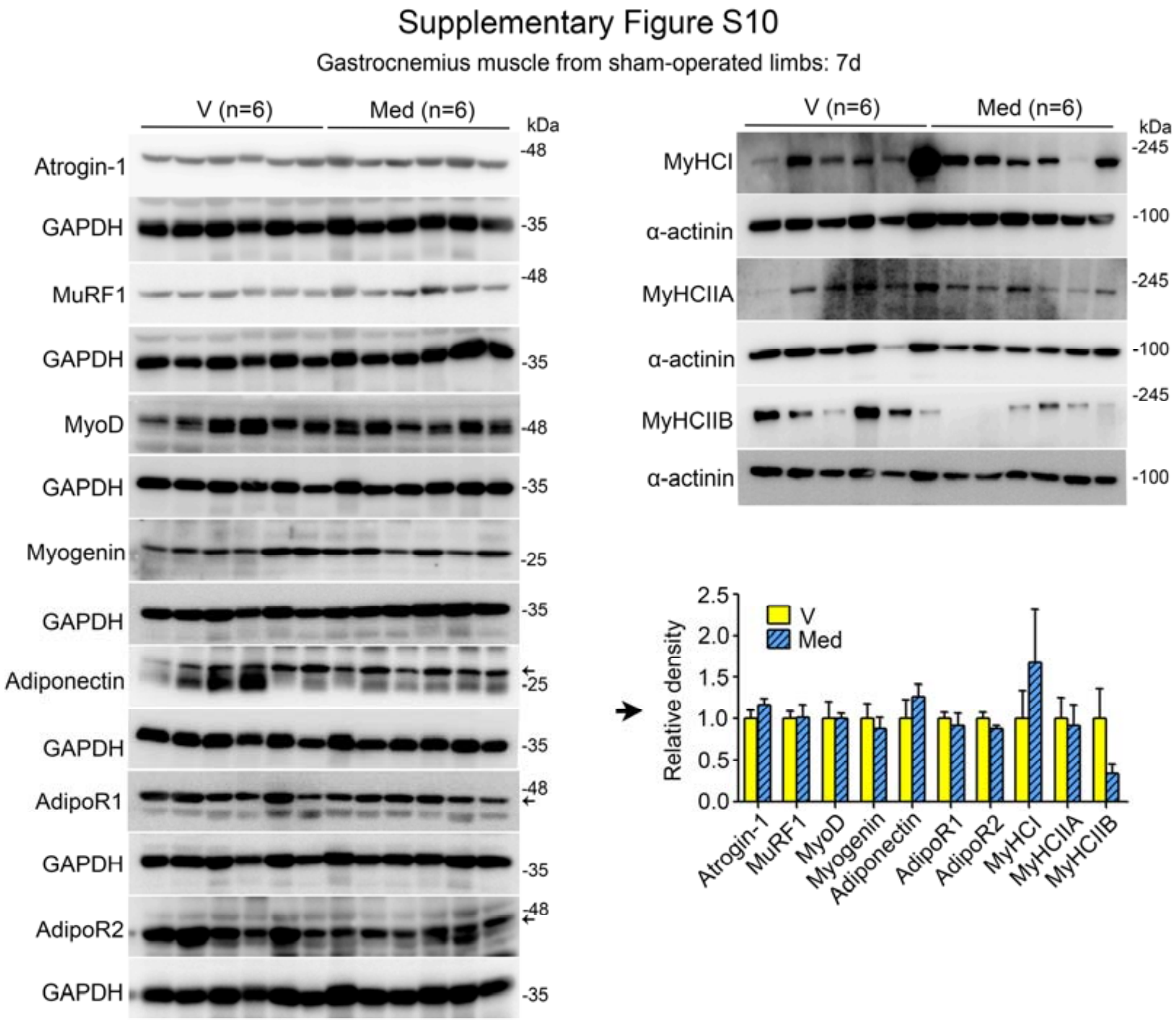

Supplementary Figure S10. Med treatment does not significantly alter expressions of the proteins examined in sham-operated GN muscles. Immunoblot panels represent expression of indicated proteins in GN muscles from sham-operated limbs of vehicle (n=6) or Med (n=6) -treated rats corresponding to the denervation experiment (**main Figure 6**). Graph represents densitometric analysis (mean±SEM). MyHC-I, MyHC-IIA, and MyHC-IIB were normalized with α-actinin, and the rest were normalized with GAPDH. Statistical analysis was performed by unpaired, two-tailed Student's t-test, except for MyoD and MyHC-IIB, where Mann-Whitney test was used. F test was used to assess intra-group variances.

Supplementary Figure S11

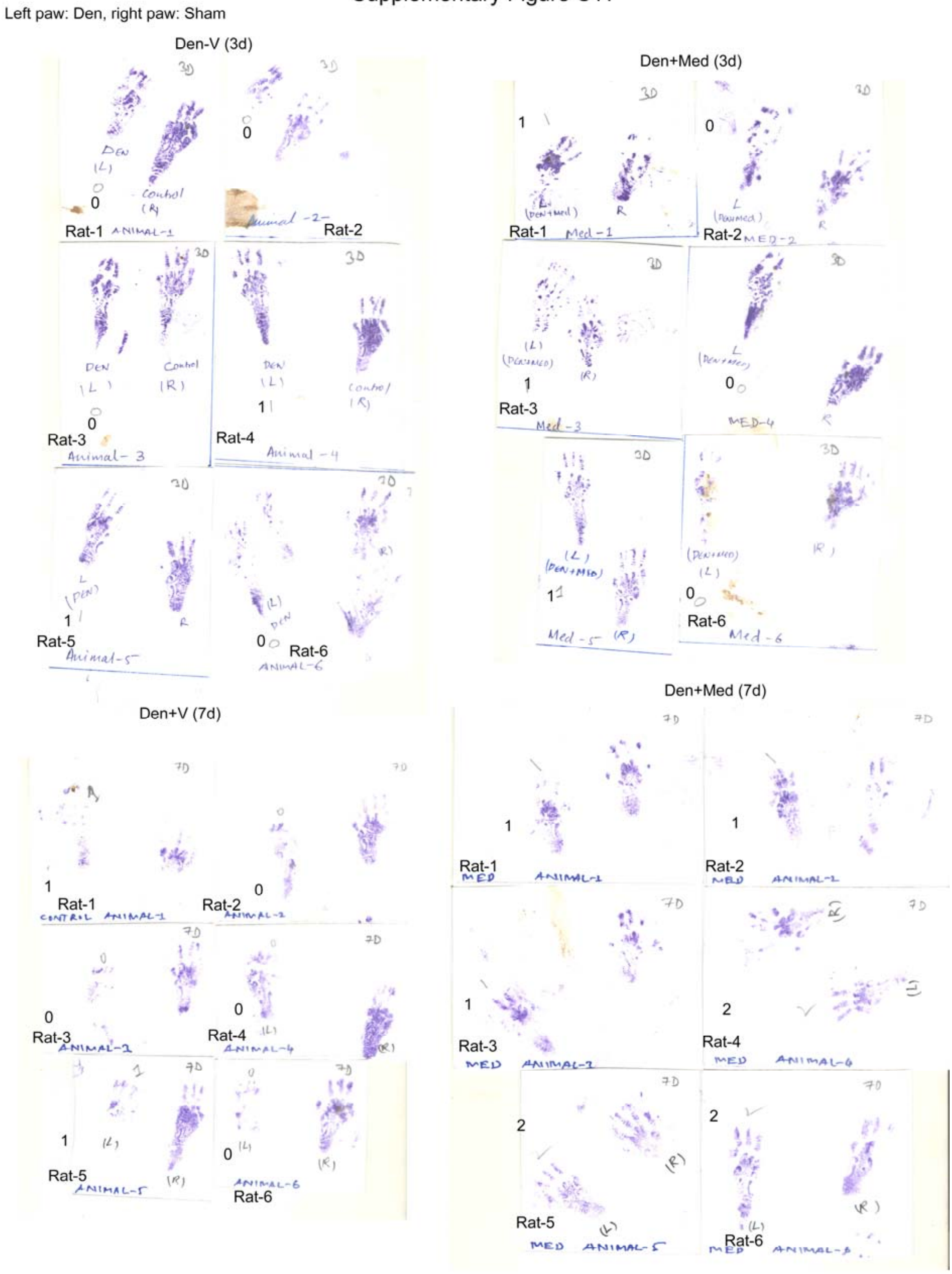

Supplementary Figure S11. Paw print images from day 3 and day 7, post-denervation. Paw prints from individual rats are labeled along with their respective scores. Left hindlimbs in each case was denervated, and the right limb served as sham and was taken as benchmark (score: 2) for full opening of the paws. Corresponding graphs are in the **main Figure 6F**.

Supplemental References

S1. Langen RC, Gosker HR, Remels AH, Schols AM. Triggers and mechanisms of skeletal muscle wasting in chronic obstructive pulmonary disease. Int J Biochem Cell Biol. 2013;45:2245-56. doi:S1357-2725(13)00199-4 [pii]
10.1016/j.biocel.2013.06.015
S2. Lecker SH, Jagoe RT, Gilbert A, Gomes M, Baracos V, Bailey J, et al. Multiple types of skeletal muscle atrophy involve a common program of changes in gene expression. FASEB J. 2004;18:39-51. doi:18/1/39 [pii]
10.1096/fj.03-0610com
S3. Yang J, Cao RY, Li Q, Zhu F. Muscle Atrophy in Cancer. Adv Exp Med Biol. 2018;1088:329-46. doi:10.1007/978-981-13-1435-3_15
S4. Chen L, Zhang, H., Chi, M., Yang, Q., & Guo, C. Drugs for the Treatment of Muscle Atrophy. IntechOpen. 2021;doi: doi: 10.5772/intechopen.93503
S5. Achari AE, Jain SK. Adiponectin, a Therapeutic Target for Obesity, Diabetes, and Endothelial Dysfunction. Int J Mol Sci. 2017;18:doi:10.3390/ijms18061321
1321
ijms18061321 [pii]
ijms-18-01321 [pii]
S6. Nguyen TMD. Adiponectin: Role in Physiology and Pathophysiology. Int J Prev Med. 2020;11:136. doi:10.4103/ijpvm.IJPVM_193_20
136
IJPVM-11-136 [pii]
S7. Gamberi T, Magherini F, Modesti A, Fiaschi T. Adiponectin Signaling Pathways in Liver Diseases. Biomedicines. 2018;6:doi:10.3390/biomedicines6020052
52
biomedicines6020052 [pii]
biomedicines-06-00052 [pii]
S8. Akimoto M, Maruyama R, Kawabata Y, Tajima Y, Takenaga K. Antidiabetic adiponectin receptor agonist AdipoRon suppresses tumour growth of pancreatic cancer by inducing RIPK1/ERK-dependent necroptosis. Cell Death Dis. 2018;9:804. doi:10.1038/s41419-018-0851-z
804

10.1038/s41419-018-0851-z [pii]
851 [pii]
S9. Jortay J, Senou M, Abou-Samra M, Noel L, Robert A, Many MC, et al. Adiponectin and skeletal muscle: pathophysiological implications in metabolic stress. Am J Pathol. 2012;181:245-56. doi:10.1016/j.ajpath.2012.03.035
S0002-9440(12)00324-0 [pii]
S10. Khan MP, Singh AK, Joharapurkar AA, Yadav M, Shree S, Kumar H, et al. Pathophysiological Mechanism of Bone Loss in Type 2 Diabetes Involves Inverse Regulation of Osteoblast Function by PGC-1alpha and Skeletal Muscle Atrogenes: AdipoR1 as a Potential Target for Reversing Diabetes-Induced Osteopenia. Diabetes. 2015;64:2609-23. doi:10.2337/db14-1611
db14-1611 [pii]
S11. Shinohara I, Kataoka T, Mifune Y, Inui A, Sakata R, Nishimoto H, et al. Influence of adiponectin and inflammatory cytokines in fatty degenerative atrophic muscle. Sci Rep. 2022;12:1557. doi:10.1038/s41598-022-05608-x
1557
10.1038/s41598-022-05608-x [pii]
5608 [pii]
S12. Schneider CA, Rasband WS, Eliceiri KW. NIH Image to ImageJ: 25 years of image analysis. Nat Methods. 2012;9:671-5. doi:10.1038/nmeth.2089
S13. Park SJ, Baek JY, Wei S, Lee JY, Lee Y, Lee SH, et al. Elevated Circulating Ceramides 18:0 and 24:1 as a Risk Factor for Sarcopenia: In Vitro, Animal, and Clinical Evidence. J Cachexia Sarcopenia Muscle. 2026;17:e70310. doi:10.1002/jcsm.70310
e70310
JCSM70310 [pii]
S14. Brun A, Mougeot G, Denis P, Collin ML, Pouchin P, Montaurier C, et al. A new bio imagery user-friendly tool for automatic morphometry measurement on muscle cell cultures and histological sections. Sci Rep. 2024;14:3108. doi:10.1038/s41598-024-53658-0
3108
10.1038/s41598-024-53658-0 [pii]
53658 [pii]

S15. Balasubramanian P, Schaar AE, Gustafson GE, Smith AB, Howell PR, Greenman A, et al. Adiponectin receptor agonist AdipoRon improves skeletal muscle function in aged mice. Elife. 2022;11:doi:10.7554/eLife.71282
e71282
71282 [pii]
S16. Iwabu M, Okada-Iwabu M, Tanabe H, Ohuchi N, Miyata K, Kobori T, et al. AdipoR agonist increases insulin sensitivity and exercise endurance in AdipoR-humanized mice. Commun Biol. 2021;4:45. doi:10.1038/s42003-020-01579-9
45
10.1038/s42003-020-01579-9 [pii]
1579 [pii]
S17. Halling JF, Pilegaard H. PGC-1alpha-mediated regulation of mitochondrial function and physiological implications. Appl Physiol Nutr Metab. 2020;45:927-36. doi:10.1139/apnm-2020-0005
S18. Kim YI, Lee H, Nirmala FS, Seo HD, Ha TY, Jung CH, et al. Antioxidant Activity of Valeriana fauriei Protects against Dexamethasone-Induced Muscle Atrophy. Oxid Med Cell Longev. 2022;2022:3645431. doi:10.1155/2022/3645431
3645431
S19. Wang Y, Zhang X, Shao J, Liu H, Liu X, Luo E. Adiponectin regulates BMSC osteogenic differentiation and osteogenesis through the Wnt/beta-catenin pathway. Sci Rep. 2017;7:3652. doi:10.1038/s41598-017-03899-z
3652
10.1038/s41598-017-03899-z [pii]
3899 [pii]
S20. Hargreaves M, Spriet LL. Skeletal muscle energy metabolism during exercise. Nat Metab. 2020;2:817-28. doi:10.1038/s42255-020-0251-4
10.1038/s42255-020-0251-4 [pii]
S21. Roy RR, Gardiner PF, Simpson DR, Edgerton VR. Glucocorticoid-induced atrophy in different fibre types of selected rat jaw and hind-limb muscles. Arch Oral Biol. 1983;28:639-43. doi:10.1016/0003-9969(83)90012-2
S22. Noh KK, Chung KW, Choi YJ, Park MH, Jang EJ, Park CH, et al. beta-Hydroxy beta-methylbutyrate improves dexamethasone-induced muscle atrophy by modulating the muscle

degradation pathway in SD rat. PLoS One. 2014;9:e102947. doi:10.1371/journal.pone.0102947
e102947
PONE-D-14-11110 [pii]
S23. Moin MR, Pal S, Das S, Awasthi, P, Talukdar A, Kumar A, et al. Selective Small-Molecule AdipoR1 Agonist 3-Hydroxy Pterocarpan Salt (CDRI-1709S) Ameliorates Skeletal Muscle Atrophy. J Cachexia Sarcopenia Muscle 2026; DOI: 10.1002/jcsm.70328
