# Supplementary material for: A Novel Small‐Molecule Dual Adiponectin Receptor 1/2 Agonist Induces Myogenesis and Ameliorates Skeletal Muscle Atrophy
Source: J Cachexia Sarcopenia Muscle. 2026 Aug 24;17(4):e70364. doi: 10.1002/jcsm.70364 (PMC13502058; doi:10.1002/jcsm.70364)

Supplementary Information: Chemical synthesis

1. **Synthesis of medicarpin (6) and its water-soluble sodium salt Med (7):**

Compound **6** was synthesized following the methodology outlined in our previous report (Goel et al., 2012), and as shown in Scheme-1. Briefly, the Friedel-Crafts acylation of 2-(2,4-dimethoxyphenyl)acetic acid and resorcinol in the presence of boron trifluoride provides 1-(2,4-dihydroxyphenyl)-2-(2,4-dimethoxyphenyl)ethan-1-one (3). This (compound **3**) on treatment with methanesulfonyl chloride and DMF in the presence of Lewis acid $BF_3.OEt_2$ form isoflavone **4**. Further, selective demethylation of **4** using aluminium trichloride in ACN reflux results in the formation of 2'-hydroxy isoflavone **5**, which on one-pot reduction and by cyclization using $NaBH_4$ furnished medicarpin (**6**) in good yield. Finally, we synthesized the water-soluble sodium salt **7** in 74% yield, by treatment of medicarpin (**6**) with aq. NaOH at room temperature. Methods of synthesis of each compound, their spectroscopic characterization data and the HPLC purity analysis data of compound **7** (purity=99.7 %) are provided below.

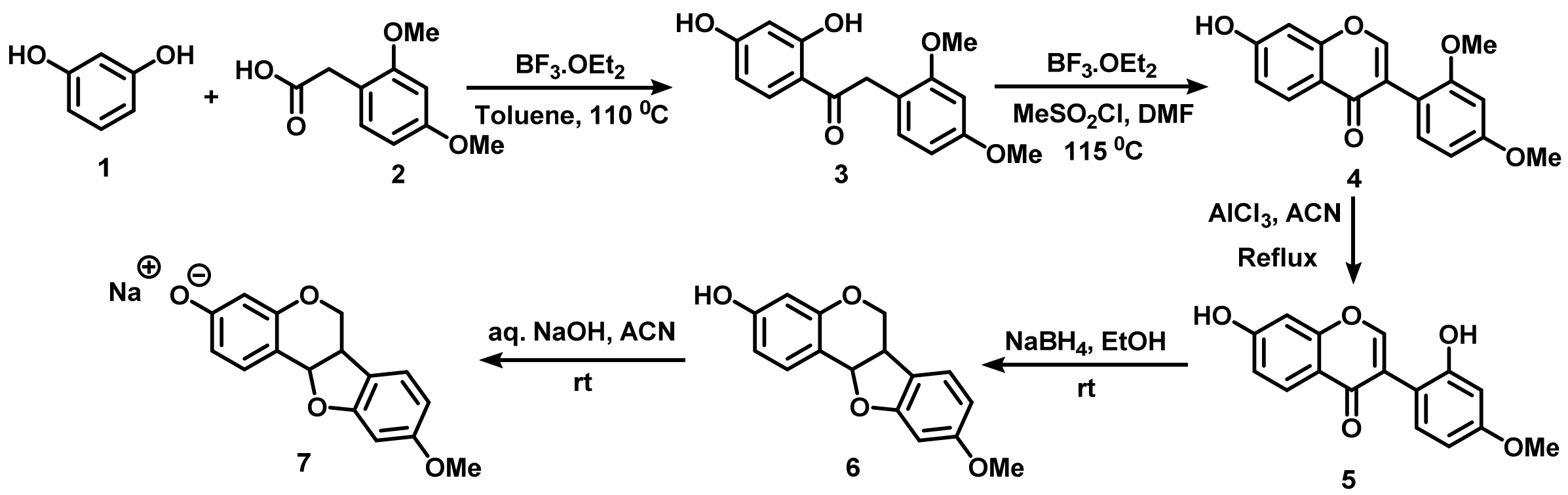

Scheme-1: Synthesis of medicarpin **(6)** and its water-soluble sodium salt Med **(7).**

Purity analysis of Med (7):

To assess the purity of synthesized compound **7**, a high-performance liquid chromatographic method was developed using an (1:1) ACN/Water eluting system on a reverse-phase Luna 5 µm C18(2) 100 Å LC column with flow rate 0.400 mL/min. Determined retention time of water-soluble Med **(7)** was 24.36 min and the purity was 99.7 %.

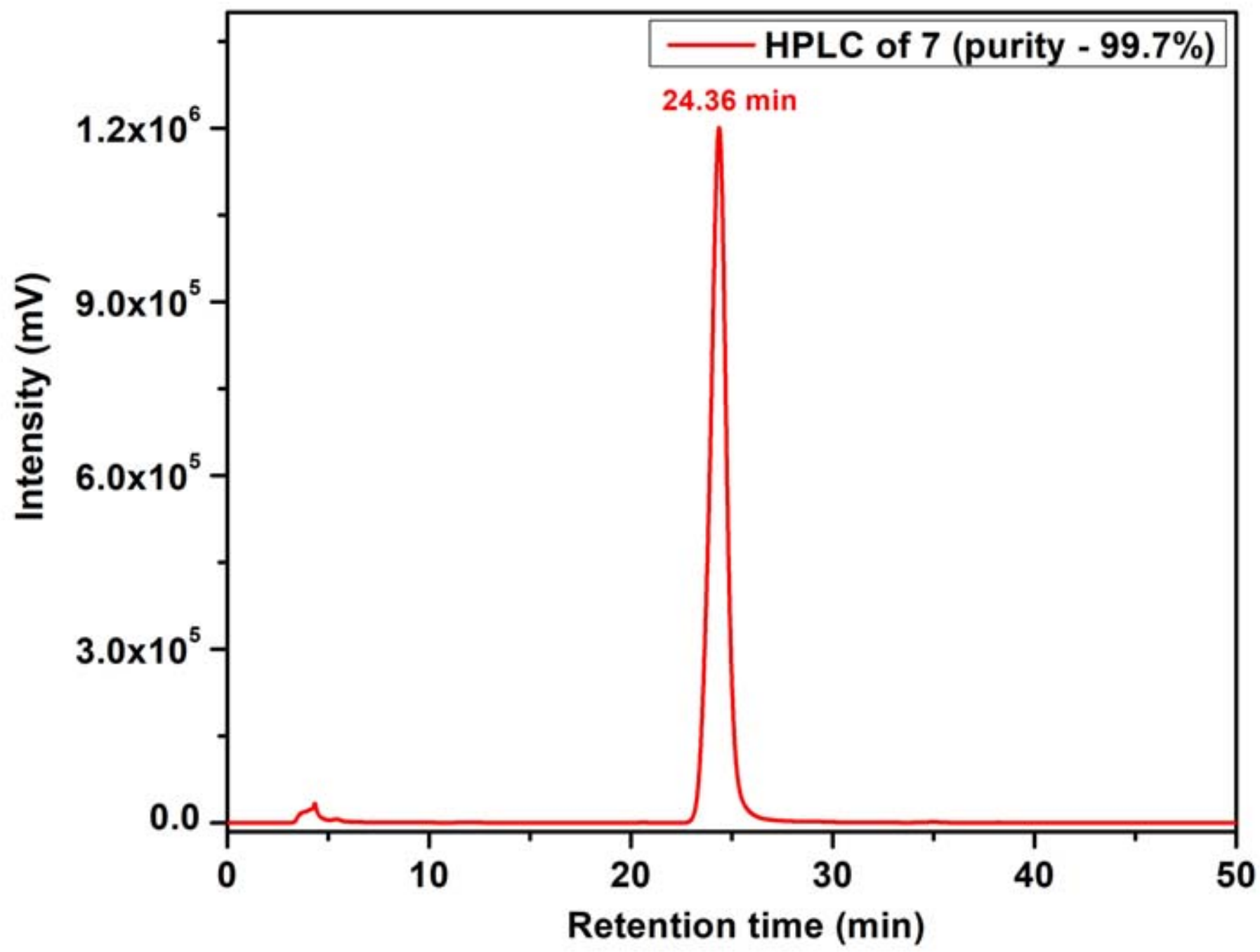

Assessment of purity: HPLC of compound **7** (Med).

Method of synthesis :

Synthesis of 1-(2,4-dihydroxyphenyl)-2-(2,4-dimethoxyphenyl)ethan-1-one (3). In an oven-dried 250 mL round-bottom flask containing 100 mL of toluene, resorcinol **1** (5 g, 1 equiv) and 2-(2,4-dimethoxyphenyl)acetic acid **2** (9.8 g, 1.1 equiv) were stirred at 0°C. In the above-stirred solution $BF_3.OEt_2$ (11.7 mL, 2 equiv) was added dropwise, and the resulting mixture was refluxed at 110°C for 5 h. After completion, the reaction was cooled to rt, concentrated under reduced pressure and to the residue, added 200 mL of water. After neutralization using saturated solution of $NaHCO_3$, precipitated solid was filtered and washed with chlorobenzene to furnish the desired **3** (11.8 g, 90% yield) as a brown solid. Mp: 150–151°C; R_f 0.4 ($CHCl_3$); ^{1}H NMR (400 MHz, DMSO-d_6): δ 12.51(s, 1H), 10.60 (s, 1H), 7.90 (d, J = 8.9 Hz, 1H), 7.05 (d, J = 8.3 Hz, 1H), 6.55 (d, J = 2.4 Hz, 1H), 6.47 (dd, J = 8.3, 2.4 Hz, 1H), 6.38 (dd, J = 8.9, 2.4 Hz, 1H), 6.25 (d, J = 2.4 Hz, 1H), 4.14 (s, 2H), 3.75 (s, 3H), 3.71 (s, 3H); ^{13}C NMR (100 MHz, DMSO-d_6):δ 202.3, 164.7, 164.3, 159.7, 157.9, 133.0, 131.4, 115.5, 112.3, 108.1, 104.6, 102.4, 98.4, 55.4, 55.1, 38.3; HRMS (ESI) Calcd for $C_{16}H_{17}O_5$ $[M+H]^+$: 289.1076, found: 289.1071.

Synthesis of 3-(2,4-dimethoxyphenyl)-7-hydroxy-4H-chromen-4-one (4). In a 250 mL round-bottom flask, **3** (8 g, 1 equiv) was dissolved with 100 mL DMF and cooled to 0°C. To the solution of **3**, dropwise $BF_3.OEt_2$ (7.2 mL, 2 equiv) was added. After 30 mins, mesyl chloride (4.2 mL, 2 equiv) was added and the reaction was allowed to be stirred at 115°C for 4 h. The reaction was cooled to rt, poured into 300 mL of water, and neutralized using saturated solution of $NaHCO_3$. The precipitate was filtered and washed with chlorobenzene and tert-butyl alcohol to afford **4** (6.7 g, 81% yield) as a pale red solid. Mp: 238-239 °C; R_f 0.50 (20:1 $CHCl_3$:CH_3OH); ^1H NMR (400 MHz, DMSO-d_6) δ 10.76 (s, 1H), 8.11 (s, 1H), 7.91 (d, J = 8.7 Hz, 1H), 7.12 (d, J = 8.3 Hz, 1H), 6.92 (dd, J = 8.7, 2.3 Hz, 1H), 6.85 (d, J = 2.3 Hz, 1H), 6.62 (d, J = 2.4 Hz, 1H), 6.56 (dd, J = 8.4, 2.4 Hz, 1H), 3.79 (s, 3H), 3.69(s, 3H); ^{13}C NMR (100 MHz, DMSO-d_6) δ 174.4, 162.4, 160.7, 158.4, 157.5, 153.9, 132.0, 127.2, 121.6, 116.5, 115.0, 113.5, 104.6, 102.1, 98.6, 55.5, 55.3; HRMS (ESI) Calcd for $C_{17}H_{15}O_5$ $[M+H]^+$: 299.0919, found: 299.0909.

Synthesis of 7-hydroxy-3-(2-hydroxy-4-methoxyphenyl)-4H-chromen-4-one (5). In an oven-dried 250 ml round bottom flask, **4** (3 g, 1 equiv) was dissolved in 150 mL ACN and cooled to 0°C. To the solution of **4**, $AlCl_3$ (8 g, 6 equiv) was added in portions over 30 min and then the resulting mixture was refluxed for 24 h. The reaction was cooled to rt and quenched by the addition of 450 ml water. The resulting precipitate was filtered to furnish the desired **5** (2.4 g, 84% yield) as a brown solid. Mp: 211–212 °C; R_f 0.5 (3:2 Hexane:$CHCl_3$); ^1H NMR (400 MHz, DMSO-d_6): δ 10.78 (s, 1H), 9.42 (s, 1H), 8.17 (s, 1H), 7.94 (d, J = 8.8 Hz, 1H), 7.10 (d, J = 8.2 Hz, 1H), 6.94 (dd, J = 8.7, 2.3 Hz, 1H), 6.87 (d, J = 2.2 Hz, 1H), 6.47 – 6.42 (m, 2H), 3.73 (s, 3H); ^{13}C NMR (100 MHz, DMSO-d_6): δ 175.0, 162.5, 160.2, 157.5, 156.5, 154.5, 132.2, 127.2, 121.5, 116.5, 115.1, 111.9, 104.5, 102.1, 101.6, 55.0; HRMS (ESI) Calcd for $C_{16}H_{13}O_5$ $[M+H]^+$: 285.0763, found: 285.0757.

Synthesis of 9-methoxy-6a,11a-dihydro-6H-benzofuro[3,2-c]chromen-3-ol (6). To the solution of compound **5** (3.5 g, 1 equiv) in ethanol was added solid $NaBH_4$ (2.8 g, 6 equiv) at 0°C. The cooling bath was then removed, and the reaction stirred at room temperature for 12 h. The solvent of reaction mixture was removed under reduced pressure and quenched by addition of water. The reaction was then neutralized with dil. HCl, and the resulting precipitate was filtered. The crude product was purified by silica gel column chromatography (99:1 = $CHCl_3$:CH_3OH) to give **6** (2 g, 61% yield) as white solid. Mp: 189–190 °C; R_f 0.6 (49:1 $CHCl_3$:CH_3OH); ^1H NMR (400 MHz, DMSO-d_6): δ 9.62 (s, 1H), 7.26 (d, J = 8.4 Hz,

1H), 7.22 (d, J = 8.0 Hz, 1H), 6.49 – 6.40 (m, 3H), 6.28-6.24 (m, 1H), 5.55 – 5.51 (m, 1H), 4.27 – 4.18 (m, 1H), 3.69 (s, 3H), 3.64 – 3.54 (m, 2H); ^{13}C NMR (100 MHz, DMSO-d_6): δ 160.5, 160.3, 158.7, 156.3, 132.1, 125.1, 119.4, 111.2, 109.7, 105.9, 102.8, 96.3, 78.1, 65.8, 55.3, 38.8; HRMS (ESI) Calcd for $C_{16}H_{15}O_4$ [M+H]$^+$: 271.0970, found: 271.0965.

Synthesis of sodium 9-methoxy-6a,11a-dihydro-6H-benzofuro[3,2-c]chromen-3-olate (7): In a 25 mL round-bottom flask, **6** (200 mg, 1 equiv) was dissolved in 5 mL acetonitrile. To the solution of **6**, aq. NaOH (40 mg, 2 equiv) was added at room temperature and the mixture was stirred for 15 minutes. The resulting precipitate was filtered and washed with acetonitrile to furnish the desired salt **7** (161 mg, 74% yield) as a pale pink solid. Mp: 176–177 °C; ^{1}H NMR (400 MHz, D_2O): δ 7.34 (d, J = 8.3 Hz, 1H), 7.27 (dd, J = 8.7, 1.6 Hz, 1H), 6.63 (d, J = 8.3 Hz, 1H), 6.52 (s, 1H), 6.42 (d, J = 8.6 Hz, 1H), 6.16-6.11 (m,1H), 5.73 (d, J = 7.3 Hz, 1H), 4.32-4.24 (m, 1H), 3.82 (s, 3H), 3.81-3.69 (m, 2H); ^{13}C NMR (100 MHz, D_2O): δ 168.9, 160.1, 159.5, 156.3, 131.8, 125.4, 120.3, 114.8, 106.8, 106.1, 105.2, 96.6, 80.2, 66.3, 55.6, 39.2; HRMS (ESI) Calcd for $C_{16}H_{15}O_4$ [M-Na-2H]$^+$: 271.0976, found: 271.0960.

NMR:

A: ^{1}H NMR of Compound-**3**

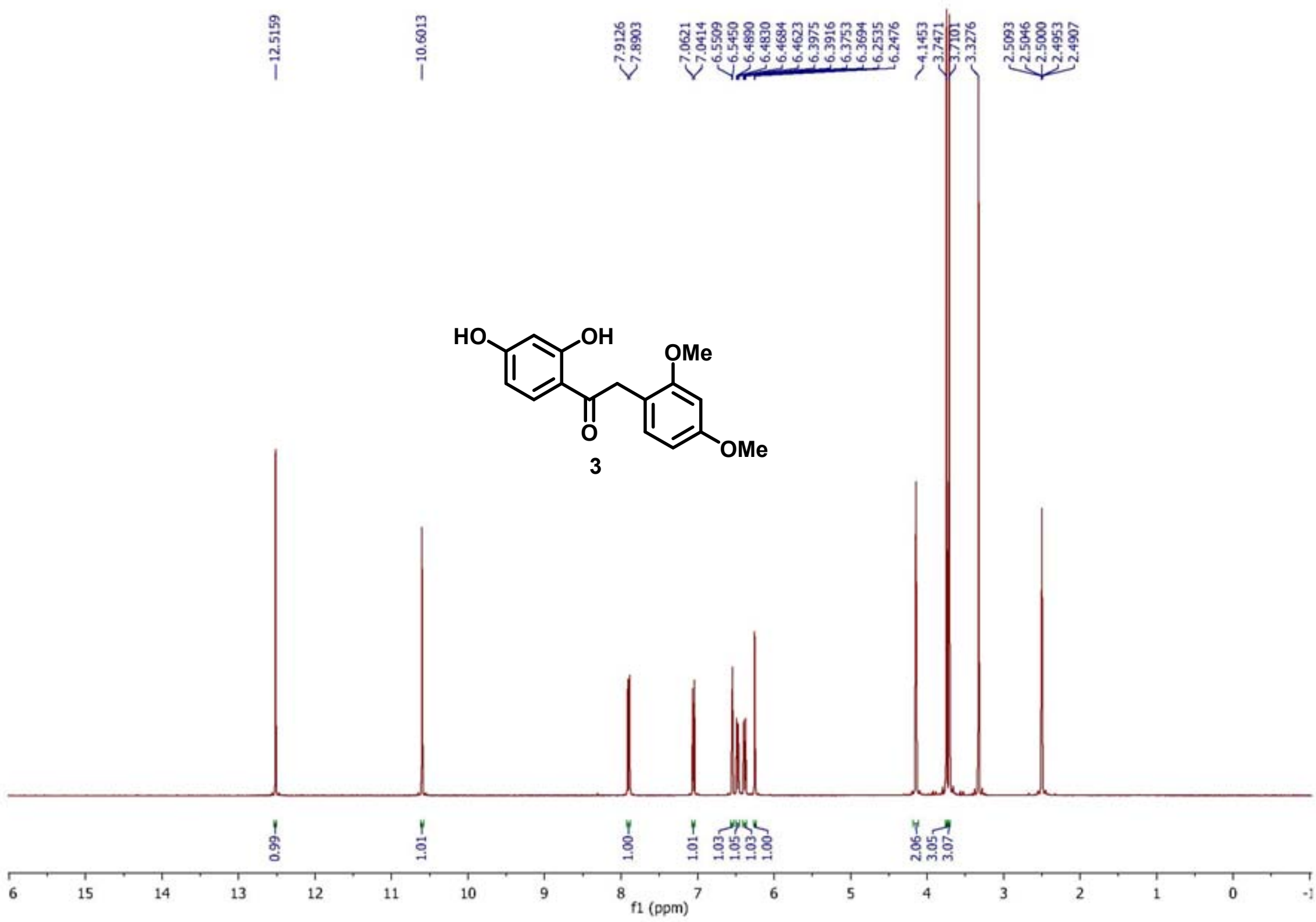

B: ^{13}C NMR of Compound-**3**

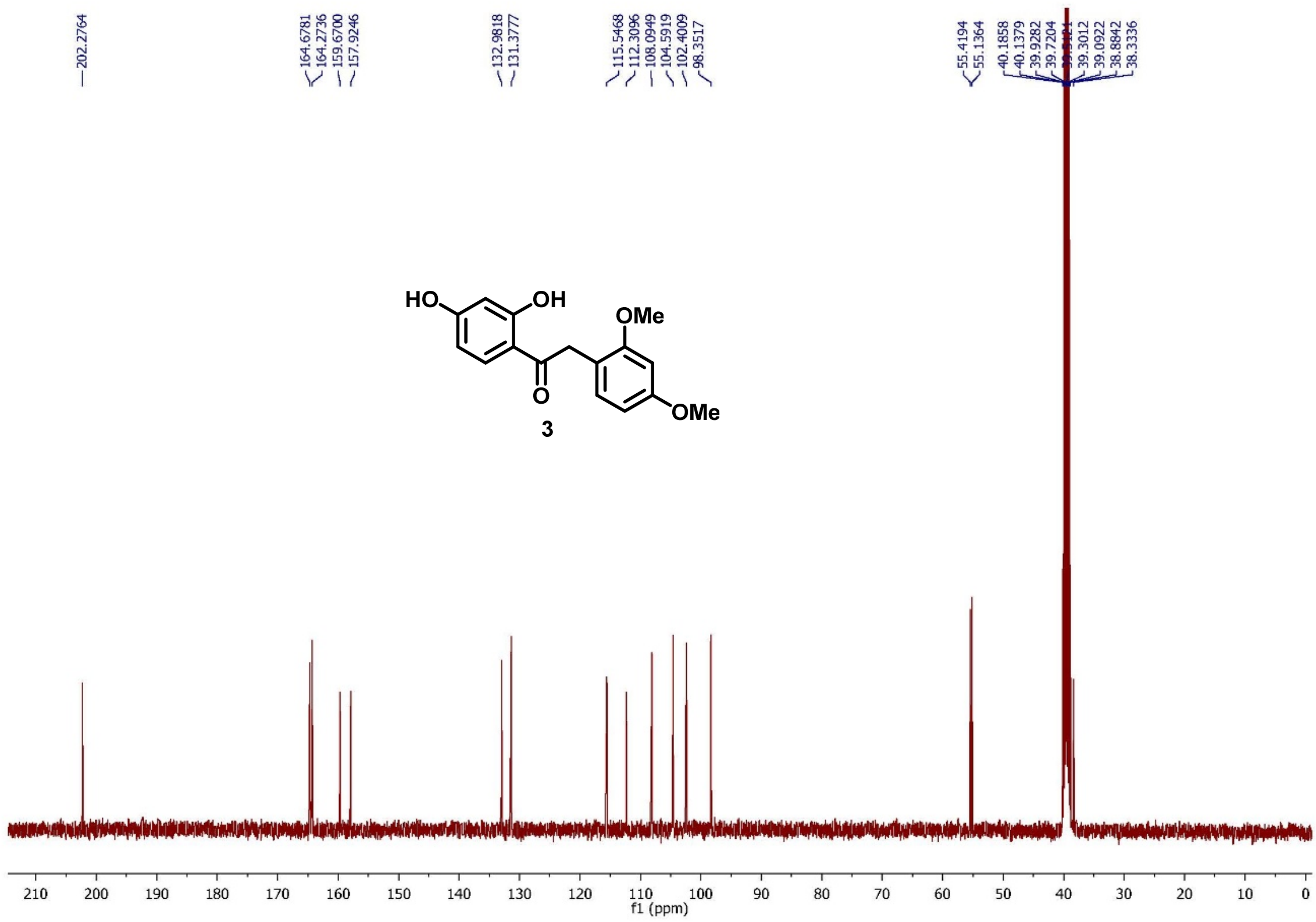

C: ^{1}H NMR of Compound-**4**

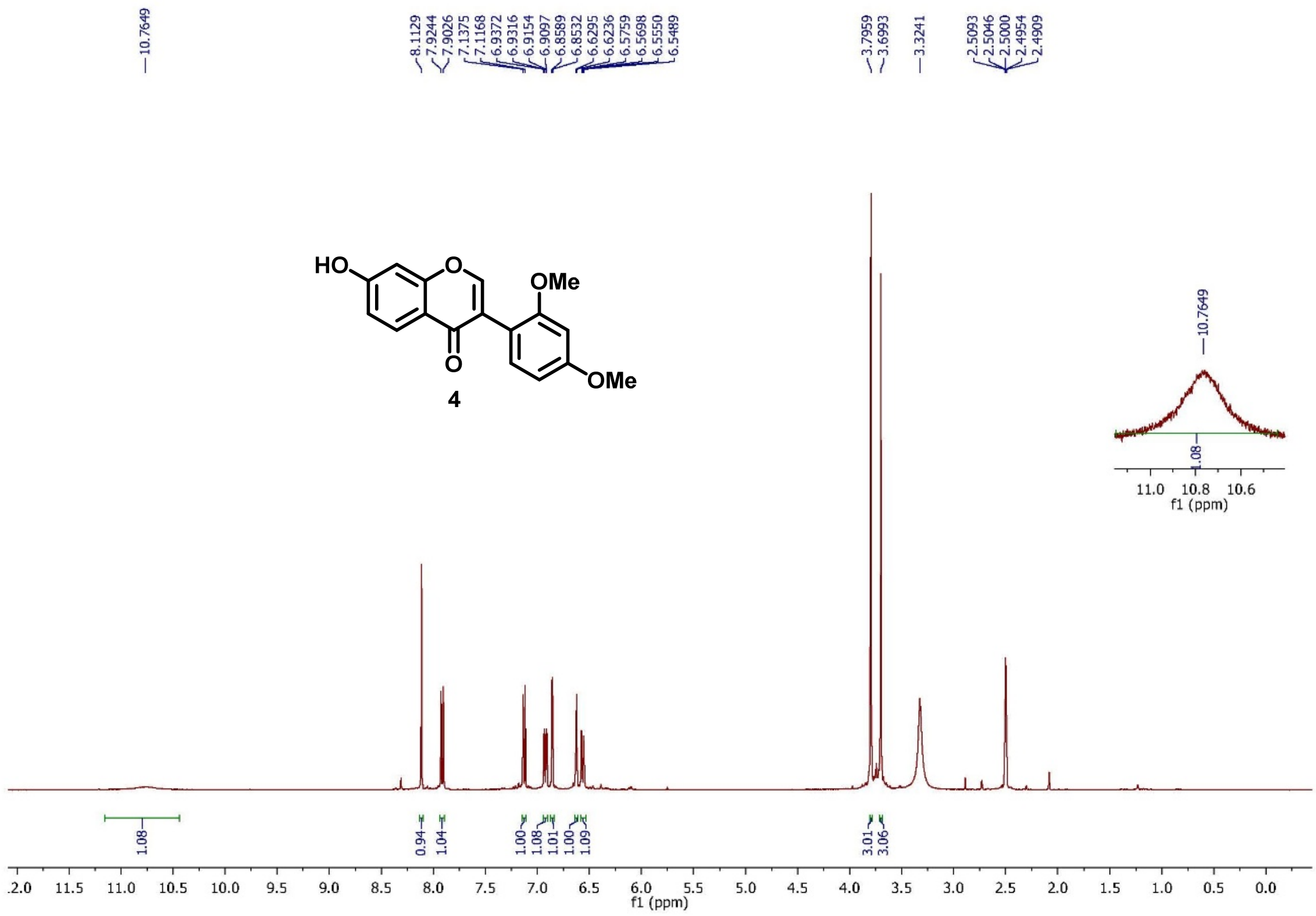

D: ^{13}C NMR of Compound-**4**

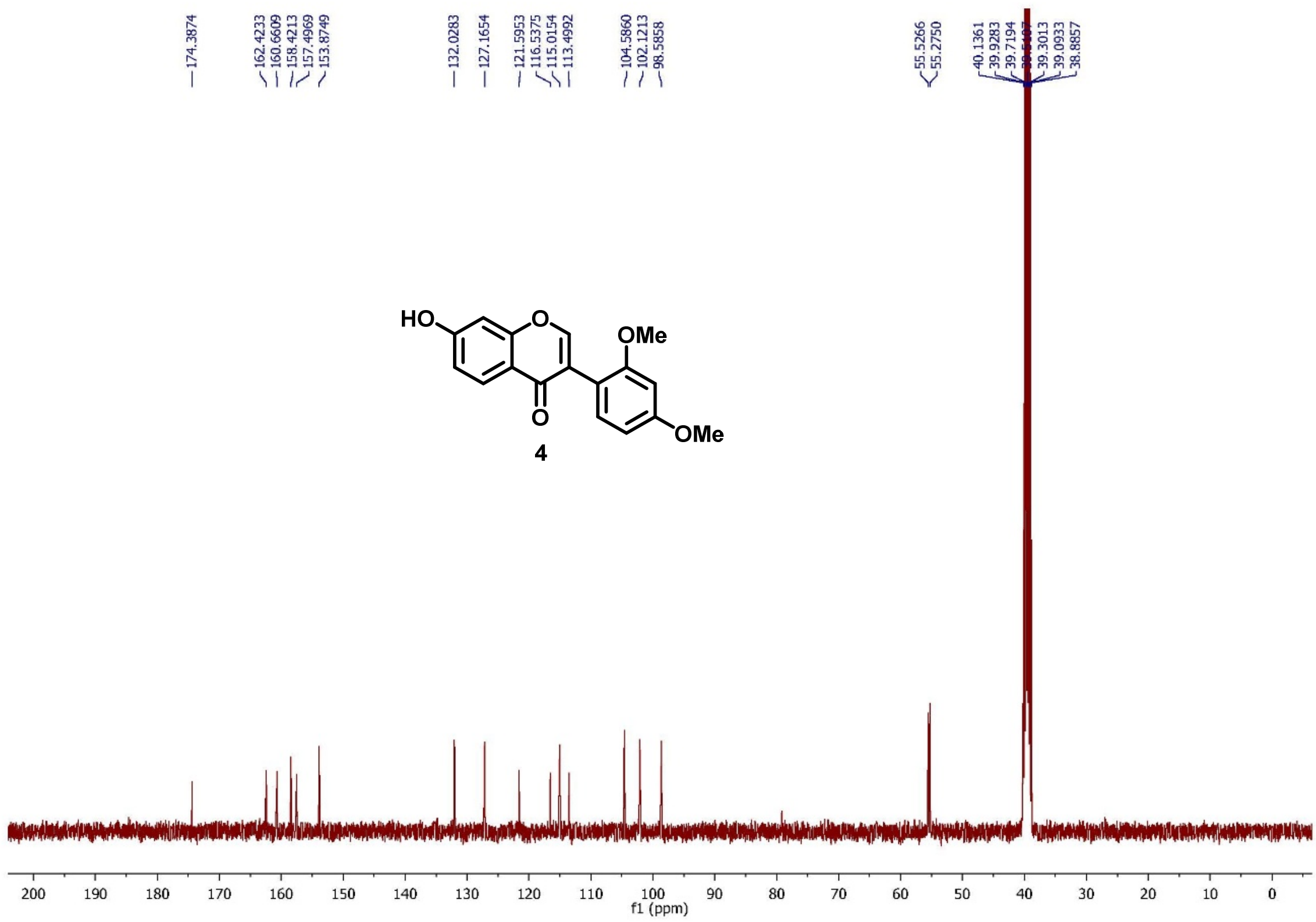

E: ^{1}H NMR of Compound-**5**

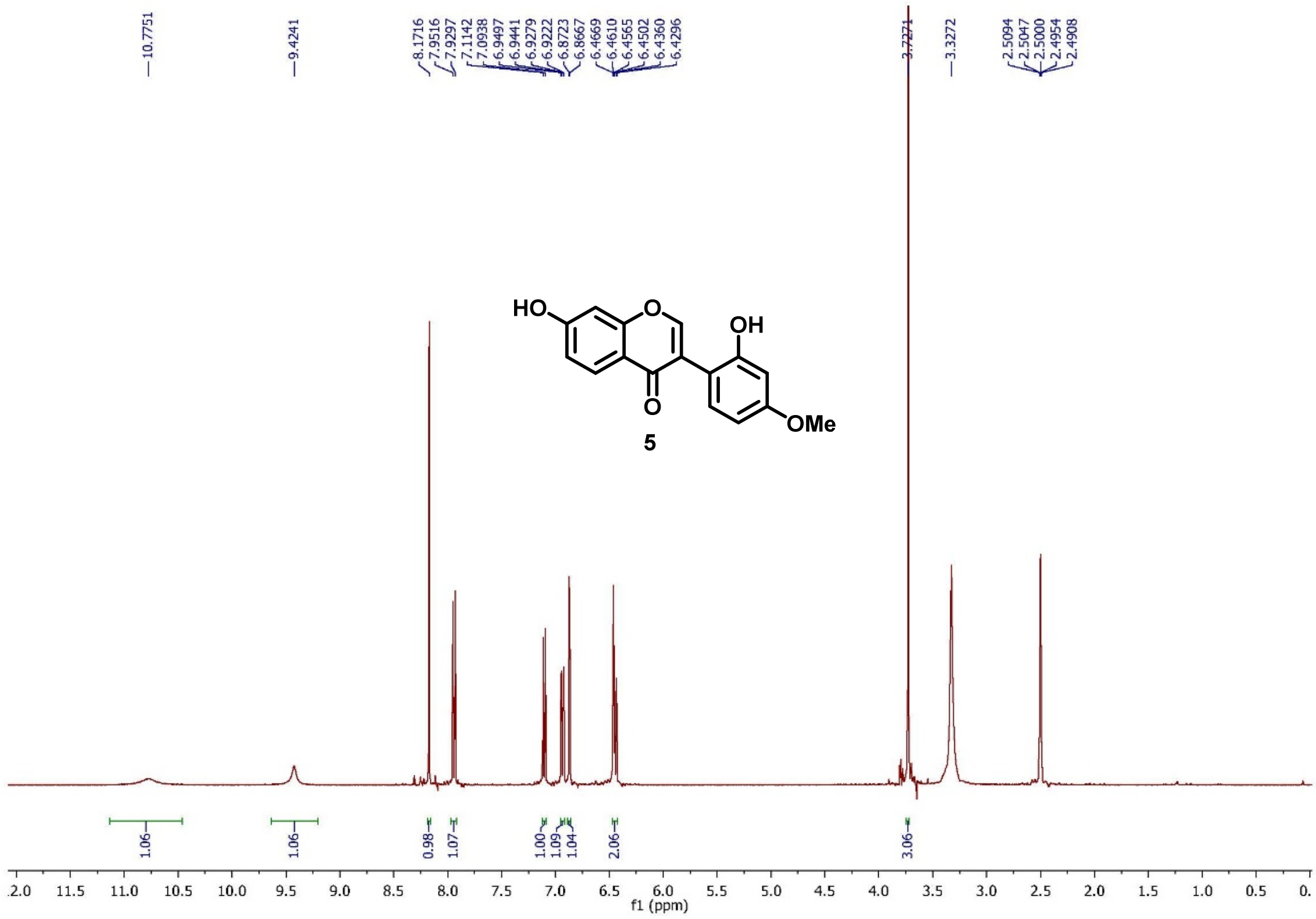

F: ^{13}C NMR of Compound-**5**

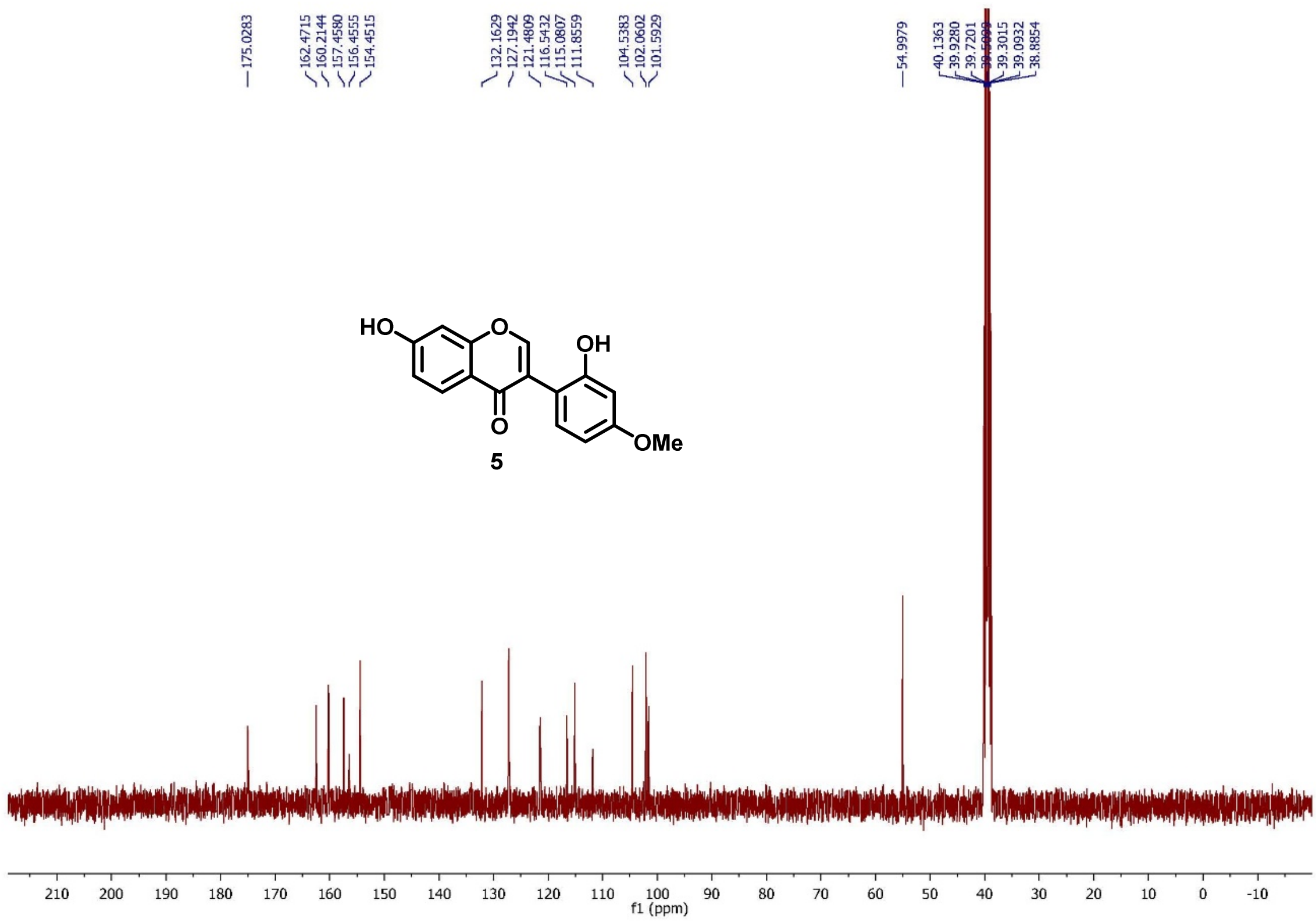

G: ^{1}H NMR of Compound-**6**

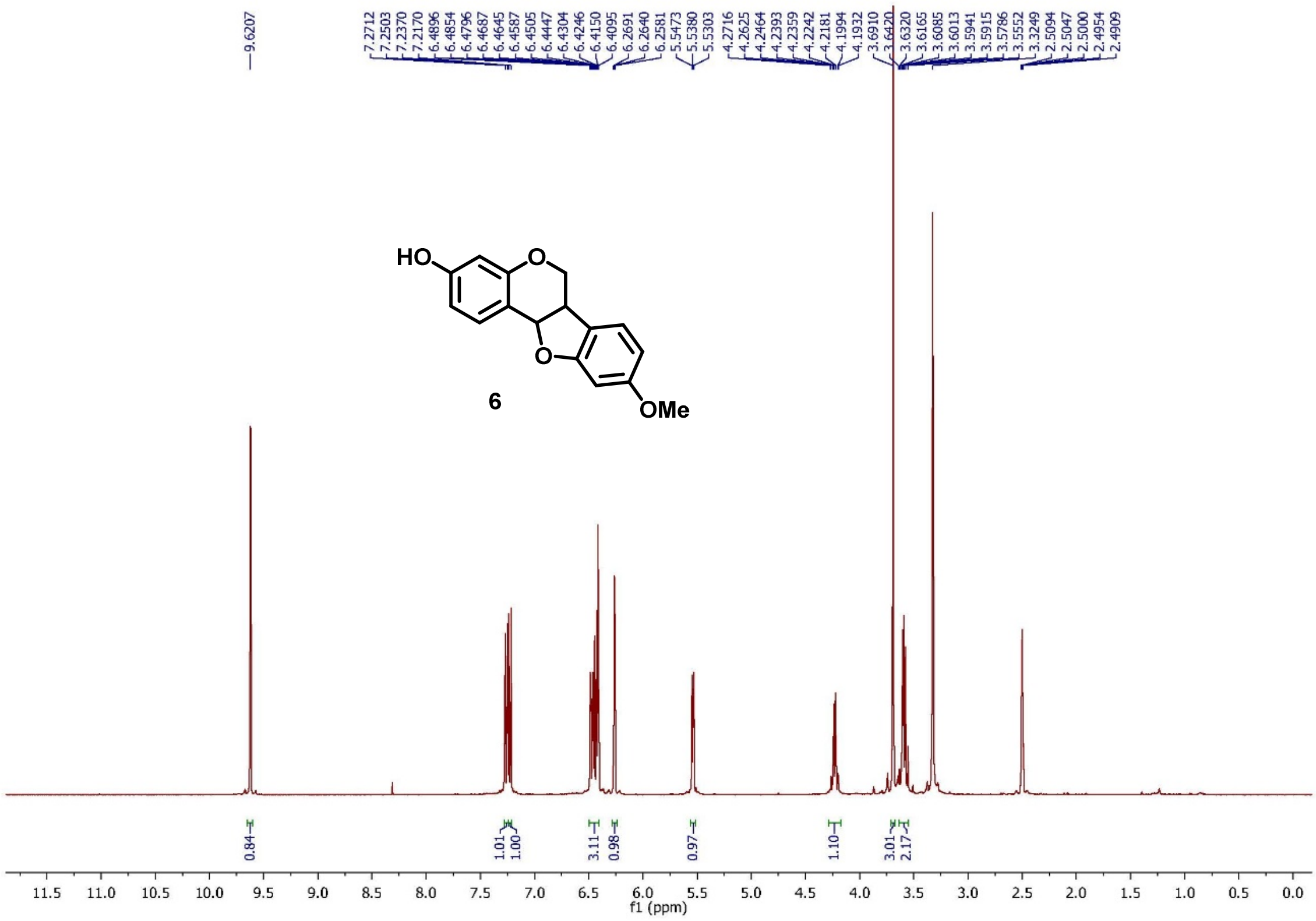

H: ^{13}C NMR of Compound-**6**

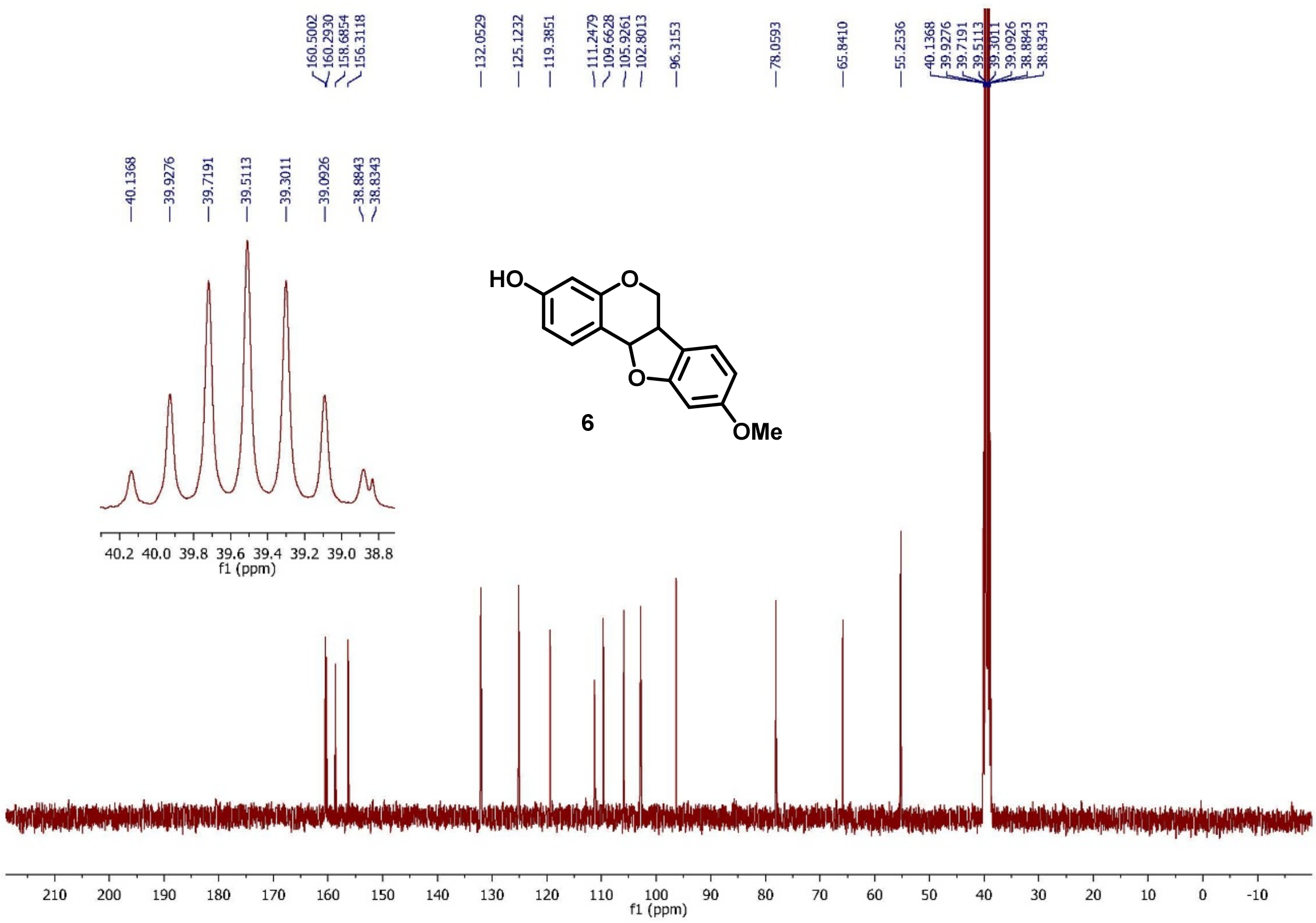

I: ^{1}H NMR of Compound-**7**

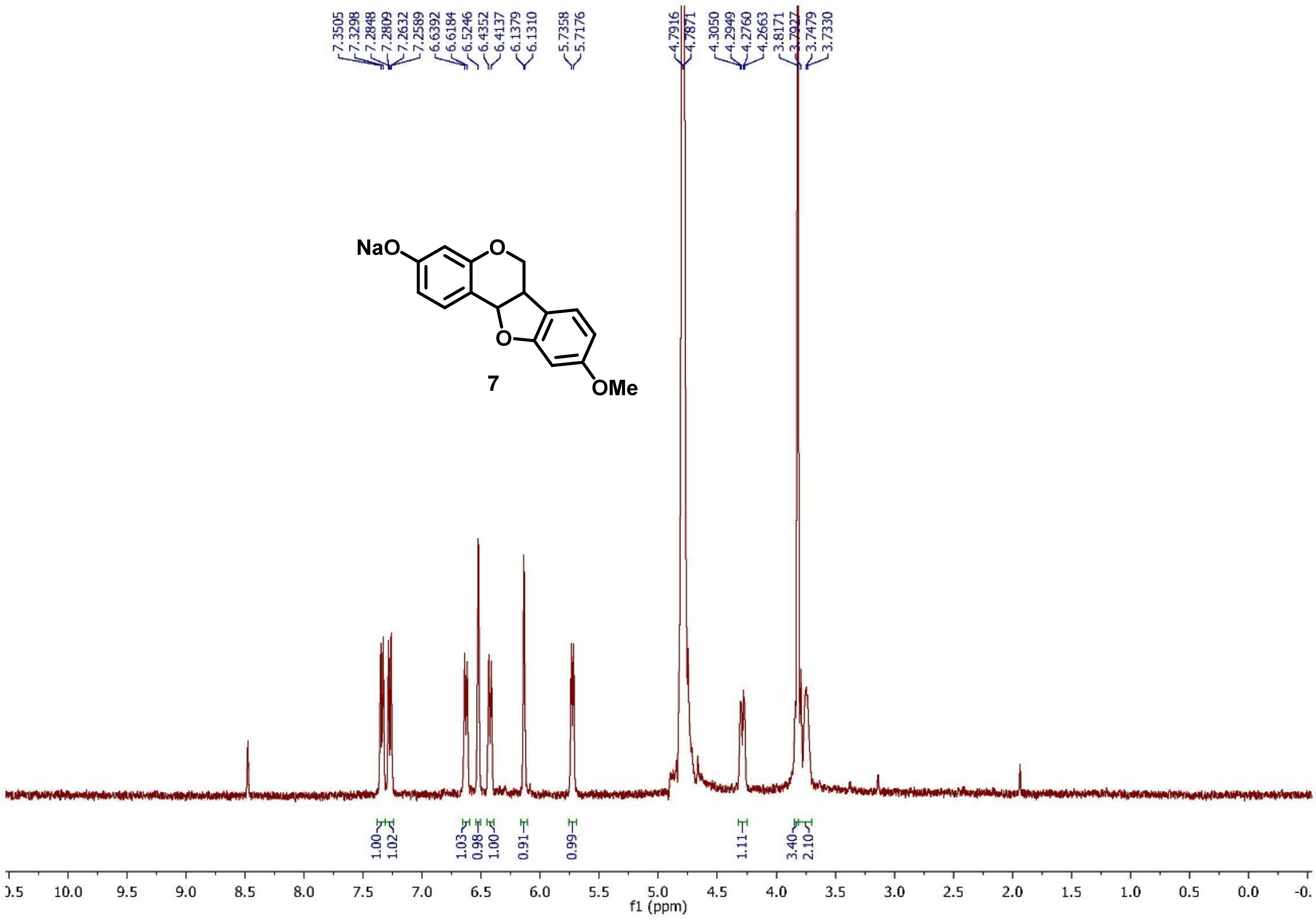

J: ^{13}C NMR of Compound-**7**

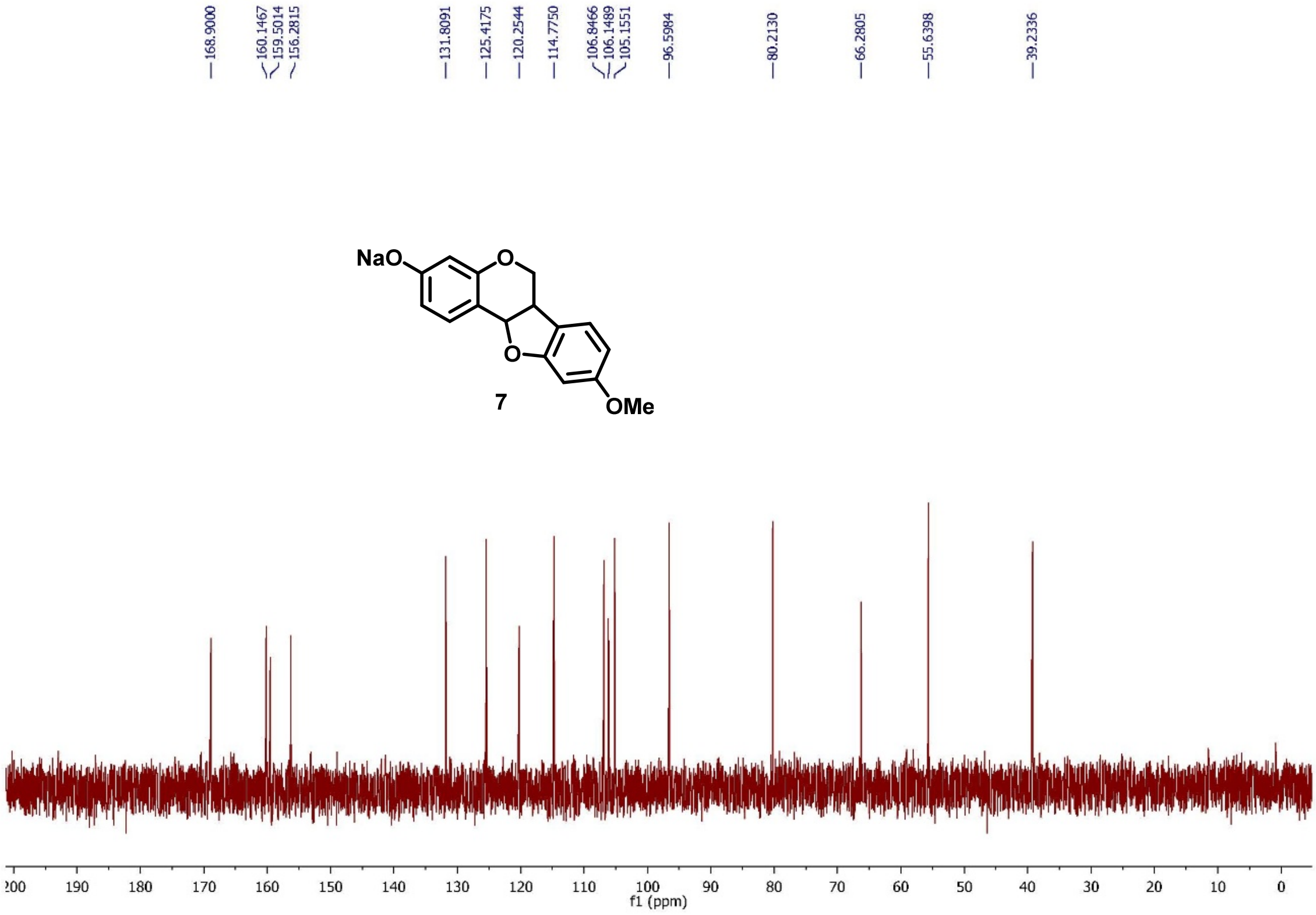

Supplementary Full Blots

Figure-1C full blots

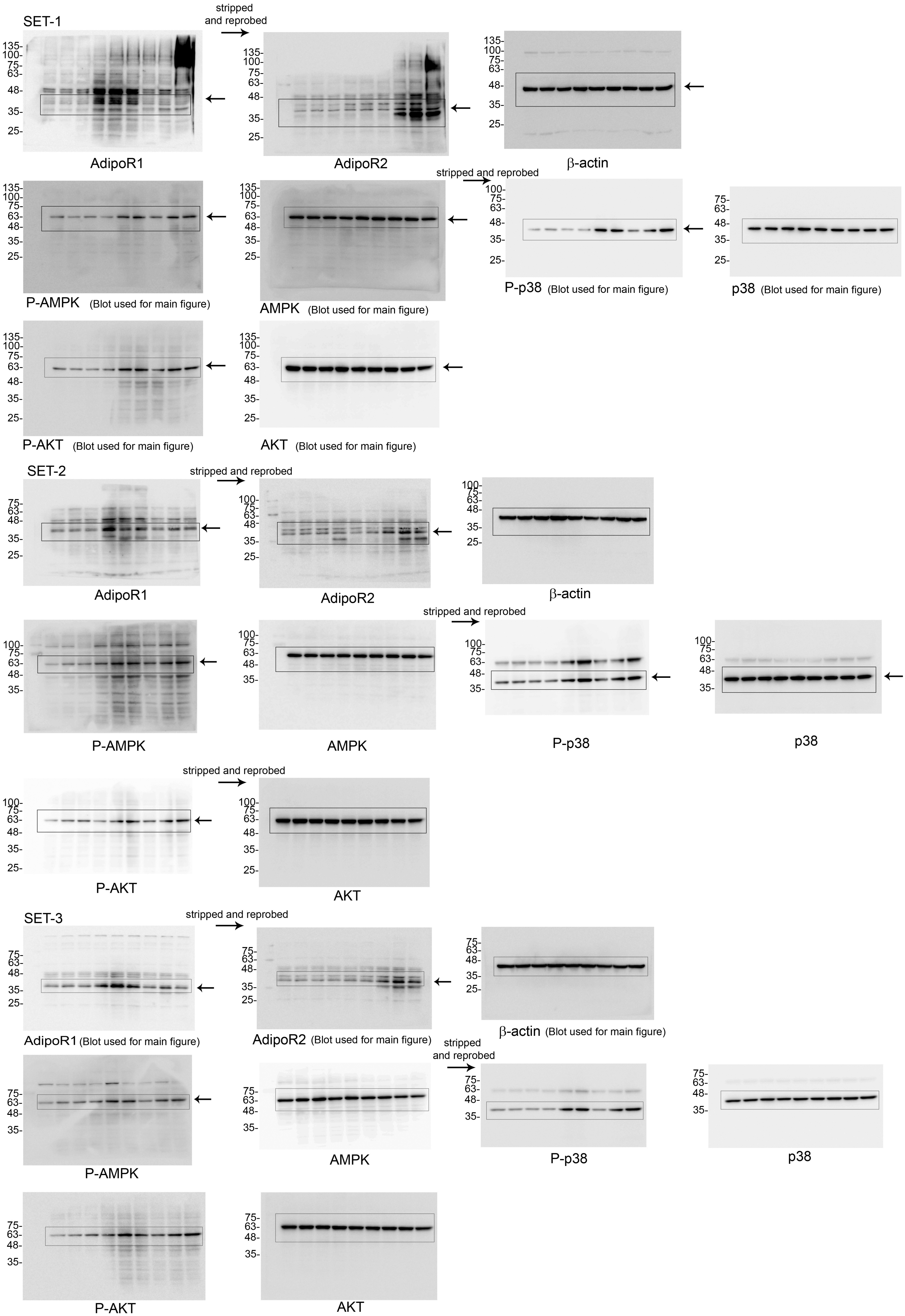

Figure-1D full blots

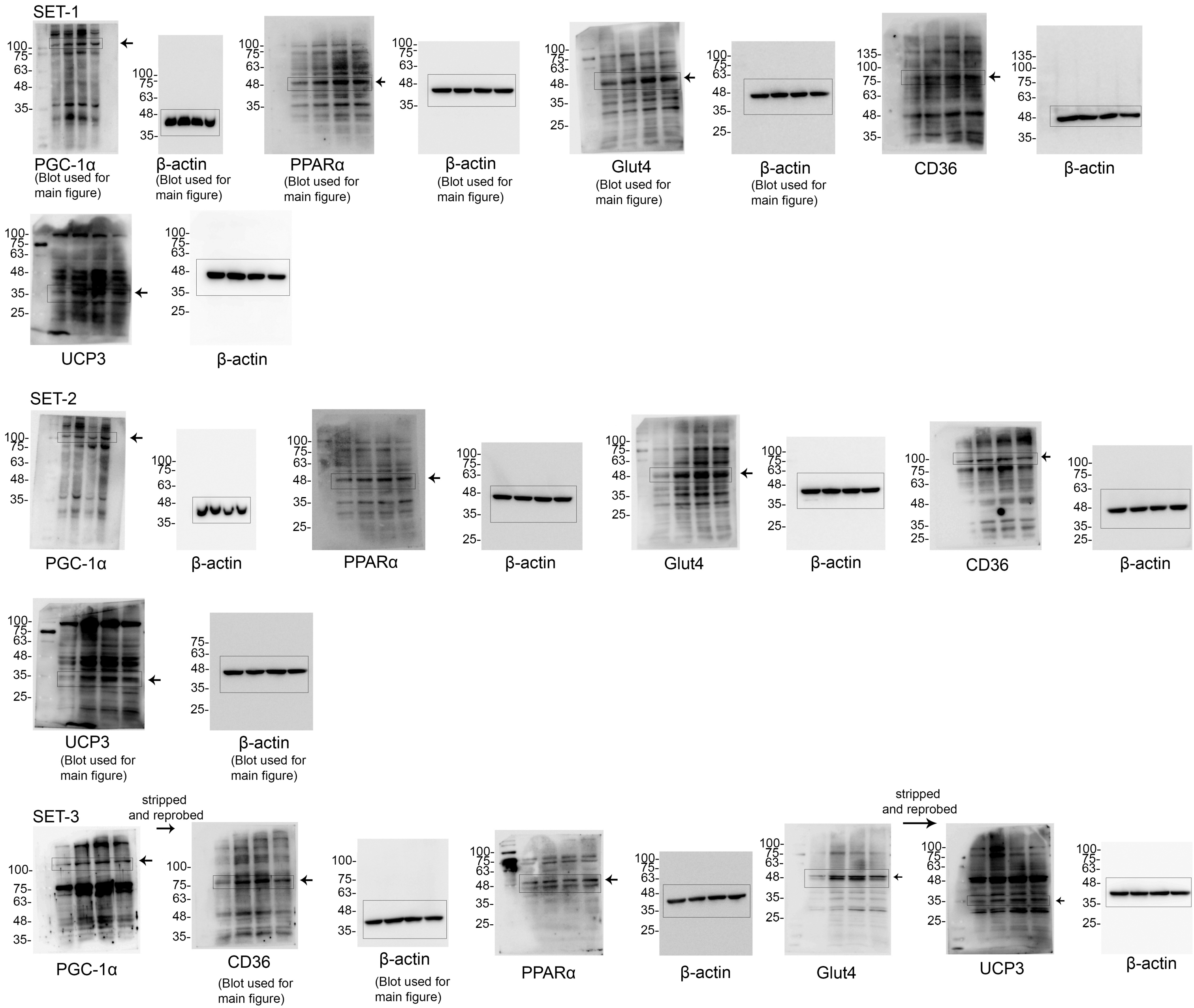

Figure-1E shRNA set-1

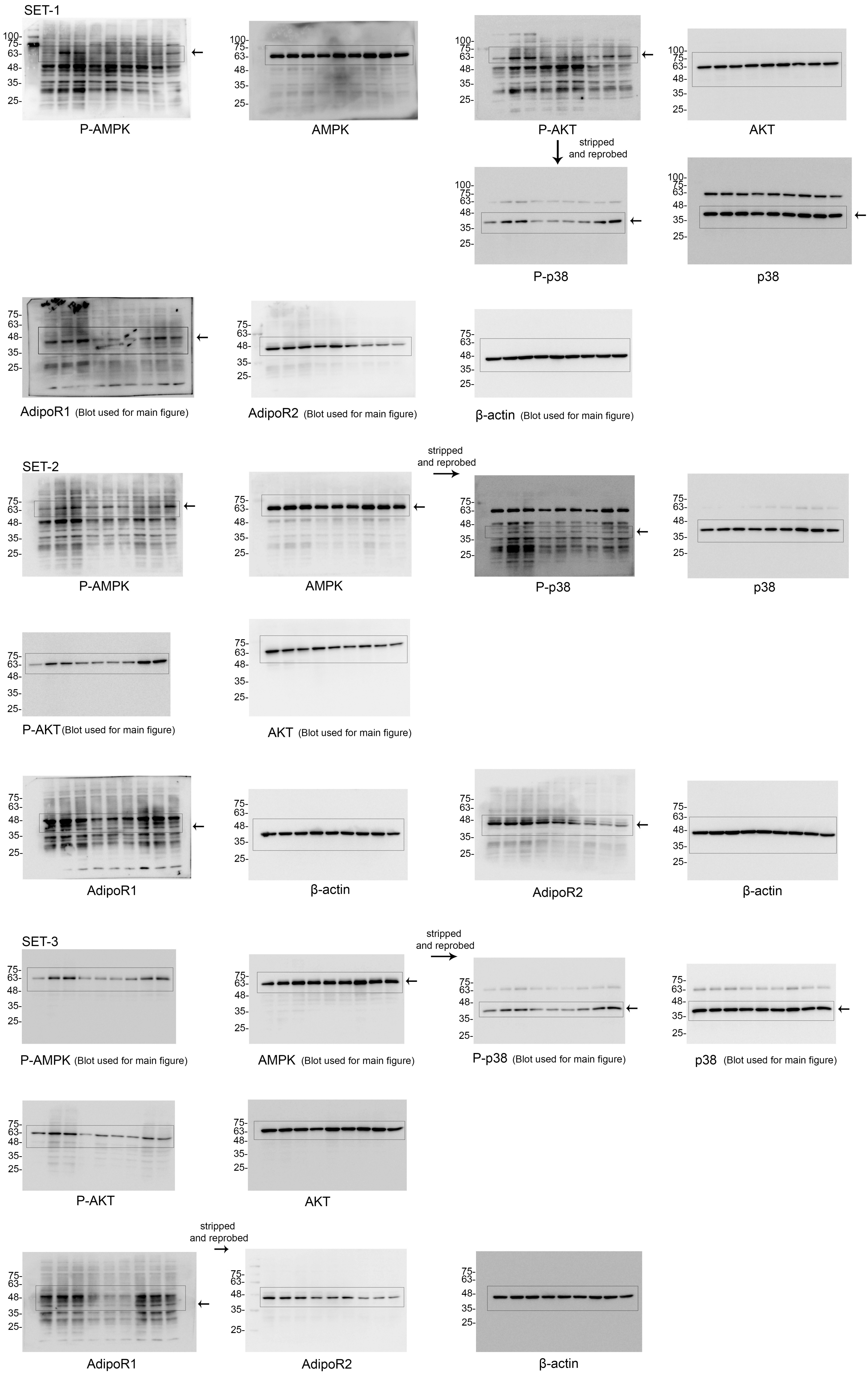

Figure-1F shRNA set-1

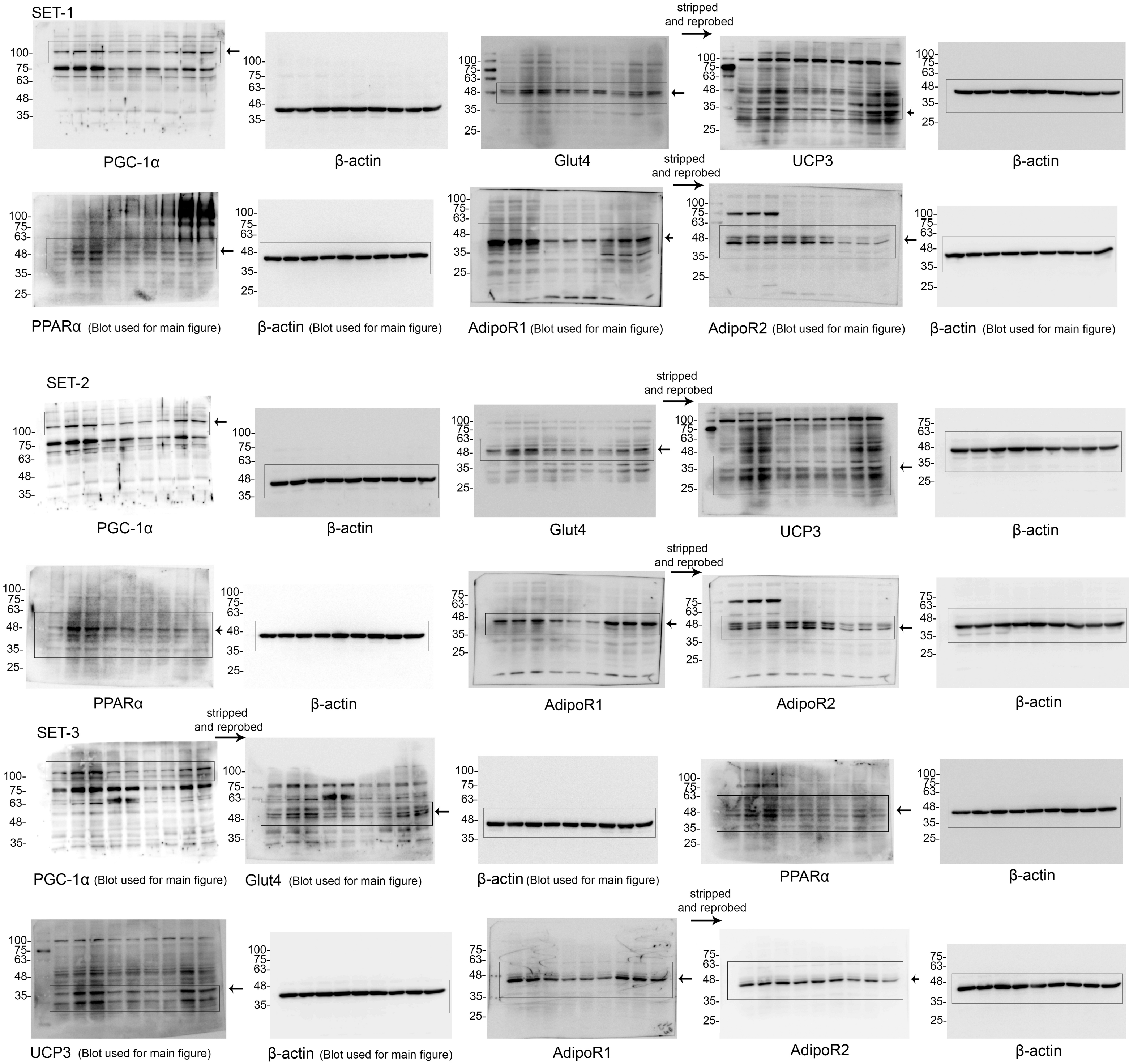

Figure 2- Full blots

Figure 2B- Full blots

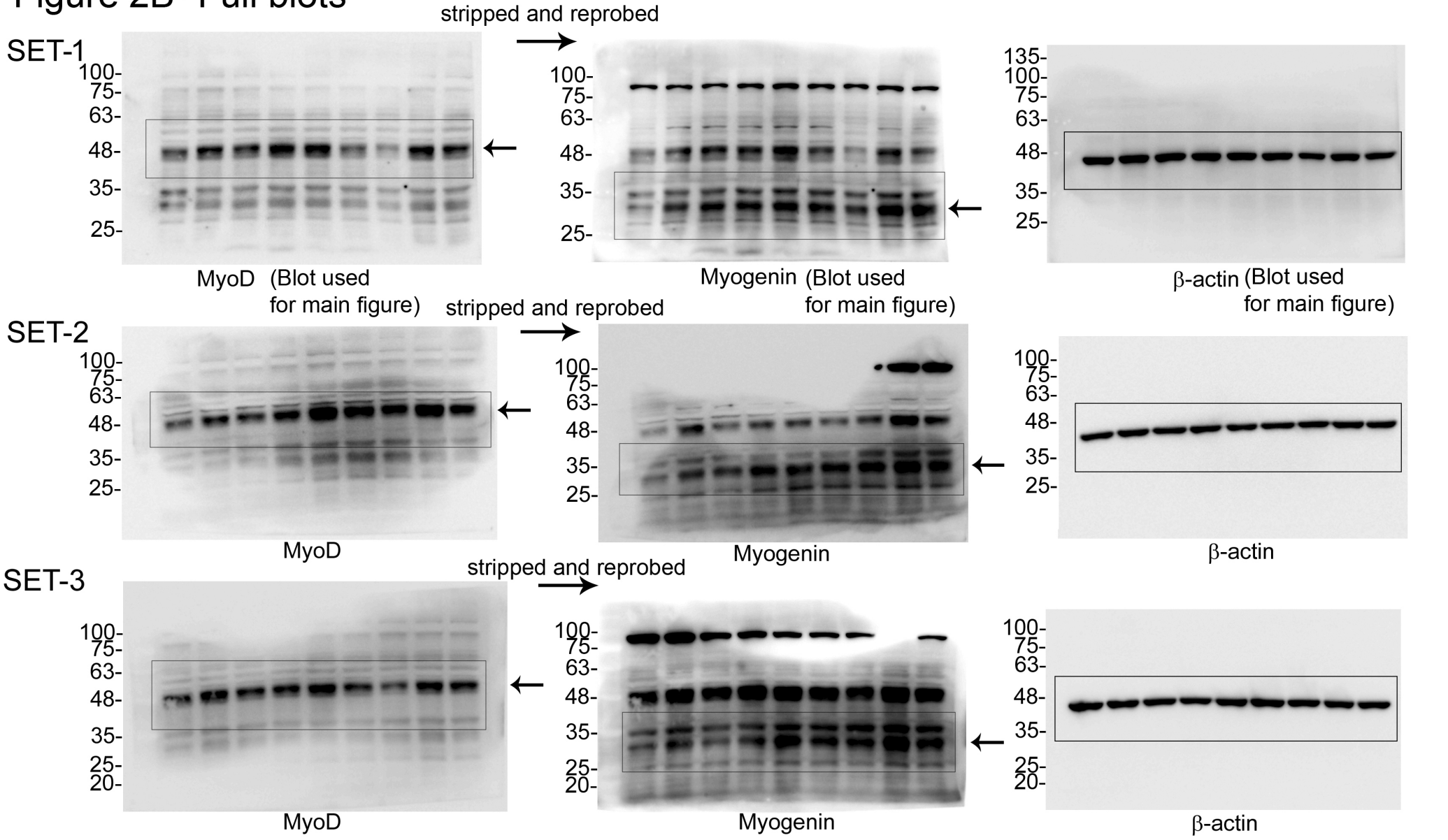

Figure 2D- Full blots

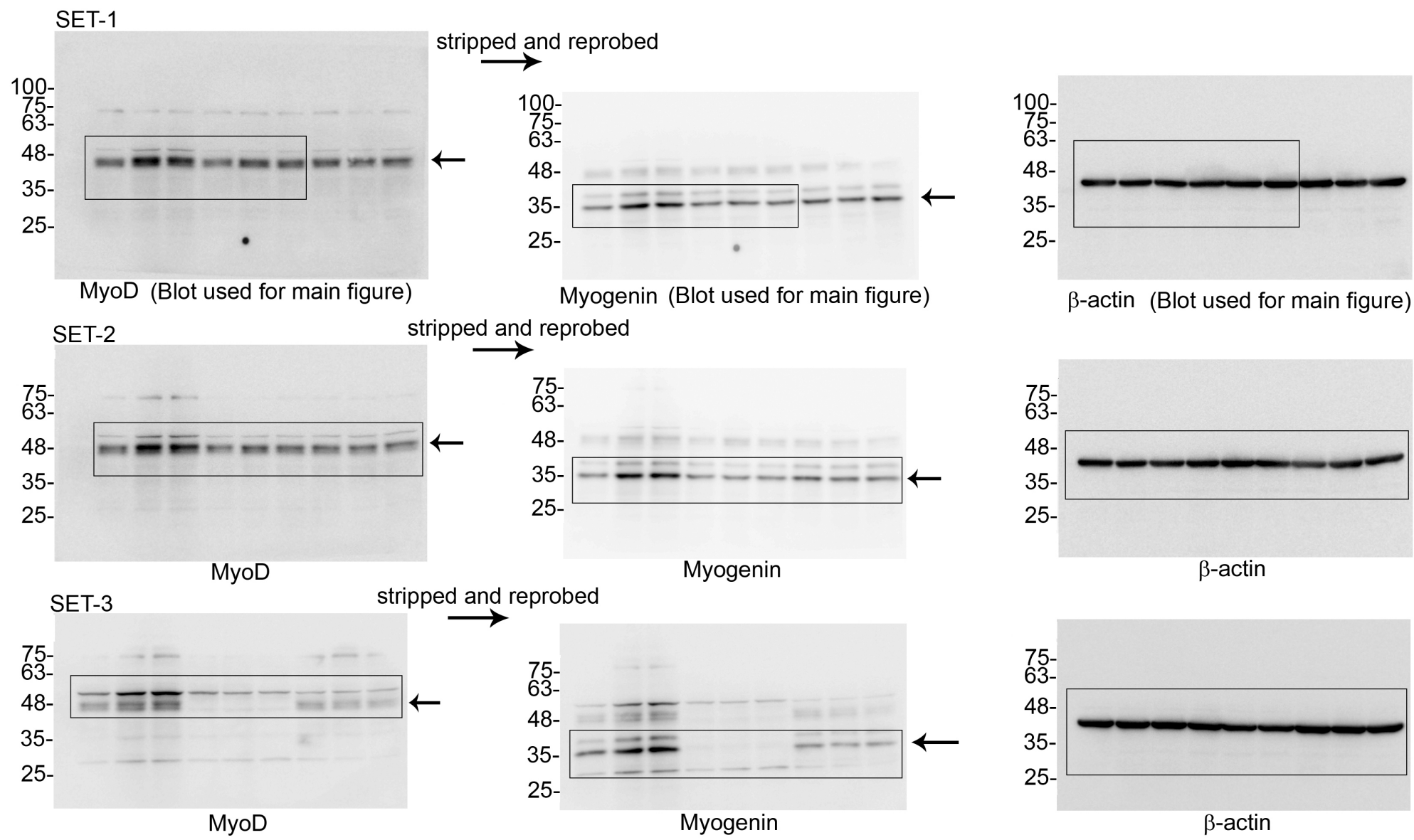

Figure-3

Figure 3B full blots

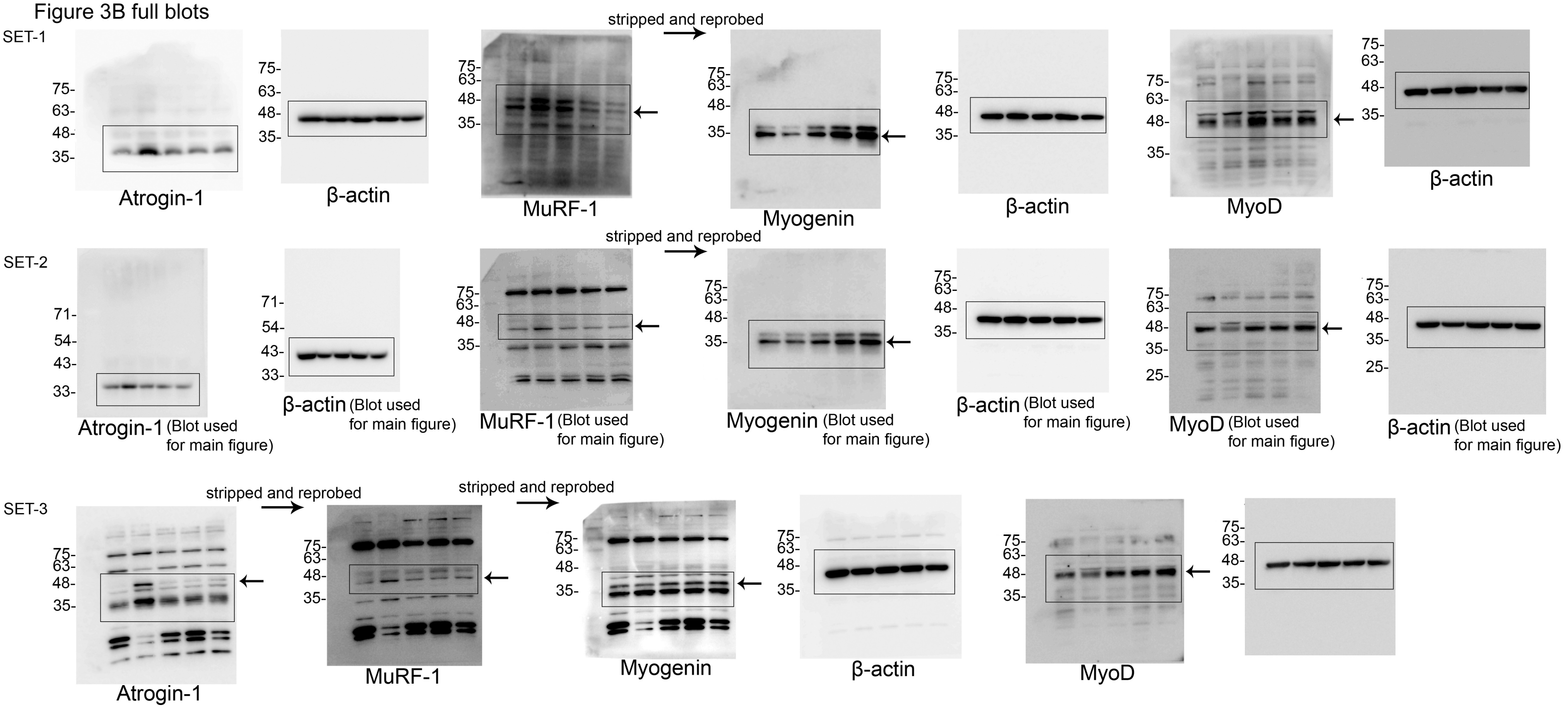

Figure 3D full blots

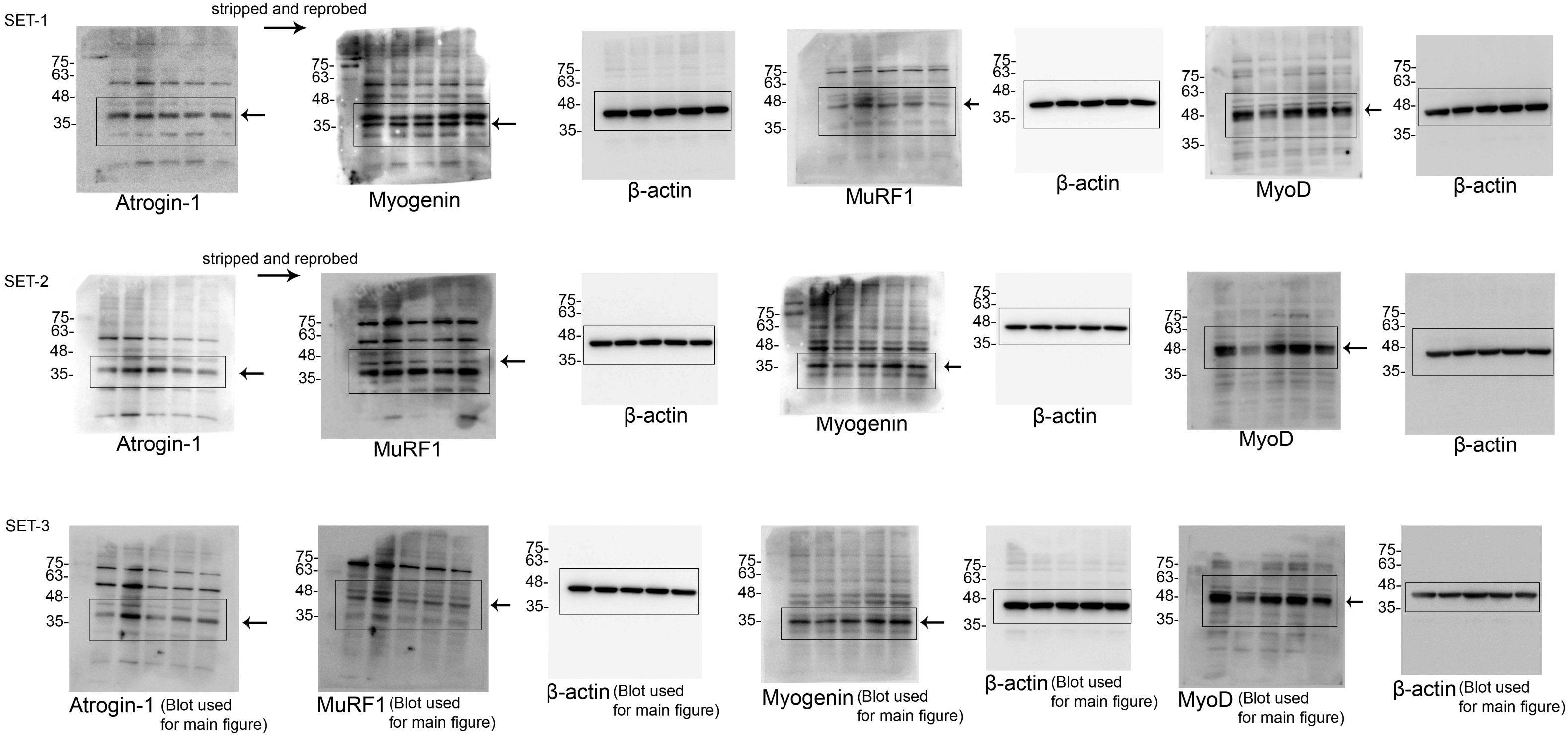

Figure 3

Figure 3F: PBS (+Ca2+) full blots

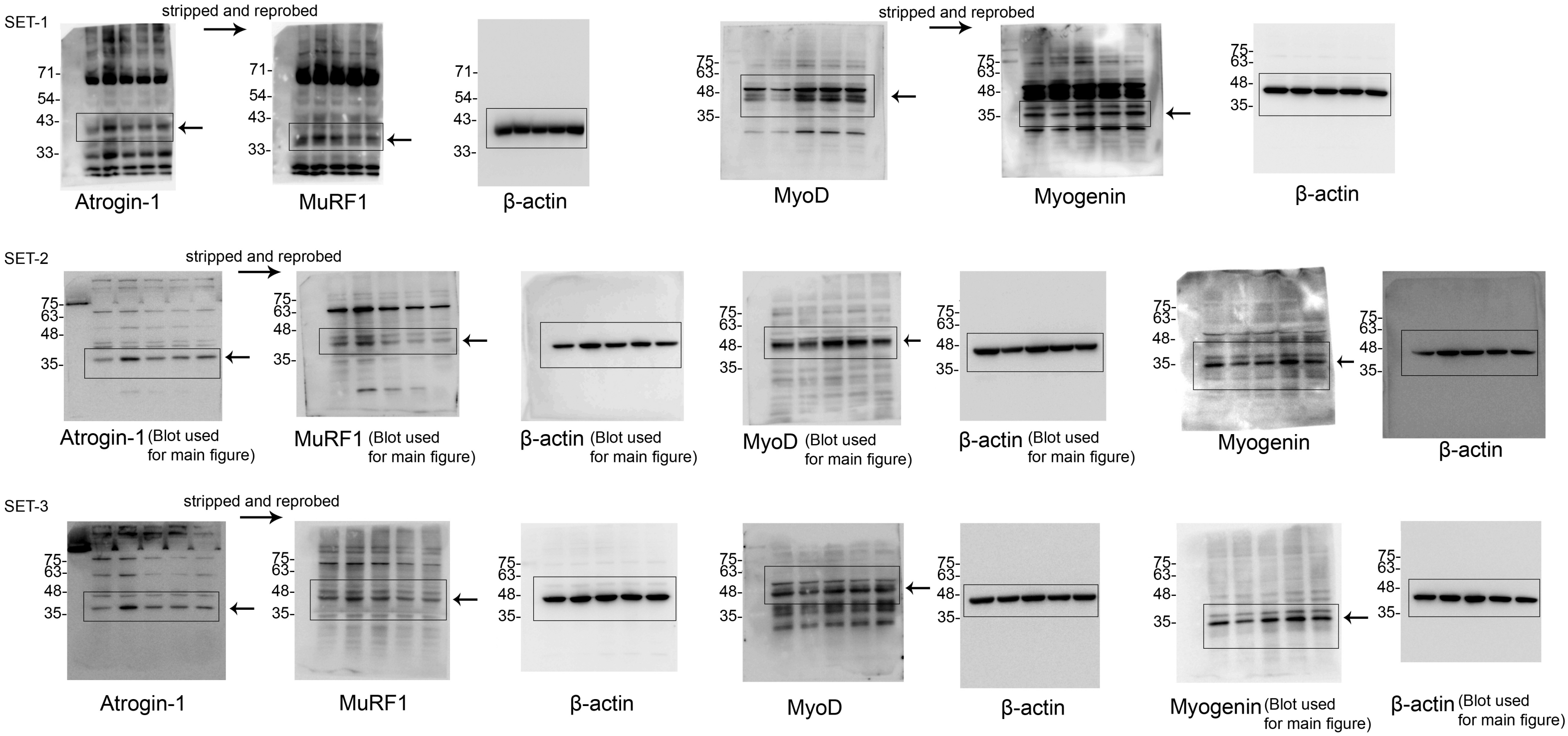

Figure 3F: PBS (-Ca2+) full blots

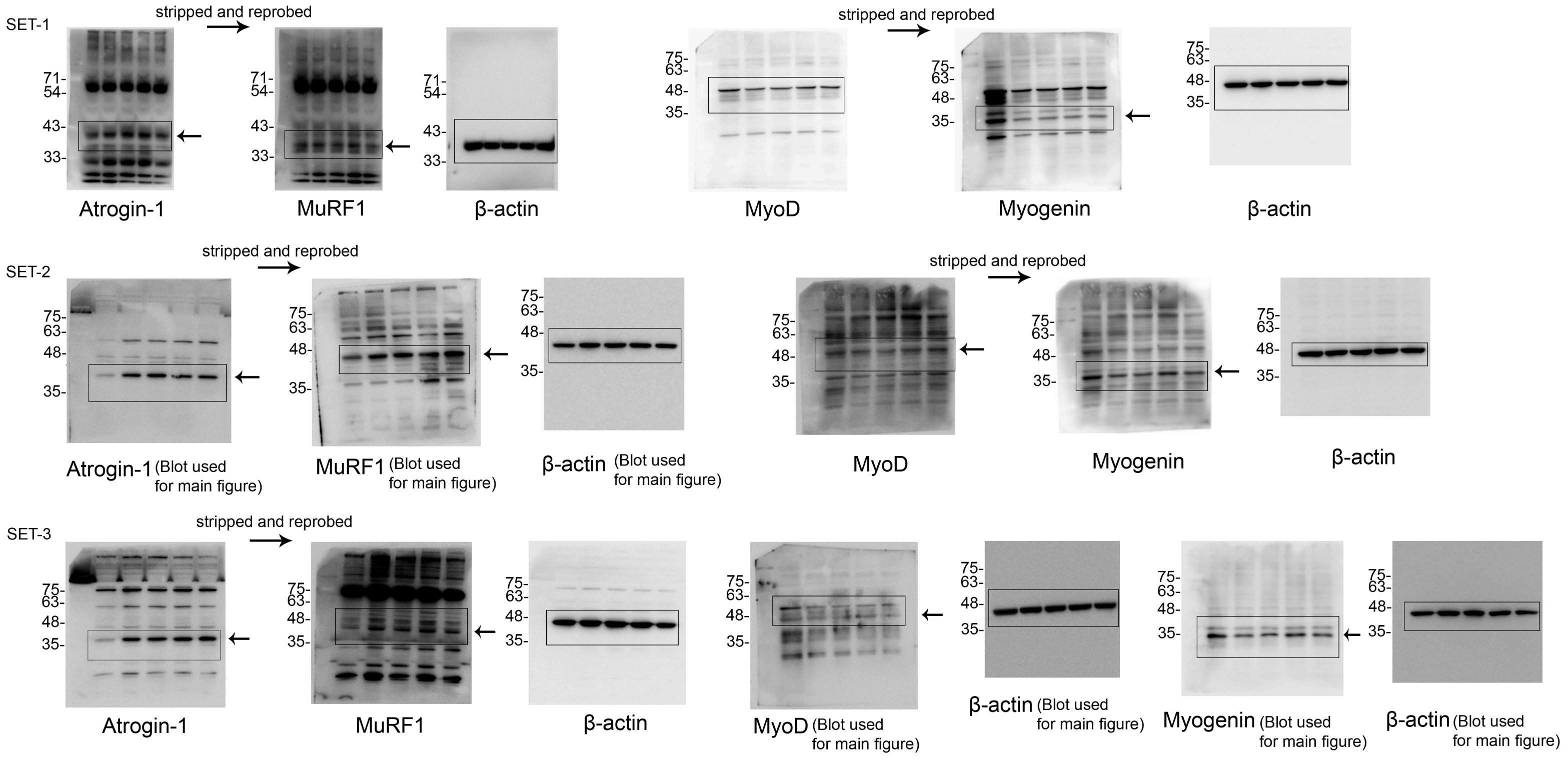

Figure 4

Figure-4A full blots.

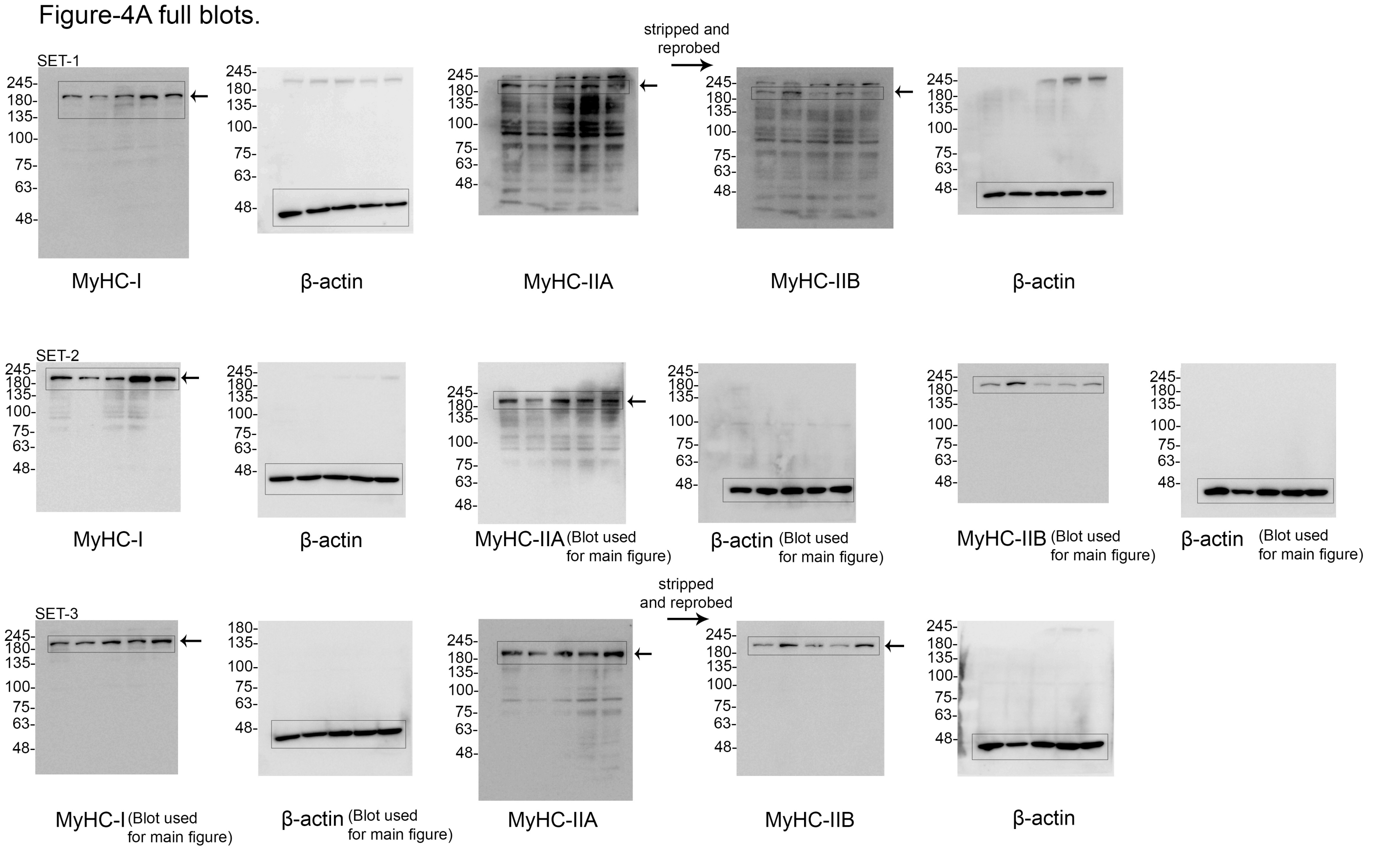

Figure-4D full blots

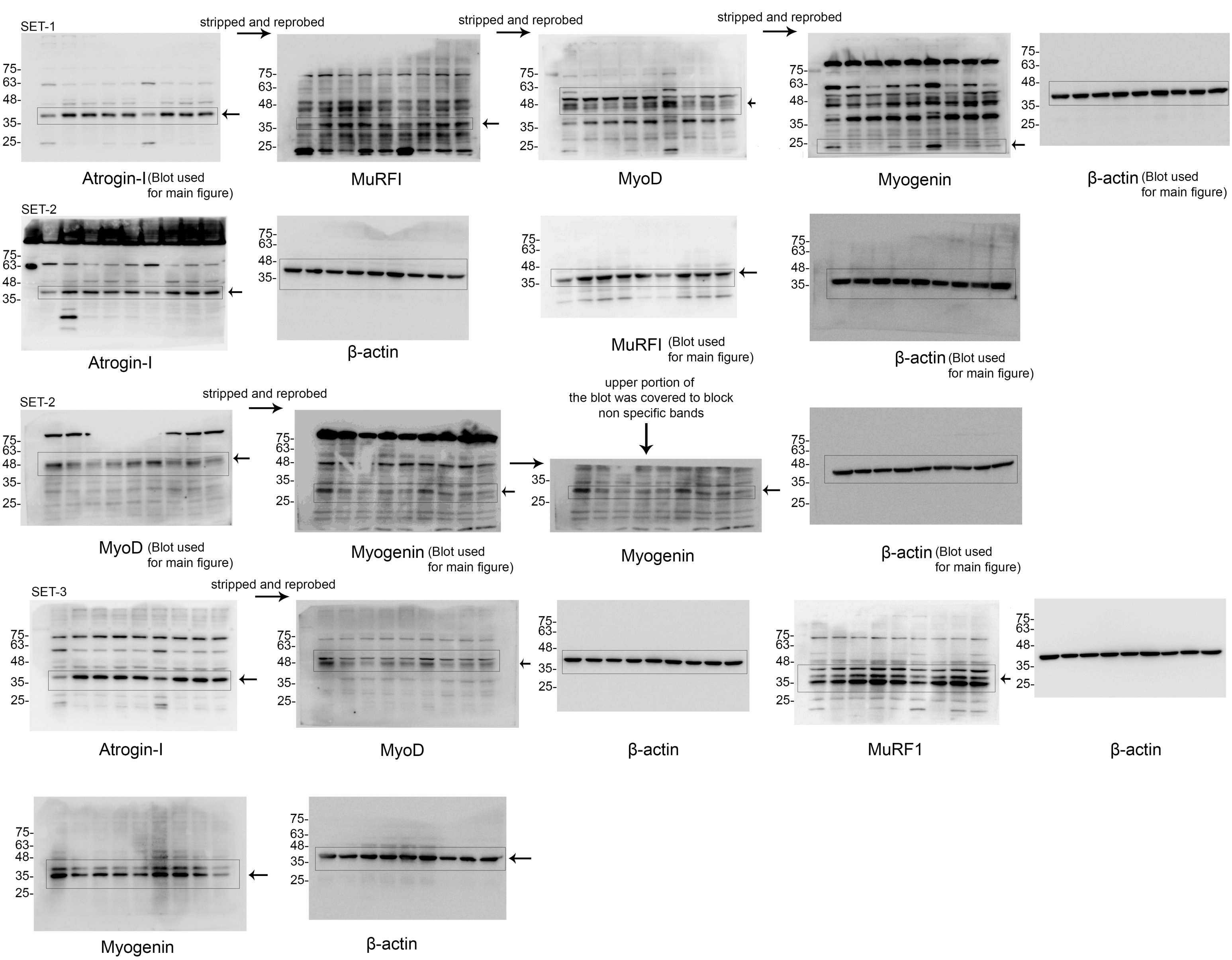

Figure 5C

Part A

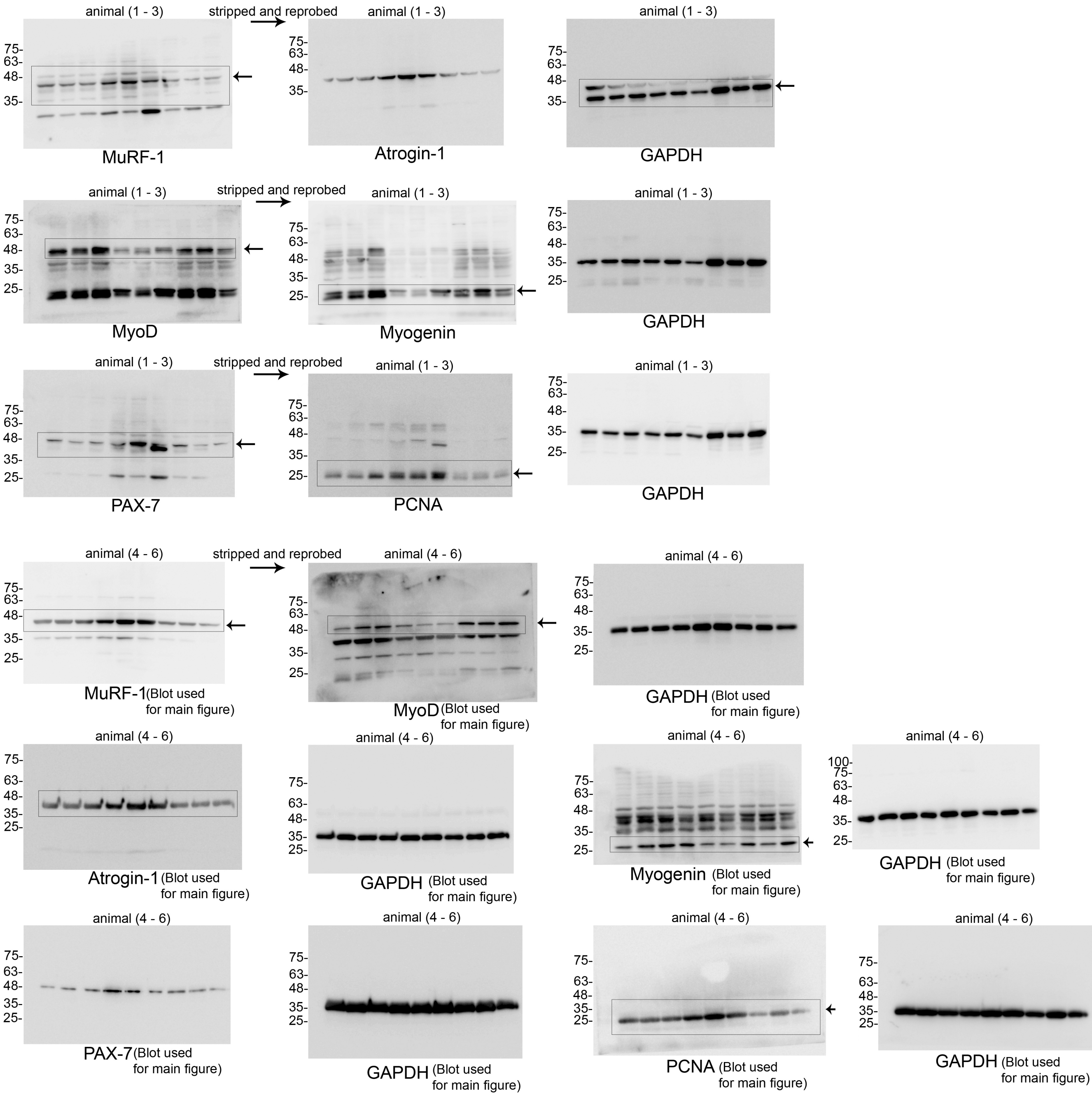

Figure 5C
Part B

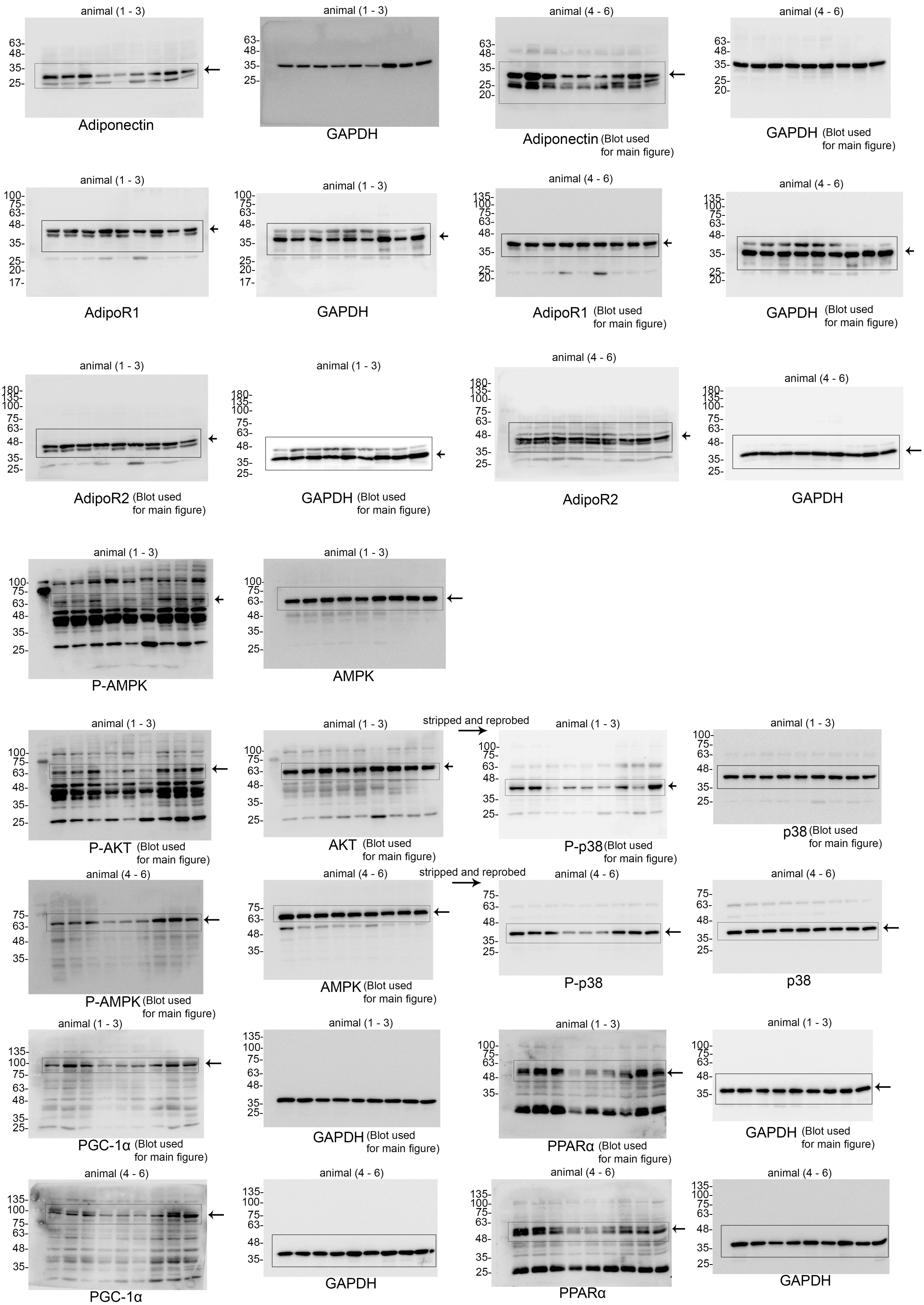

Figure 5C

Part C

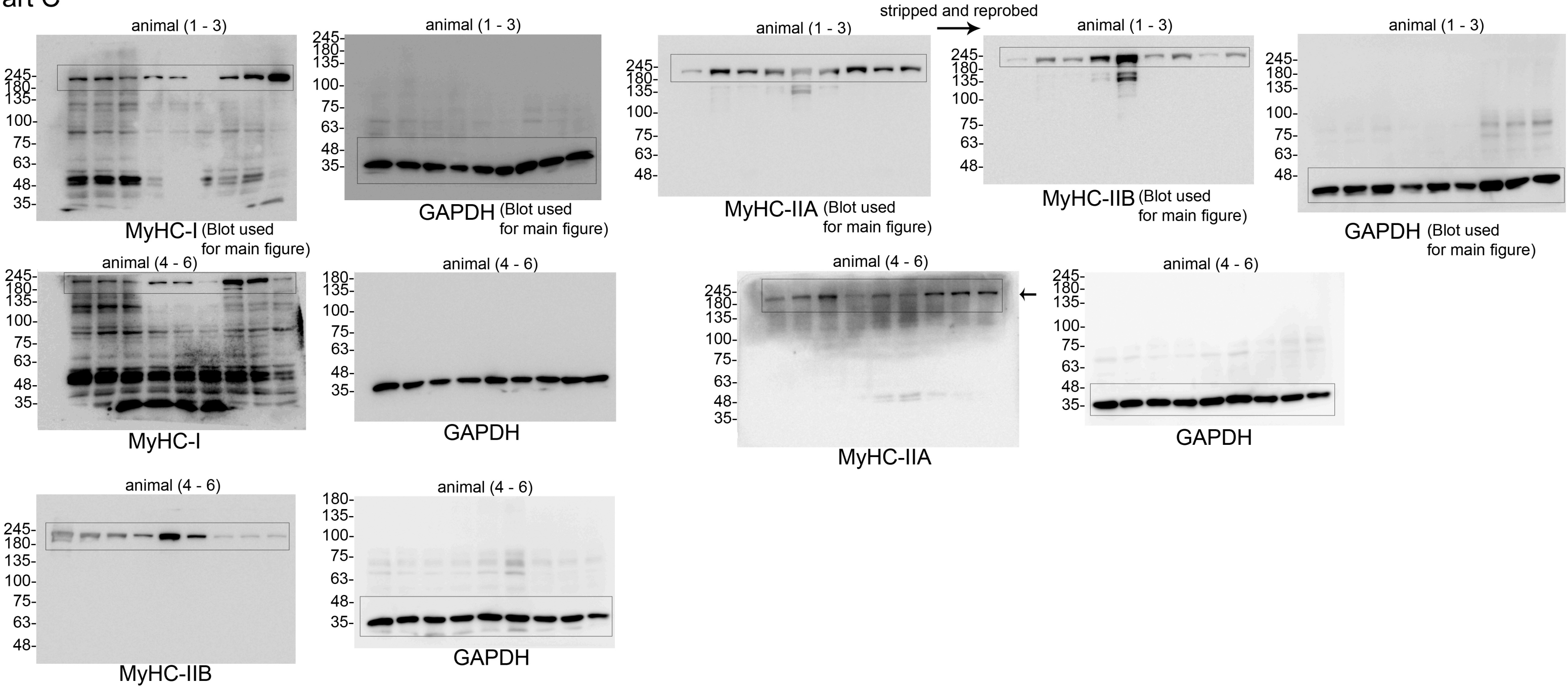

Figure-6D full blots (3 day)

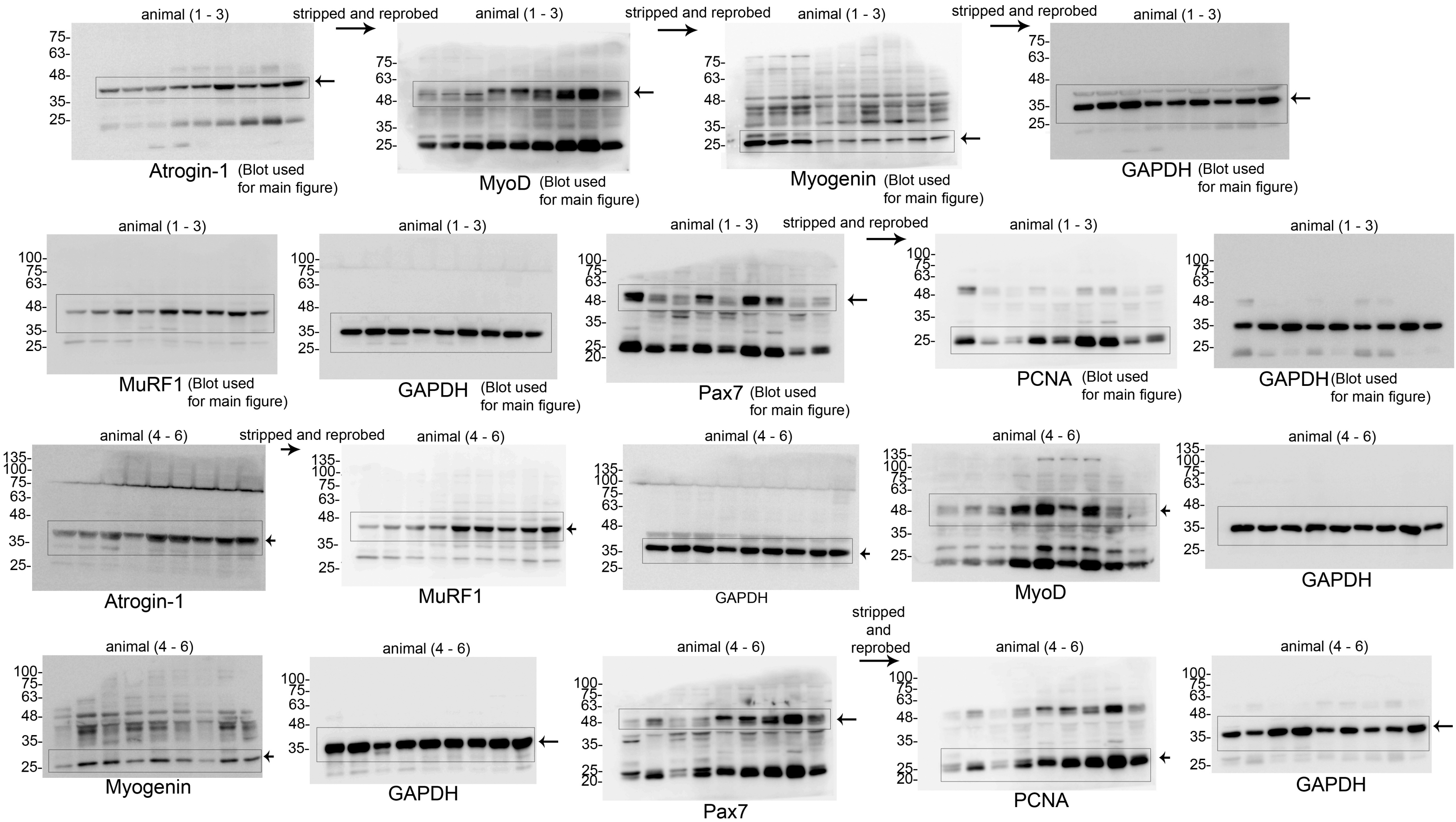

Figure-6E full blots (7 day)

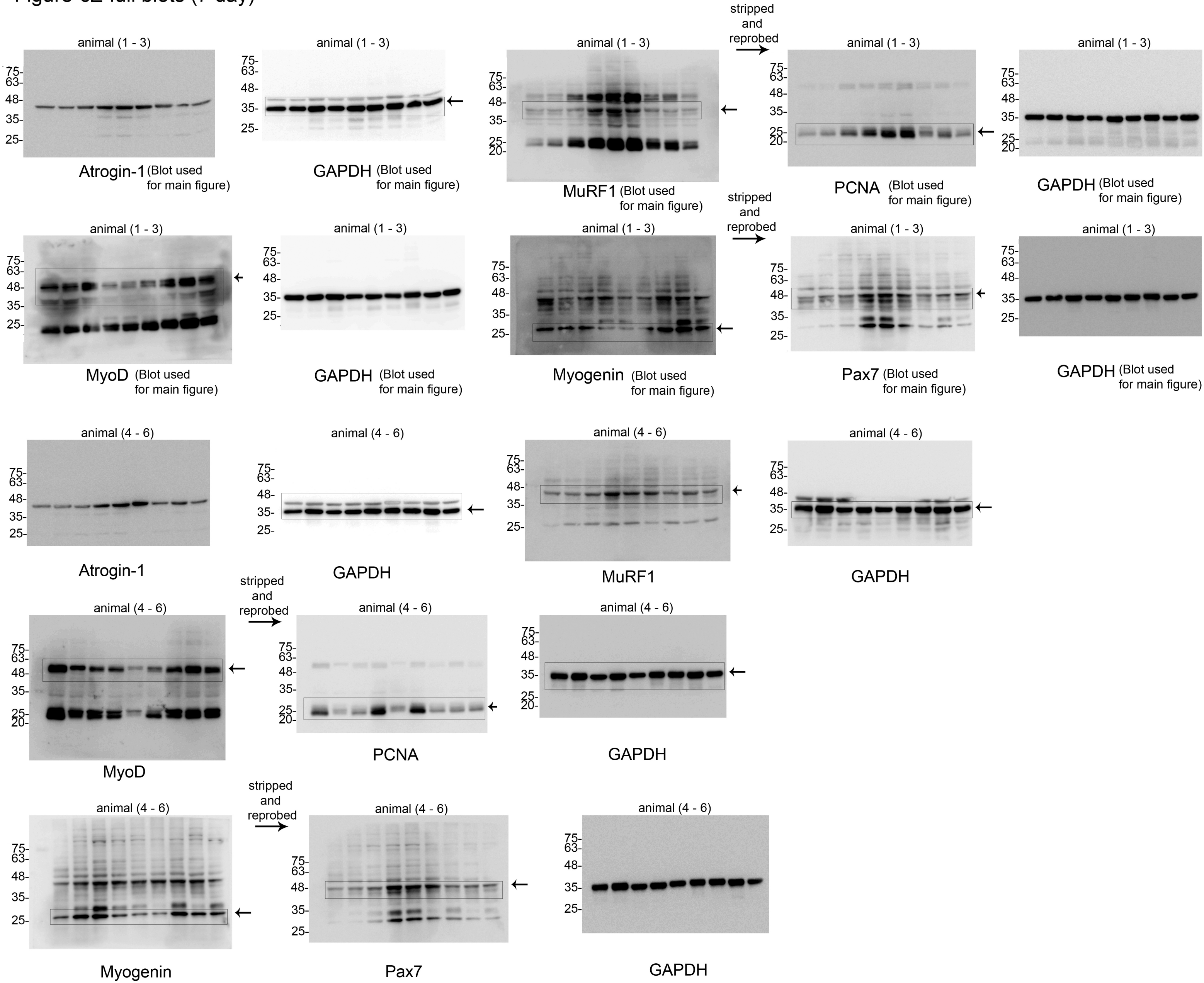

Figure-6E full blots (7 day)

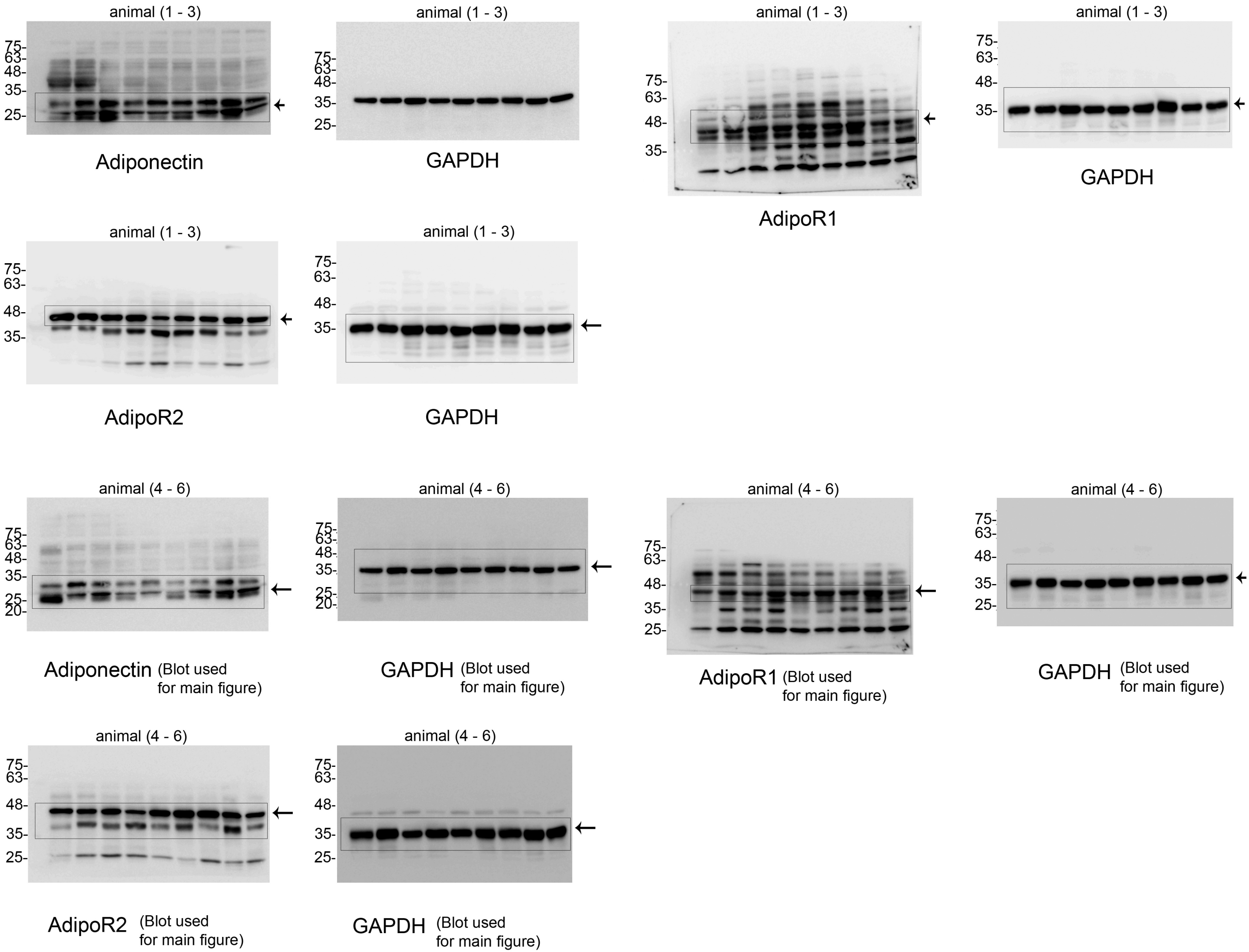

Supplementary Figure S1: full blots

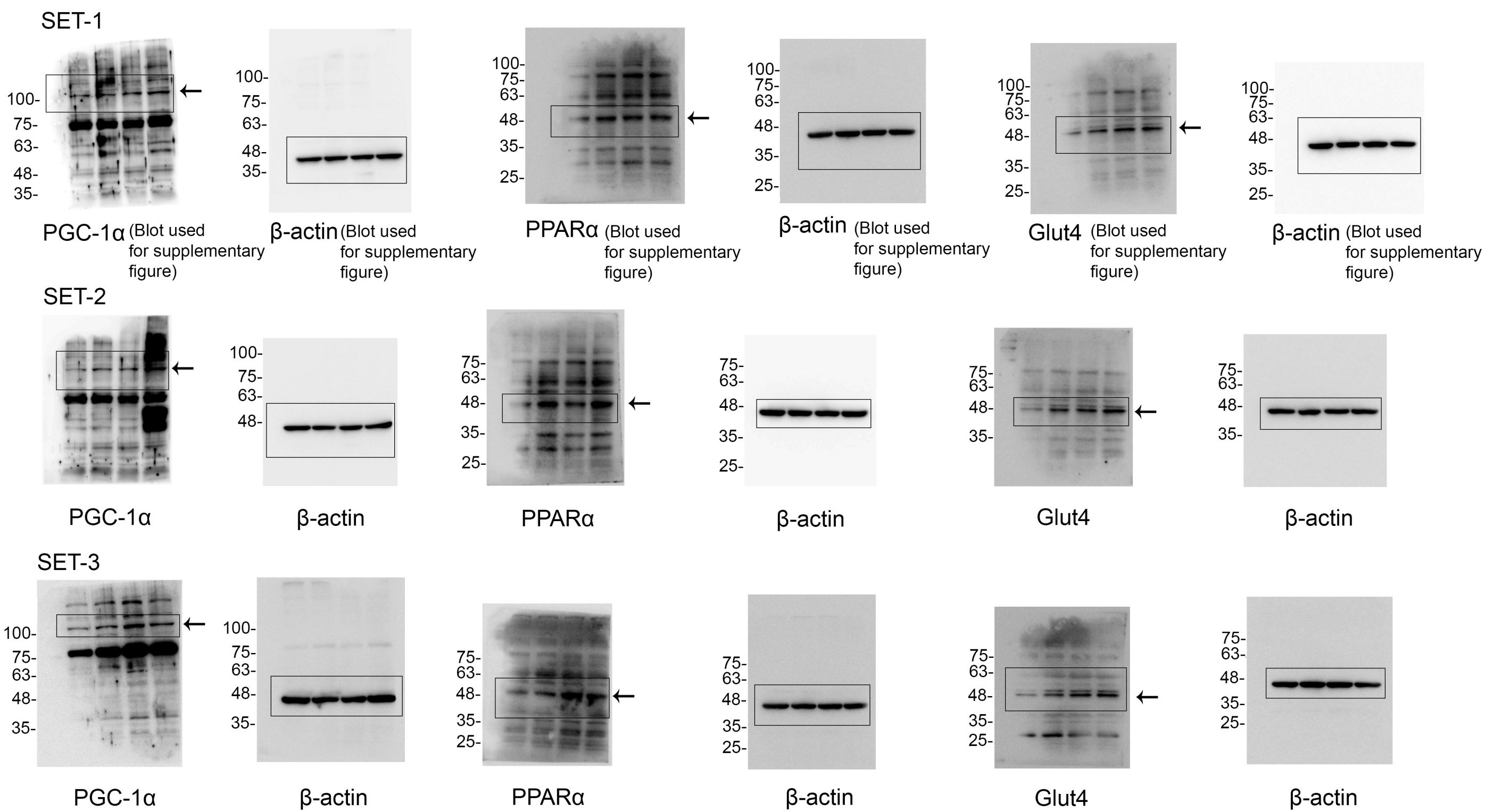

Supplementary Figure S2

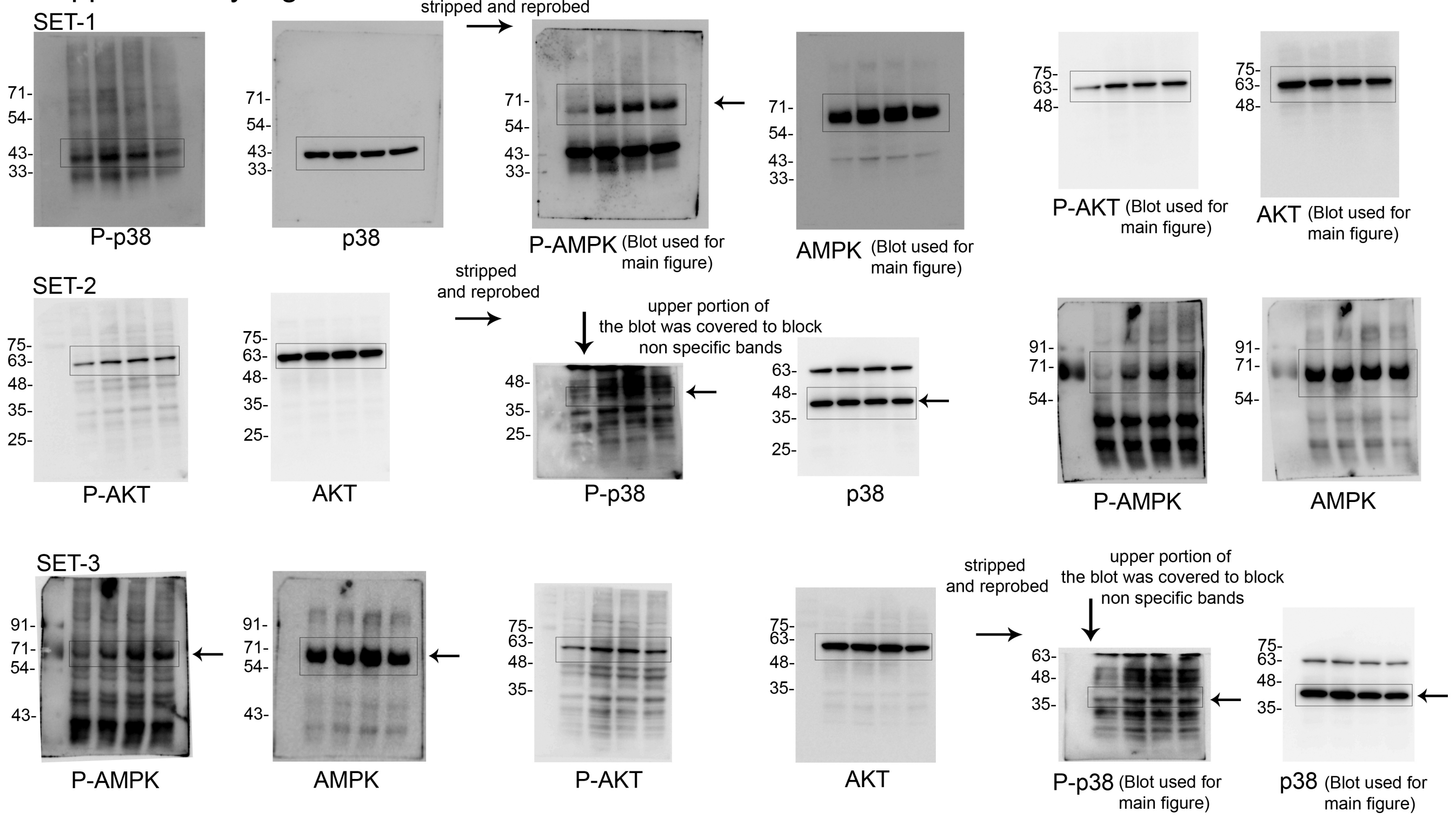

Supplementary Figure S4A full blots

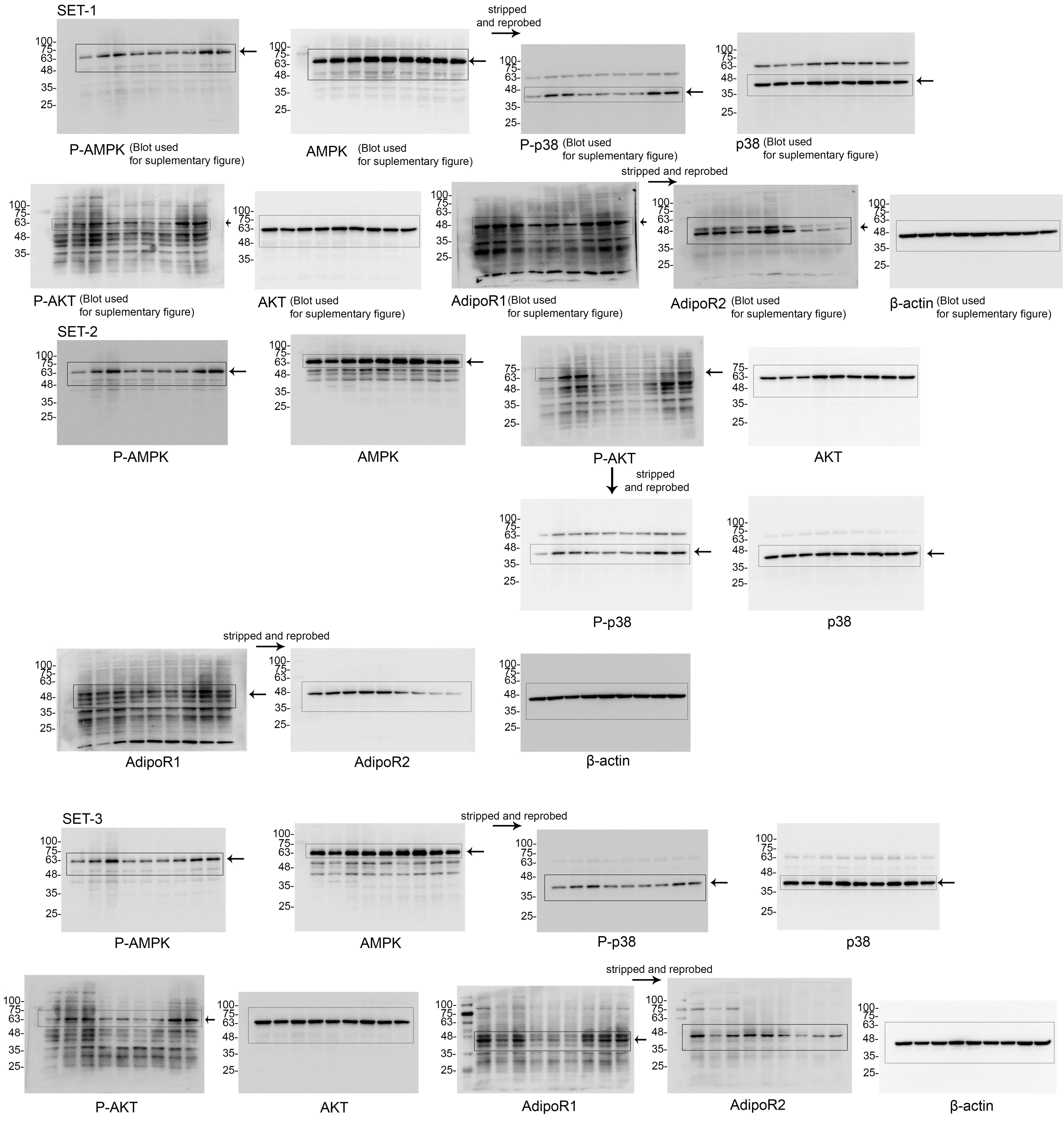

Supplementary Figure S4B full blots

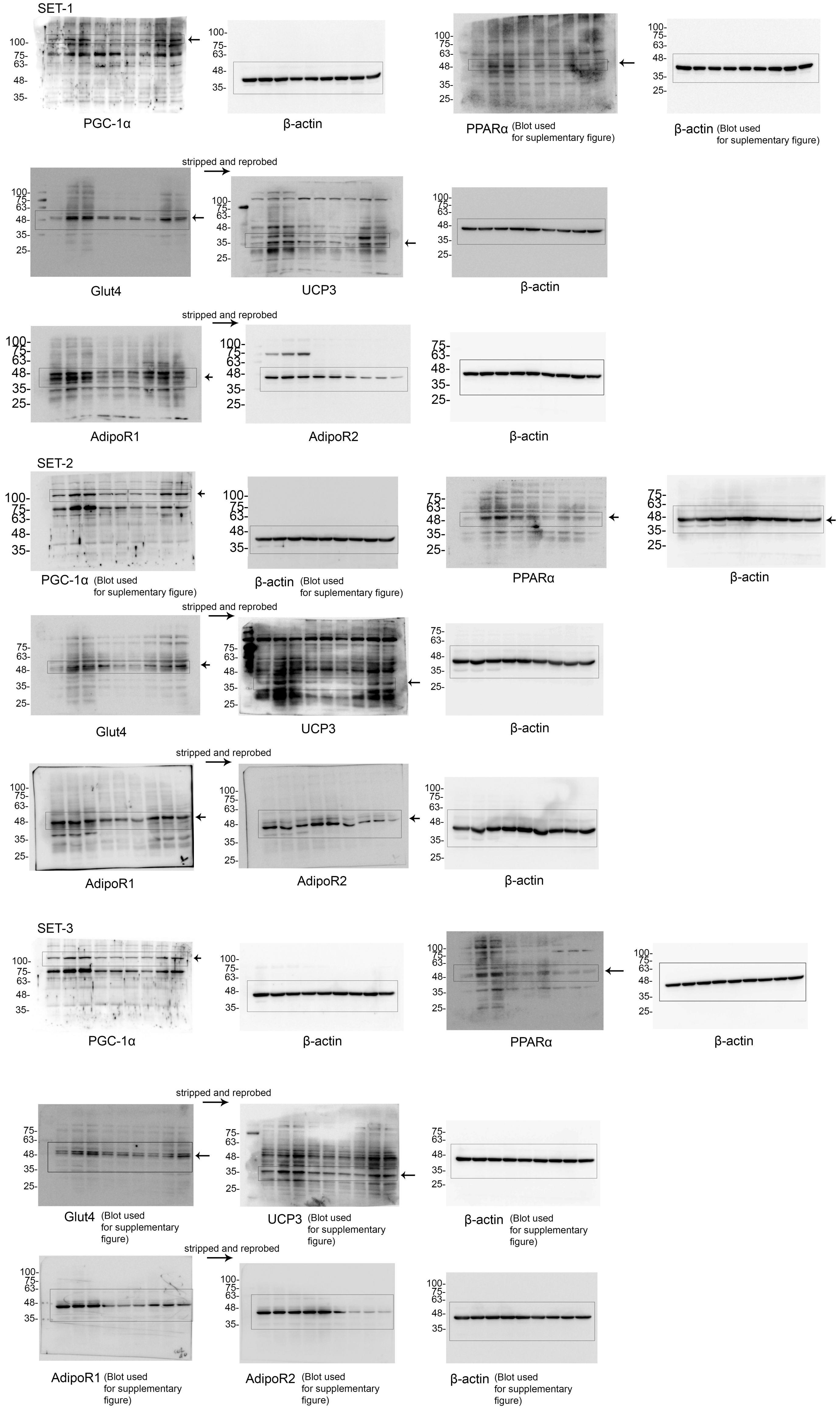

Supplementary Figure S5 full blots

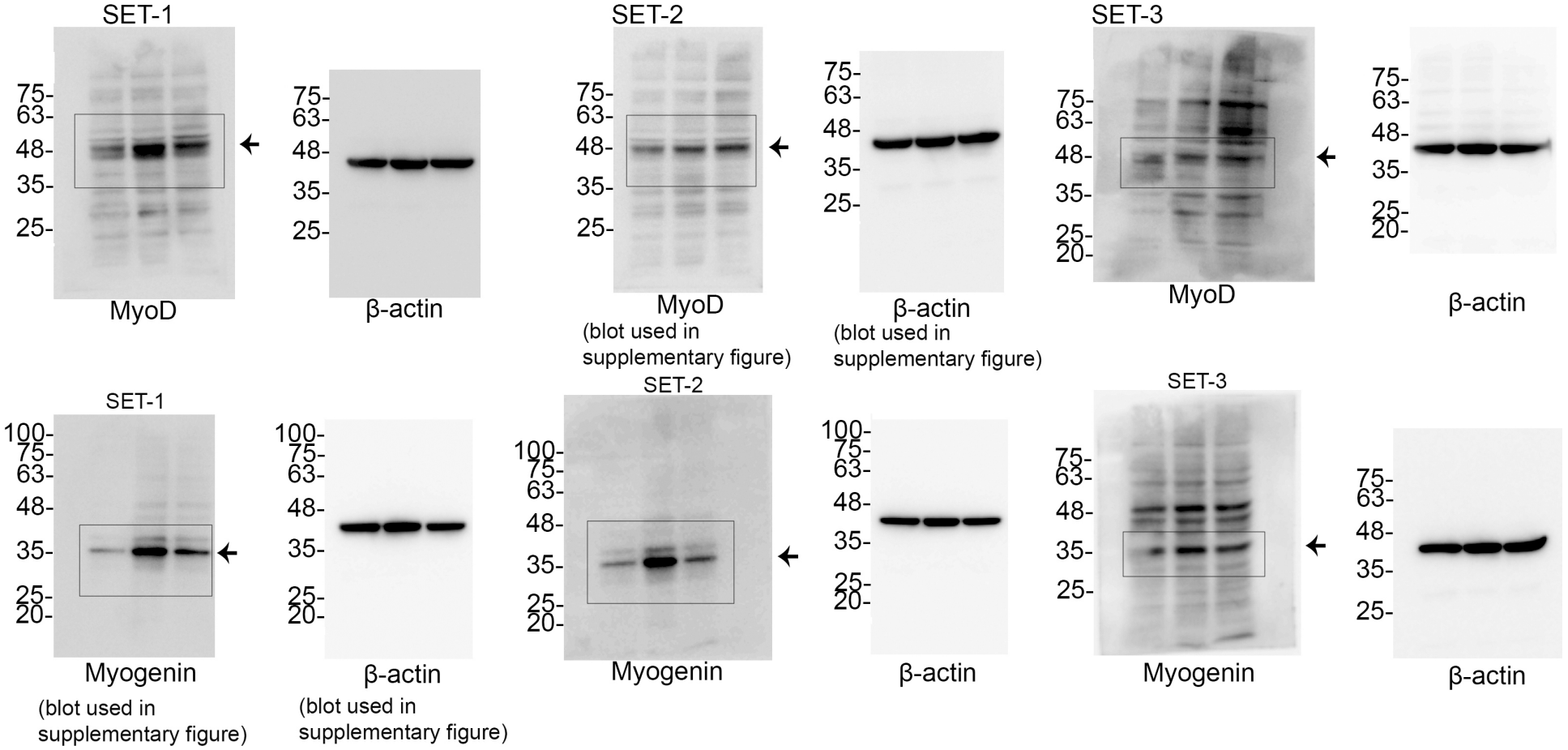

Supplementary Figure S10 full blots

7d Sham (n=6) vs 7d Med (n=6)

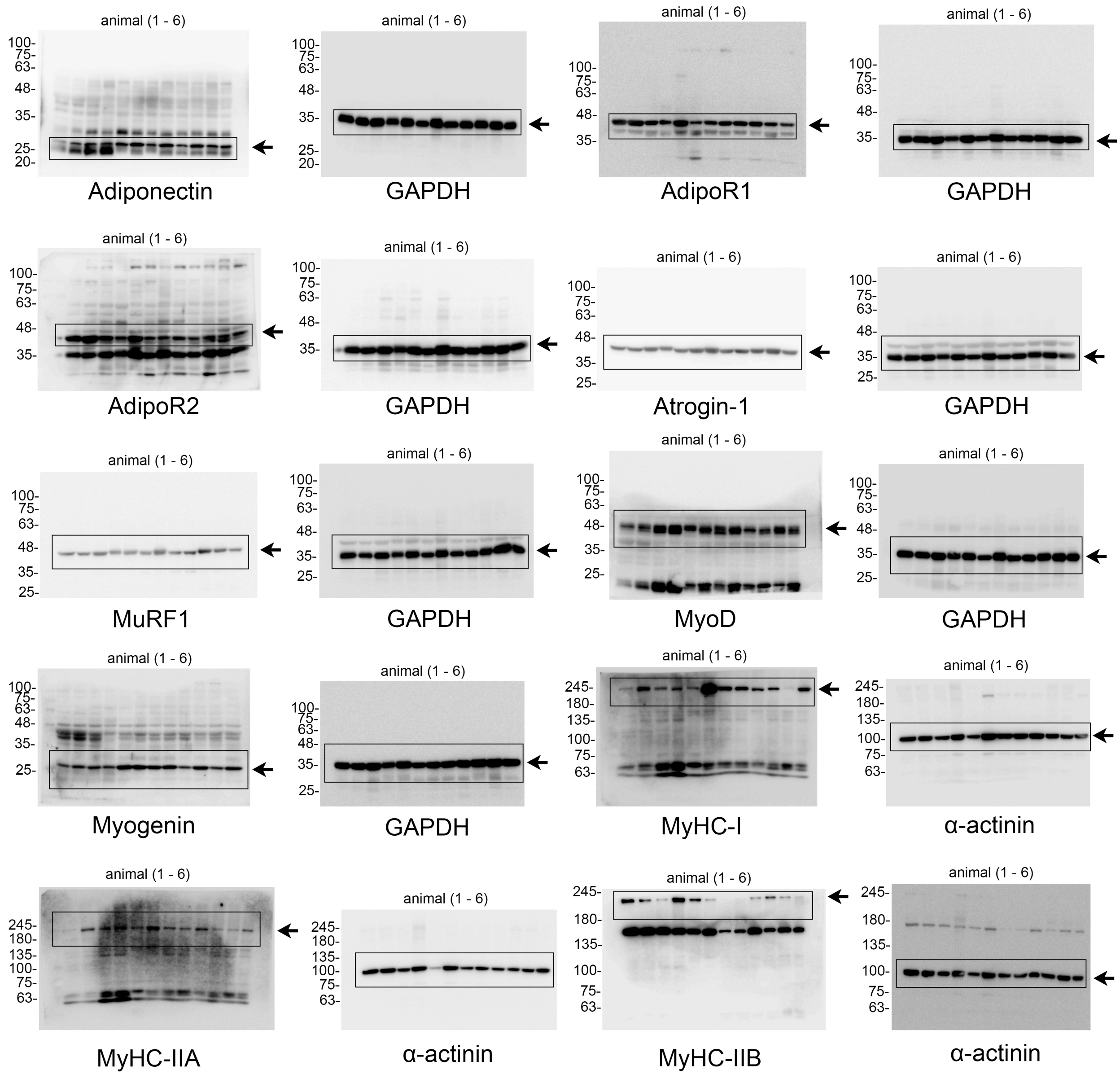

Supplement: Supplementary file 2 [file JCSM-17-e70364-s002.pdf]
